# Synthesis of α,α-Diaryl-α-amino Acid Precursors by Reaction of Isocyanoacetate Esters with *o*-Quinone Diimides

**DOI:** 10.1021/acs.orglett.3c01965

**Published:** 2023-07-24

**Authors:** Adrián Laviós, Pablo Martínez-Pardo, Amparo Sanz-Marco, Carlos Vila, José R. Pedro, Gonzalo Blay

**Affiliations:** † Departament de Química Orgànica, Facultat de Química, Universitat de València, Burjassot E-46100, Spain

## Abstract

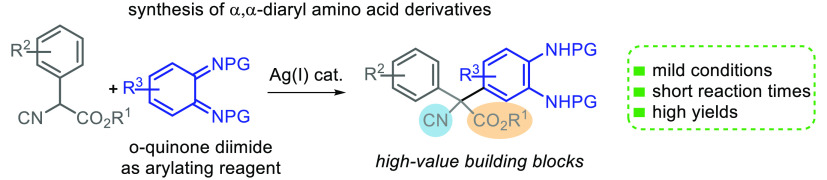

A novel procedure for the synthesis of α,α-diaryl-α-amino acid derivatives has been developed. Silver oxide catalyzes the conjugate addition of α-aryl isocyanoacetates to *o*-quinone diimide, affording the corresponding α,α-diarylisocyano esters in excellent yields and regioselectivities in short reaction times. Acid hydrolysis of the isocyano group provides the corresponding amino acids bearing a diarylated tetrasubstituted carbon atom. The reaction is also amenable to the synthesis of α-alkyl-α-arylisocyano esters, while the reaction with 3-hydroxy *o*-quinone diimides provides 4*H*-benzo[*e*][1,3]oxazines via a conjugate addition/cyclization process.

The α,α-disubstituted α-amino acids have attracted considerable attention in the fields of synthetic, biological, and medicinal chemistry.^1^ They are also found in nature, either in their free form or as constituents of biologically active natural products, exhibiting enzyme inhibition, ion channel blocking, anti-inflammatory, antitumor, or antibiotic properties, among others.^2^ The introduction of two substituents at the α-position of the amino acid imposes conformational constraints and increased steric hindrance with respect to related α-monosubstituted amino acids. For this reason, these amino acids having a tetrasubstituted α-carbon atom have been used in the design and synthesis of new foldamers and peptides with increased chemical stability and lipophilicity as well as restricted conformational flexibility that exhibit enhanced resistance to chemical and enzymatic degradation.^3^ Usually, α,α-dialkyl-substituted amino acids favor the stabilization of helical secondary structures,^4^ while α,α-diaryl substitution often induces extended geometries.^5^

Therefore, the synthesis of α,α-disubstituted α-amino acids and their derivatives has attracted great interest from organic chemists.^1c,1e,6^ Although several methods for the synthesis of α-alkyl quaternary amino acid derivatives have been described, including arylation of 4-alkyl azlactones with diaryliodonium bromides and silver salts,^7^ only a few synthetic approaches for α,α-diaryl-α-amino acid derivatives have been developed.

The most extended strategy involves the nucleophilic arylation of cyclic or acyclic α-ketoimino esters through Friedel–Crafts reactions or the addition of arylboronic acids or Grignard reagents (Scheme 1a).^8^ Additionally, palladium-catalyzed α-arylation of hydantoin derivatives and azlactones has been reported (Scheme 1b).^9^

**Scheme 1:**
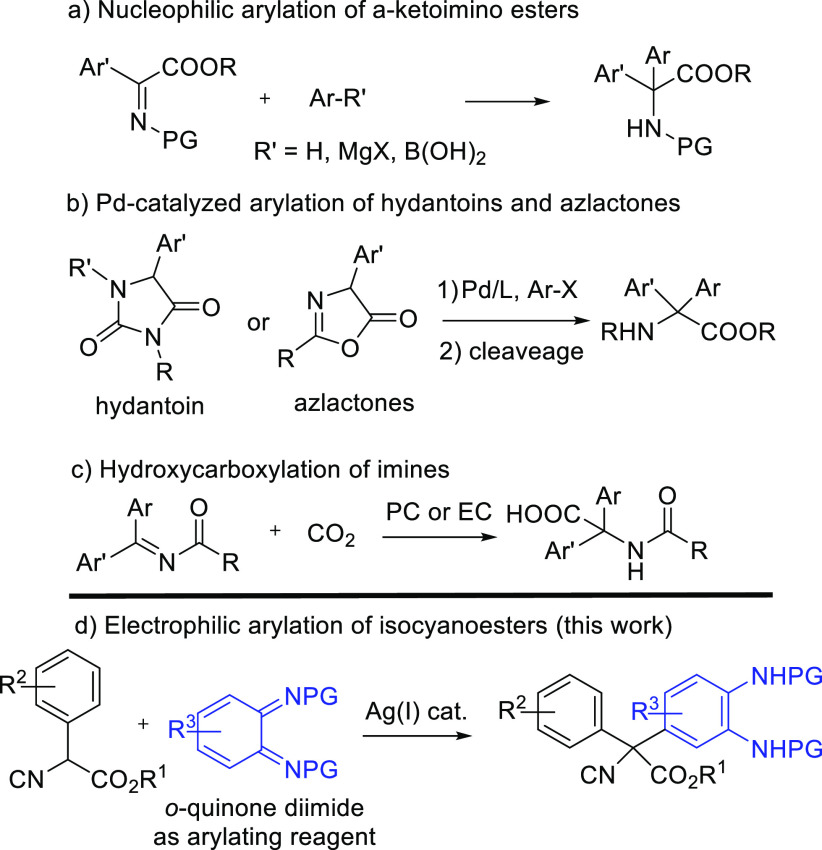
Approaches for the Synthesis of α,α-Diaryl Amino Acid Derivatives

Nucleophilic arylation of azlactones with indoles has been achieved via catalytic aerobic cross-dehydrogenative coupling of azlactones in the presence of FeCl_3_.^10^ Recently, the hydrocarboxylation of *N*-acylimines with carbon dioxide using photocatalytic (PC)^11^ or electrochemical (EC)^12^ procedures to give a variety of α,α-disubstituted amino acids has been developed (Scheme 1c). Despite these advances, there are still some limitations such as limited scope, low yields, or the requirement of precious metals, and the development of new methods for the synthesis of α,α-diaryl-α-amino acids still remains as a major goal in organic synthesis.

On the other hand, α-isocyanoesters can be considered as masked amino acids that show increased α-acidity due to the presence of the electron-withdrawing isocyanide group.^13^ α-Deprotonation of these compounds leads to enolates that behave as formal 1,3-dipoles^14^ or that can react with a variety of carbon electrophiles to give α-substituted-α-isocyano esters via alkylation reactions.^15^ According to this reactivity, we envisioned a straightforward synthesis of α,α-diaryl-α-amino acid derivatives via the electrophilic α-arylation of α-aryl-isocyanoacetates, a methodology that has not been previously reported in the literature that involves the arylation of a highly sterically congested carbon atom.

Herein, we describe the reaction of α-aryl-isocyanoacetates using *o*-quinone diimides as arylating reagents to obtain functionalized α,α-diaryl-α-amino acid derivatives (Scheme 1d). Quinone diimides are important building blocks to synthesize high-value products with biological activity or importance in pharmaceutical chemistry.^16^ These types of compounds show high reactivity driven by rearomatization during the course of the reaction. Thus, *o*-quinone diimides have been used as heterodienes in cycloaddition reactions.^17^ However, their application in arylation reactions involving nucleophilic addition to the carbocycle is almost unknown, and only a few examples with phosphorus, tin, or silicon reagents have been reported in the literature, to the best of our knowledge.^18^

To start our investigation, we decided to test the reaction of methyl 2-isocyano-2-phenylacetate (**1a**) and *o*-benzoquinone diimide **2a** in dichloromethane as the solvent at room temperature (Scheme 2). Product **3aa** was obtained in 70% yield after 24 h with total regioselectivity. Encouraged by this result and considering that Ag^+^ coordinates the isocyano group, thus enhancing the acidity of the α-H^14^ as well as the possibility of electrophilic activation of the imino group,^19^ 5 mol % of Ag_2_O was added to the reaction mixture. In this way, the reaction proceeded smoothly to give compound **3aa** in quantitative yield after 1 h. Cu_2_O also catalyzed the reaction but led to a lower yield.

**Scheme 2:**
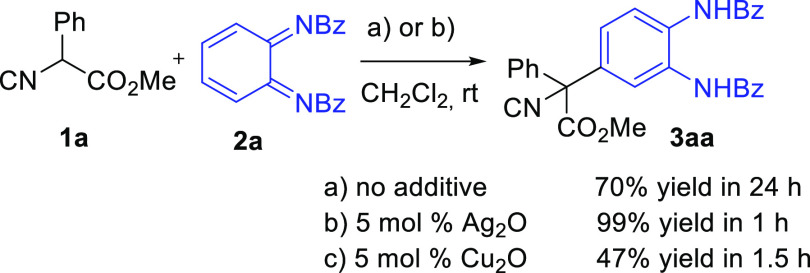
Arylation of Methyl 2-Isocyano-2-phenylacetate (**1a**) with *o*-Quinone Diimide **2a** Reaction conditions: **1a** (0.33 mmol), **2a** (0.25 mmol), CH_2_Cl_2_ (3.0 mL).

Next, we examined the reaction scope (Scheme 3). First, a variety of isocyanoacetates (**1b**–**1d**) were studied. *tert*-Butyl 2-isocyano-2-phenylacetate (**1b**) bearing a bulky alkoxy group was a proper substrate, although the reaction product **3ba** was obtained in a lower yield (73%). The effect of the α-substituent in the isocyanoacetic methyl ester was then studied. Aromatic rings substituted with either electron-donating (MeO) or electron-withdrawing (Cl, NO_2_) groups were tolerated. The presence of a *p-*methoxy group (**1c**) increased the yield (**3ca**, 96%), while isocyanoacetates bearing a halogen (**1d**) or a nitro group in the *para*- (**1e**) or *ortho-* (**1f**) position gave somehow lower but still good yields of compounds **3da** (74%), **3ea** (74%), and **3fa** (88%). Moreover, methyl 2-isocyanopropanoate (**1g**) reacted with **2a** to give **3ga** in 76% yield, indicating that this methodology can be extended to α-alkyl isocyanoacetate esters, providing the corresponding products that are precursors of α-alkyl-α-aryl amino acids.

**Scheme 3:**
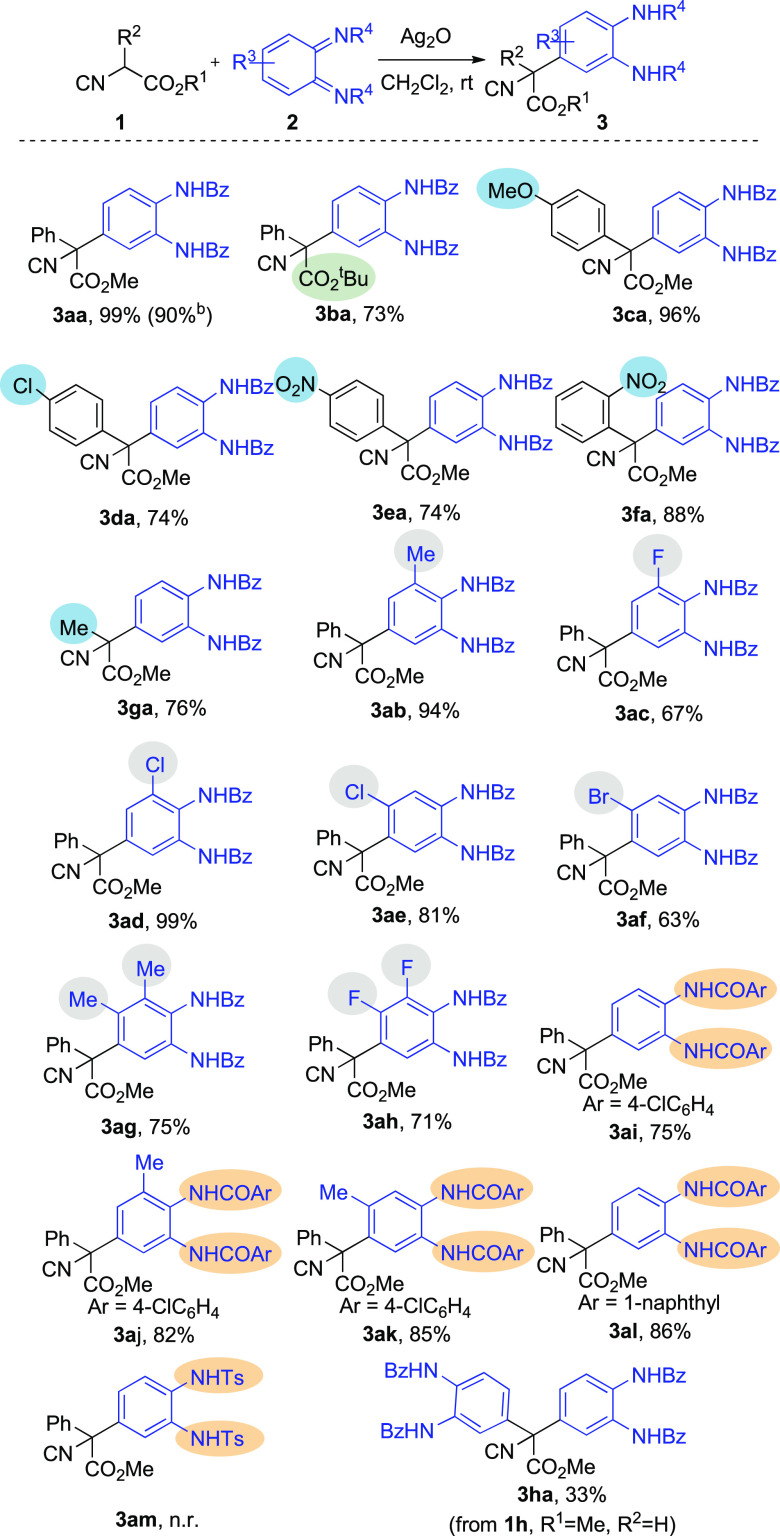
Scope of the Reaction Reaction conditions: **1** (0.33 mmol), **2** (0.25 mmol), Ag_2_O (0.05 mmol), CH_2_Cl_2_ (3 mL), rt, 1 h. Reaction with 2.5 mmol of **2a**.

The scope of the reaction was further examined with a range of substituted *o*-benzoquinone diimides (**2b**–**2m**). Methyl or halogen atoms were allowed at positions 3 (**2b**–**2d**) or 4 (**2e**, **2f**, and **2k**) of the *o*-quinone diimide. The expected arylated products were obtained in fair to excellent yields (67–99%). 3,4-Disubstituted diimides **2g** and **2h** were also suitable reactants that lead to isocyanoacetates arylated with tetrasubstituted phenyl rings **3ag** and **3ah** (71–75%). Finally, the carboxyamido group in compound **2** is also amenable to variation. Diimides **2i**–**2k** bearing a *p*-chlorobenzoyl group and diimide **2l** bearing a 1-naphthylcarboxy group reacted with **1a** to give the corresponding products in good yields (75–86%). However, *N*-tosyl-protected diimide **2m** (R^4^ = Ts) did not react under the reaction conditions. Moreover, 2-unsubstituted methyl isocyanoacetate **1h** reacted with **2a** to give the diarylated product **3ha** in 33% yield. Finally, we proved the scalability and applicability of this protocol by performing a reaction on a 2.5 mmol scale that afforded **3aa** in 90% yield.

It is worth mentioning that when the reaction is carried out with *o-*quinone diimides bearing a 4-hydroxyl group (**2n**), the initial arylation is followed by intramolecular addition of the hydroxyl group to the isocyanide carbon to give benzoxazine derivatives **3an** and **3fn** in good yields (Scheme 4).

**Scheme 4:**
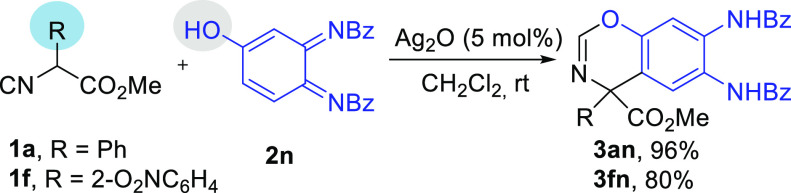
Synthesis of 4*H*-Benzo[*e*][1,3]oxazines

To demonstrate the usefulness of this new methodology in the synthesis of α,α-diaryl-α-amino acids, some of the prepared compounds **3** were subjected to hydrolysis of the isocyano group upon treatment with aqueous hydrochloric acid in MeOH for 4 h. In all of the examples, the expected amino esters **4** were produced in excellent yields (Scheme 5).

**Scheme 5:**
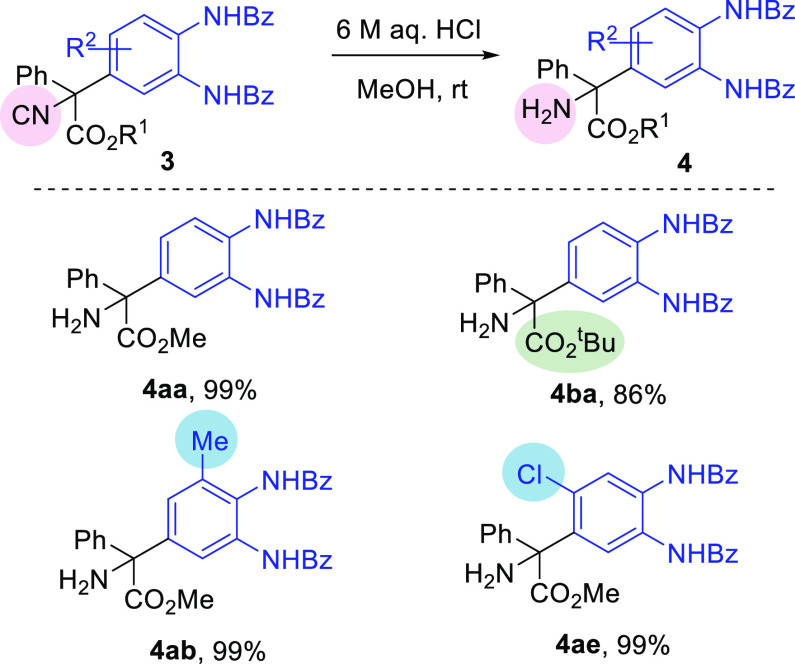
Synthesis of α,α-Diaryl-α-amino Esters **4**

In summary, a novel methodology for the electrophilic arylation of α-substituted-α-isocyanoesters has been developed. Ag_2_O catalyzes the addition of these types of compounds to *o*-quinone diimides, resulting in the formation of the corresponding diaryl (or alkyl-aryl) isocyanoesters. Upon acidic hydrolysis, these compounds are converted into α,α-diaryl-α-amino esters, which are of great interest in medicinal chemistry. The reaction also provides new synthetic access to benzoxazines when 4-hydroxy-*o*-quinone diimides are used.

## Data Availability

The data underlying this study are available in the published article and its Supporting Information

## Supporting Information Available

Experimental procedures, characterization data, and NMR spectra (PDF)
FAIR data, including the primary NMR FID files, for compounds **2**–**4** (ZIP)
