# Supplementary material for: Synthesis of α,α-Diaryl-α-amino Acid Precursors by Reaction of Isocyanoacetate Esters with o-Quinone Diimides
Source: Org Lett. 2023 Jul 24;25(30):5608–12. doi: 10.1021/acs.orglett.3c01965 (PMC10853967; doi:10.1021/acs.orglett.3c01965)
Supplement: Supplementary file 1 — ol3c01965_si_001.pdf [file ol3c01965_si_001.pdf]

Synthesis of α,α-diaryl-α-amino acid precursors by reaction of isocyanoacetate esters with *o*-quinone diimides

Adrián Laviós,[a] Pablo Martínez-Pardo,[a] Amparo Sanz-Marco,[a] Carlos Vila,[a] José R. Pedro[a] and Gonzalo Blay[a,]*

[a] Departament de Química Orgànica, Universitat de València, C/ Dr. Moliner 50, E-46100-Burjassot (València), Spain.
E-mail: gonzalo.blay@uv.es

Table of Contents:

General Experimental Methods S2

Synthesis and characterization data for compounds **2** S2

Synthesis and characterization data for compounds **3** S6

Synthesis of compound **3aa** at 2.5 mmol-scale S13

Synthesis and characterization data for compounds **4** S13

References S14

NMR spectra S15

Additional experiments S54

General Experimental Methods

Unless otherwise stated, starting materials were obtained from commercial sources and used without previous purification. Reactions were monitorized by TLC analysis using Merck Silica Gel 60 F254 thin layer plates. Flash column chromatography was performed on Merck silica gel 60, 0.040-0.063 mm. Melting points were determined in capillary tubes. NMR spectra were recorded at 300 MHz, 400 MHz or 500 MHz for ^{1}H, at 75 MHz or 101 MHz for ^{13}C. Residual non-deuterated solvent signals were used as internal standard (7.26 ppm for ^{1}H and 77.16 ppm for ^{13}C in $CDCl_3$, and 2.50 ppm for ^{1}H and 39.52 ppm for ^{13}C in DMSO-d_6). Chemical shifts are given in ppm. The carbon type was determined by DEPT experiments. High resolution mass spectra (ESI) were recorded on a Q-TOF spectrometer equipped with an electrospray source with a capillary voltage of 3.3 kV (ESI). α-Substituted isocyanoacetates **1** were synthesized according to a literature procedure.[1]

Synthesis and characterization data for compounds 2[2]

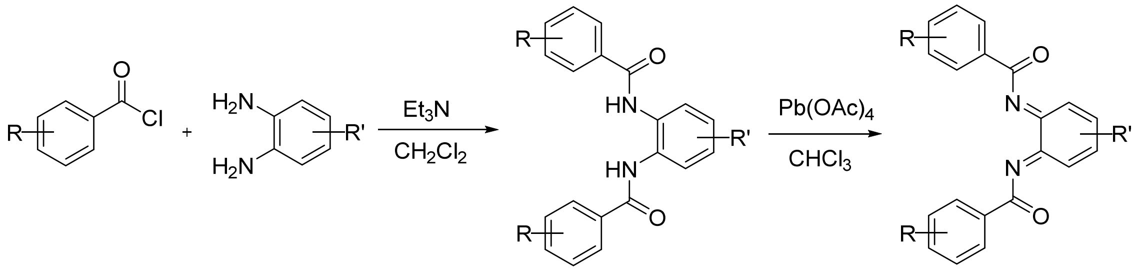

2

Step 1: A solution of benzoyl chloride (2.81 g, 2.32 mL, 20 mmol, 2 equiv) in CH_2Cl_2 (50 mL, 0.4 M) was added to an ice-cooled solution of 1,2-phenylenediamine derivative (10 mmol, 1 equiv) and triethylamine (2.53 g, 3.5 mL, 25 mmol, 2.5 equiv) in CH_2Cl_2 (50 mL, 0.2 M). The reaction was allowed to warm to room temperature and it was stirred at rt until consumption of diamine (TLC). Once completed, the white solid product was collected by filtration, washed with a small amount of CH_2Cl_2 (15 mL) and no further purification was needed.

Step 2: $Pb(OAc)_4$ (3.10 g, 7 mmol, 1.4 equiv) was added at 0 °C in portions to a suspension of *N,N'*-(1,2-phenylene)dibenzamide derivative (5 mmol, 1 equiv) in $CHCl_3$ (100 mL, 0.05 M). The mixture was stirred at 40 ºC until completion of the reaction (TLC analysis). The suspension was then filtered over celite and washed with 50 mL of $CHCl_3$. The filtrate and washings were placed in a separatory funnel and washed with 50 mL of 5% aqueous $NaHCO_3$ and then with 50 mL of water. The organic layers were dried over Na_2SO_4, filtered and concentrated under reduced pressure to afford the crude product. Recrystallization from hexane/diethyl ether gave pure compounds **2**.

N,N'-(Cyclohexa-3,5-diene-1,2-diylidene)dibenzamide (2a)[3]

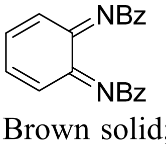

1.07 g (68%) of **2a** were obtained from 1.6 g of *N,N'*-(1,2-phenylene)dibenzamide.

Brown solid; m.p. 123.9-124.9 ºC; ^{1}H NMR (300 MHz, $CDCl_3$) δ 7.72 (4H, d, J = 7.1 Hz), 7.59-7.48 (2H, m), 7.38 (4H, m), 6.78 (2H, m), 6.57 (2H, m). ^{13}C NMR (75 MHz, $CDCl_3$) δ 180.0 (C), 134.4 (CH), 133.4 (CH), 132.0 (C), 129.0 (CH), 128.7 (CH).

***N,N'*-(3-Methylcyclohexa-3,5-diene-1,2-diylidene)dibenzamide (2b)**

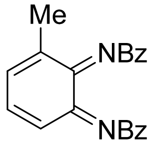

1.1 g (67%) of **2b** were obtained from 1.7 g of *N,N'*-(3-methyl-1,2-phenylene)dibenzamide.

Orange solid; m.p. 125-127 ºC; ^{1}H NMR (300 MHz, $CDCl_3$) δ 7.80 (2H, dd, *J* = 8.4, 1.4 Hz), 7.60 (2H, dd, *J* = 8.4, 1.4 Hz), 7.56-7.44 (2H, m), 7.42-7.31 (4H, m), 6.72-6.57 (2H, m), 6.26 (1H, m), 2.25 (3H, s). ^{13}C NMR (75 MHz, $CDCl_3$) δ 180.2 (C), 179.2 (C), 154.6 (C), 150.9 (C), 139.5 (C), 136.0 (CH), 133.8 (CH), 132.6 (CH), 132.18 (C), 132.15 (C), 131.2 (CH), 129.6 (CH), 128.64 (CH), 128.57 (CH), 128.2 (CH), 121.0 (CH), 18.0 (CH_3). HRMS (ESI) *m/z*: 329.1290 $[M+H]^+$, $C_{21}H_{17}N_2O_2^+$ required 329.1285.

***N,N'*-(3-Fluorocyclohexa-3,5-diene-1,2-diylidene)dibenzamide (2c)**

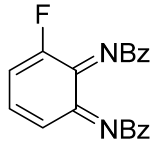

1.31 g (79%) of **2c** were obtained from 1.67 g of *N,N'*-(3-fluoro-1,2-phenylene)dibenzamide.

Brown solid; m.p. 72.8-73.5 ºC; ^{1}H NMR (300 MHz, $CDCl_3$) δ 7.78 (2H, d, *J* = 7.3 Hz, Ar), 7.68 – 7.61 (2H, m), 7.59 – 7.50 (3H, m), 7.44 – 7.35 (3H, m), 6.69 (1H, ddd, *J* = 9.9, 7.1, 5.9 Hz), 6.47 (1H, ddd, *J* = 9.3, 7.1, 1.0 Hz), 6.30 (1H, d, *J* = 9.9 Hz). ^{13}C NMR (125 MHz, $CDCl_3$) δ 179.3 (C), 179.0 (C), 134.0 (CH), 133.4 (CH), 131.9 (C), 131.6 (C), 129.5 (CH), 128.9 (CH), 128.9 (CH), 128.7 (CH), 113.6 (d, $^2J_{C\text{-}F}$ = 20.3 Hz, CH); ^{19}F NMR (282 MHz, $CDCl_3$) δ -123.4; HRMS (ESI) *m/z*: 333.1033 $[M+H]^+$, $C_{20}H_{14}FN_2O_2^+$ required 333.1034.

***N,N'*-(3-Chlorocyclohexa-3,5-diene-1,2-diylidene)dibenzamide (2d)**

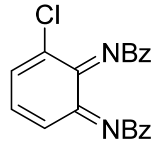

1.44 g (82%) of **2d** were obtained from 1.8 g of *N,N'*-(3-chloro-1,2-phenylene)dibenzamide.

Orange solid; m.p. 139-141 ºC; ^{1}H NMR (300 MHz, $CDCl_3$) δ 7.81 (2H, dd, *J* = 8.4, 1.3 Hz), 7.60 (2H, dd, *J* = 8.4, 1.3 Hz), 7.58-7.49 (2H, m), 7.44-7.34 (4H, m), 7.03 (1H, dd, *J* = 6.8, 1.0 Hz), 6.66 (1H, dd, *J* = 9.8, 6.8 Hz), 6.31 (1H, dd, *J* = 9.8, 1.0 Hz). ^{13}C NMR (75 MHz, $CDCl_3$) δ 179.3 (C), 178.6 (C), 150.5 (C), 150.2 (C), 134.9 (C), 134.2 (CH), 134.1 (CH), 133.1 (CH), 132.0 (CH), 131.8 (C),131.7 (C), 129.6 (CH), 128.8 (CH), 128.7 (CH), 128.3 (CH), 122.3 (CH). HRMS (ESI) *m/z*: 349.0748 $[M+H]^+$, $C_{20}H_{14}ClN_2O_2^+$ required 349.0738.

***N,N'*-(4-Chlorocyclohexa-3,5-diene-1,2-diylidene)dibenzamide (2e)**[3]

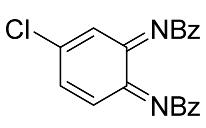

1.32 g (75%) of **2e** were obtained from 1.8 g of *N,N'*-(4-chloro-1,2-phenylene)dibenzamide.

^{1}H NMR (300 MHz, $CDCl_3$) δ 7.75-7.62 (4H, m), 7.54 (2H, t, *J* = 7.4 Hz), 7.45-7.33 (4H, m), 6.75 (1H, dd, *J* = 10.1, 2.0 Hz), 6.68 (1H, d, *J* = 10.0 Hz), 6.67 (1H, s). ^{13}C NMR (75 MHz, $CDCl_3$) δ 179.3 (C), 179.0 (C), 136.4 (CH), 133.7 (CH), 133.5 (CH), 131.9 (C), 131.6 (C), 129.2 (CH), 128.8 (CH), 128.7 (CH). HRMS (ESI) *m/z*: 349.0742 $[M+H]^+$, $C_{20}H_{14}ClN_2O_2^+$ required 349.0738.

N,N'-(4-Bromocyclohexa-3,5-diene-1,2-diylidene)dibenzamide (2f)

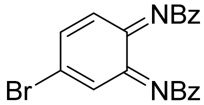

1.21 g (87%) of **2f** were obtained from 1.4 g of *N,N'*-(4-bromo-1,2-phenylene)dibenzamide.

Brown solid; m.p. 71.7-72.5 ºC; ^{1}H NMR (300 MHz, $CDCl_3$) δ 7.75 – 7.64 (4H, m), 7.59 – 7.49 (3H, m), 7.45 – 7.31 (4H, m), 6.90 – 6.83 (1H, m), 6.57 (1H, d, *J* = 10.1 Hz). ^{13}C NMR (75 MHz, $CDCl_3$) δ 179.4 (C), 179.2 (C), 138.5 (CH), 133.9 (CH), 133.6 (CH), 131.9 (C), 131.8 (C), 129.4 (CH), 129.0 (CH), 128.9 (CH); HRMS (ESI) *m/z*: 393.0229 $[M+H]^+$, $C_{20}H_{14}BrN_2O_2^+$ required 393.0233.

N,N'-(3,4-Dimethylcyclohexa-3,5-diene-1,2-diylidene)dibenzamide (2g)

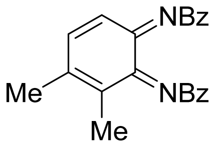

0.47 g (70%) of **2g** were obtained from 0.67 g of *N,N'*-(3,4-dimethyl-1,2-phenylene)dibenzamide.

Brown oil; this compound was highly unstable and was just characterized by ^{1}H NMR (300 MHz, $CDCl_3$) δ 7.82 – 7.77 (2H, m), 7.64 – 7.56 (2H, m), 7.55 – 7.45 (2H, m), 7.41 – 7.31 (4H, m), 2.21 (3H, s), 2.12 (3H, s).

N,N'-(3,4-difluorocyclohexa-3,5-diene-1,2-diylidene)dibenzamide (2h)

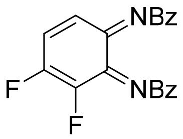

1.2 g (67%) of **2h** were obtained from 1.8 g of *N,N'*-(3,4-difluoro-1,2-phenylene)dibenzamide.

Orange solid; ^{1}H NMR (300 MHz, $CDCl_3$) δ 8.02 – 7.88 (1H, m), 7.81 – 7.70 (2H, m), 7.69 – 7.60 (2H, m), 7.59 – 7.50 (2H, m), 7.45 – 7.33 (3H, m), 6.76 (1H, ddd, *J* = 10.6, 8.7, 7.8 Hz), 6.50 – 6.33 (1H, m); ^{13}C NMR (75 MHz, $CDCl_3$) δ 178.9 (C), 178.6 (C), 167.0 (C), 166.2 (C), 134.2 (CH), 133.6 (CH), 133.0 (CH), 132.4 (CH), 131.6 (C), 129.4 (CH), 129.1 (CH), 129.0 (CH), 128.9 (CH), 128.8 (CH), 127.8 (CH), 127.6 (CH); ^{19}F NMR (282 MHz, $CDCl_3$) δ -139.94 (d, *J* = 21.4 Hz), -144.15 (d, *J* = 21.2 Hz); HRMS (ESI) *m/z*: 351.0940 $[M+H]^+$, $C_{20}H_{12}F_2N_2O_2^+$ required 351.0943.

N,N'-(Cyclohexa-3,5-diene-1,2-diylidene)bis(4-chlorobenzamide) (2i)

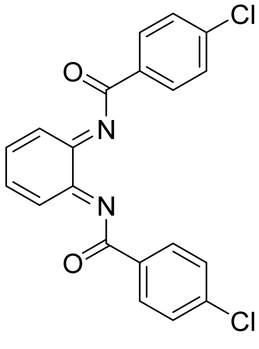

1.32 g (69%) of **2i** were obtained from 1.9 g of *N,N'*-(1,2-phenylene)bis(4-chlorobenzamide).

Orange solid; m.p. 135-137 ºC; ^{1}H NMR (300 MHz, $CDCl_3$) δ 7.66 (4H, d, *J* = 8.6 Hz), 7.37 (4H, d, *J* = 8.6 Hz), 6.81 (2H, dd, *J* = 7.7, 3.3 Hz), 6.54 (2H, dd, *J* = 7.7, 3.3 Hz). ^{13}C NMR (75 MHz, $CDCl_3$) δ 178.4 (C), 152.7 (C), 140.0 (C), 134.9 (CH), 130.3 (CH), 129.1 (CH). HRMS (ESI) *m/z*: 383.0355 $[M+H]^+$, $C_{20}H_{13}Cl_2N_2O_2^+$ required 383.0349.

N,N'-(3-Methylcyclohexa-3,5-diene-1,2-diylidene)bis(4-chlorobenzamide) (2j)

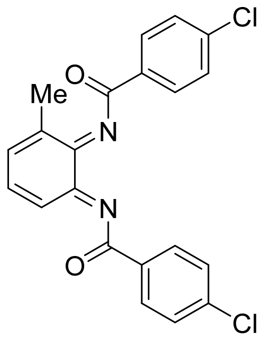

1.5 g (75%) of **2j** were obtained from 2.0 g of *N,N'*-(3-methyl-1,2-phenylene)bis(4-chlorobenzamide).

Orange solid; m.p. 136-138 ºC; ^{1}H NMR (300 MHz, $CDCl_3$) δ 7.71 (2H, d, *J* = 8.7 Hz), 7.57 (2H, d, *J* = 8.7 Hz), 7.37 (2H, d, *J* = 5.7 Hz), 7.35 (2H, d, *J* = 5.7 Hz), 6.71-6.62 (2H, m), 6.22-6.11 (1H, m), 2.23 (3H, s). ^{13}C NMR (75 MHz, $CDCl_3$) δ 179.2 (C), 177.9 (C), 155.1 (C), 151.3 (C), 140.5 (C), 139.5 (C), 139.0 (C), 136.5 (CH), 131.5 (CH), 131.0 (CH), 130.6 (C), 130.5 (CH), 129.5 (CH), 129.1 (CH), 129.0 (CH), 120.9 (CH), 17.9 (CH_3). HRMS (ESI) *m/z*: 397.0507 $[M+H]^+$, $C_{21}H_{15}Cl_2N_2O_2^+$ required 397.0505.

N,N'-(4-Methylcyclohexa-3,5-diene-1,2-diylidene)bis(4-chlorobenzamide) (2k)

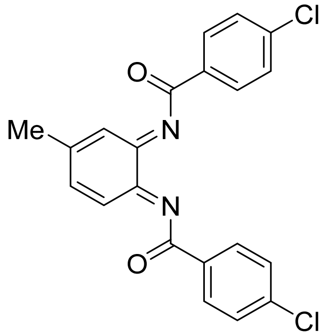

1.46 g (73%) of **2k** were obtained from 2.0 g of *N,N'*-(3-methyl-1,2-phenylene)bis(4-chlorobenzamide).

Orange solid; m.p. 196-198 ºC; ^{1}H NMR (300 MHz, $CDCl_3$) δ 7.68 (2H, d, *J* = 8.7 Hz), 7.64 (2H, d, *J* = 8.7 Hz), 7.40-7.34 (4H, m), 6.68 (1H, dd, *J* = 9.8. 1.8 Hz), 6.60 (1H, bd, *J* = 9.9 Hz), 6.28 (1H, bs), 2.09 (3H, d, *J* = 1.8 Hz). ^{13}C NMR (75 MHz, $CDCl_3$) δ 178.7 (C), 178.5 (C), 140.0 (C), 139.7 (C), 138.5 (CH), 130.7 (C), 130.6 (CH), 130.5 (C), 130.1 (CH), 129.1 (CH), 22.3 (CH_3). HRMS (ESI) *m/z*: 397.0508 $[M+H]^+$, $C_{21}H_{15}Cl_2N_2O_2^+$ required 397.0505.

N,N'-(Cyclohexa-3,5-diene-1,2-diylidene)bis(1-naphthamide) (2l)

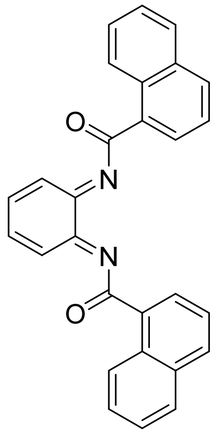

1.77g (84%) of **2l** were obtained from 2.2 g of *N,N'*-(1,2-phenylene)bis(1-naphthamide).

Orange solid; m.p. 140-142 ºC; ^{1}H NMR (300 MHz, $CDCl_3$) δ 8.96 (2H, dd, *J* = 6.3, 3.6 Hz), 7.96 (2H, d, *J* = 8.3 Hz), 7.79 (2H, dd, *J* = 6.3, 3.6 Hz), 7.65 (2H, br d, *J* = 6.8 Hz), 7.40 (4H, dd, *J* = 6.4, 3.4 Hz), 7.33 (2H, t, *J* = 7.8 Hz), 6.75 (2H, dd, *J* = 7.7, 3.4 Hz), 6.61 (2H, bd, *J* = 7.7 Hz). ^{13}C NMR (75 MHz, $CDCl_3$) δ 181.1 (C), 151.2 (C), 134.3 (CH), 133.9 (CH), 133.8 (C), 131.1 (C), 130.5 (C), 128.4 (CH), 128.1 (CH), 126.3 (CH), 125.8 (CH), 124.2 (CH). HRMS (ESI) *m/z*: 415.1441 $[M+H]^+$, $C_{28}H_{19}N_2O_2^+$ required 415.1441.

N,N'-(Cyclohexa-3,5-diene-1,2-diylidene)bis(4-methylbenzenesulfonamide) (2m)

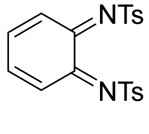

1.57 g (76%) of **2m** were obtained from 2.1 g of *N,N'*-(1,2-phenylene)bis(4-methylbenzenesulfonamide).[4]

Brown solid; m.p. 94-95 ºC; ^{1}H NMR (400 MHz, $CDCl_3$) δ 7.61 – 7.53 (4H, m), 7.24 – 7.16 (4H, m), 7.04 – 6.92 (4H, m), 2.37 (6H, s); ^{13}C NMR (125 MHz, $CDCl_3$) δ 144.3 (C), 135.6 (C), 130.9 (C), 129.8 (CH), 127.7 (CH), 127.4 (CH), 126.1 (CH), 21.7

(CH_3). HRMS (ESI) *m/z*: (ESI) *m/z*: 417.0940 $[M+H_2+H]^+$, $C_{20}H_{21}N_2O_4S2^+$ required 417.0937.

***N,N'*-(4-Hydroxycyclohexa-3,5-diene-1,2-diylidene)dibenzamide (2n)**

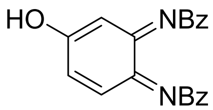

1.29 g (77%) of **2n** were obtained from 1.7 g of *N,N'*-(4-hydroxy-1,2-phenylene)dibenzamide.

White solid; m.p. 169-171 ºC; ^{1}H NMR (300 MHz, $CDCl_3$) δ 9.32 (1H, bs), 7.94-7.87 (4H, m), 7.86 (1H, d, *J* = 2.1 Hz), 7.67 (1H, t, *J* = 7.4 Hz), 7.60 (1H, t, *J* = 7.3 Hz), 7.56-7.45 (4H, m), 6.84 (1H, d, *J* = 10.1 Hz), 6.51 (1H, dd, *J* = 10.1, 2.1 Hz). ^{13}C NMR (75 MHz, $CDCl_3$) δ 187.0 (C), 178.9 (C), 165.5 (C), 153.4 (C), 140.0 (C), 135.4 (CH), 134.7 (CH), 133.3 (C), 132.9 (CH), 131.3 (C), 129.6 (CH), 129.11 (CH), 129.07 (CH), 127.9 (CH), 127.2 (CH), 115.4 (CH). HRMS (ESI) *m/z*: 331.1081 $[M+H]^+$, $C_{20}H_{15}N_2O_3^+$ required 331.1077.

Synthesis and characterization data for compounds 3

α-Substituted isocyanoacetate **1** (0.33 mmol, 1.3 equiv) was added to a solution of *o*-benzoquinone diimide **2** (0.25 mmol, 1 equiv) and silver oxide (3 mg, 0.0125 mmol, 0.05 equiv) in CH_2Cl_2 (3 mL). The mixture was stirred at rt until consumption of diimine **2** (TLC). Then, the reaction mixture was purified by flash column chromatography.

Methyl 2-(3,4-bis(benzamido)phenyl)-2-isocyano-2-phenylacetate (3aa)

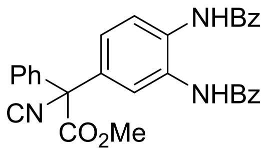

121 mg (99%) of **3aa** were obtained from **1a** (57.8 mg, 0.33 mmol) and **2a** (78.6 mg, 0.25 mmol), after flash chromatography eluting with hexane/EtOAc (6:4).

Brown solid; m.p. 102-105 ºC; ^{1}H NMR (300 MHz, $CDCl_3$) δ 9.48 (1H, bs), 9.33 (1H, bs), 8.00-7.92 (4H, m), 7.65 (1H, d, *J* = 8.6 Hz), 7.60 (1H, d, *J* = 2.3 Hz), 7.51 (2H, dd, *J* = 7.2, 1.6 Hz), 7.44 (4H, td, *J* = 7.2, 1.6 Hz), 7.31-7.23 (3H, m), 7.19 (2H, dd, *J* = 8.2, 1.6 Hz), 7.09 (1H, dd, *J* = 8.6. 2.3 Hz), 3.73 (3H, s). ^{13}C NMR (75 MHz, $CDCl_3$) δ 167.3 (C), 166.8 (C), 166.5 (C), 161.9 (C), 136.7 (C), 134.0 (C), 133.3 (C), 133.1 (C), 132.3 (CH), 132.2 (CH), 131.9 (C), 130.6 (C), 129.1 (CH), 128.72 (CH), 128.67 (CH), 127.7 (CH), 127.6 (CH), 126.9 (CH), 126.0 (CH), 125.4 (CH), 124.2 (CH), 73.2 (C), 54.2 (CH_3). HRMS (ESI) *m/z*: 490.1757 $[M+H]^+$, $C_{30}H_{24}N_3O_4^+$ required 490.1761.

***tert*-Butyl 2-(3,4-bis(benzamido)phenyl)-2-isocyano-2-phenylacetate (3ba)**

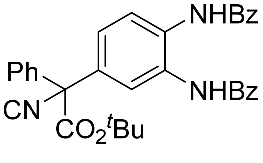

97 mg (73%) of **3ba** were obtained from **1b** (71.7 mg, 0.33 mmol) and **2a** (78.6 mg, 0.25 mmol), after flash chromatography eluting with hexane/EtOAc (7:3).

Pink solid; m.p. 125-127 ºC; ^{1}H NMR (300 MHz, $CDCl_3$) δ 9.38 (1H, bs), 8.98 (1H, bs), 7.97-7.93 (4H, m), 7.74 (1H, d, *J* = 8.6 Hz), 7.59 (1H, d, *J* = 2.3 Hz), 7.55-7.41 (6H, m), 7.34-7.27 (4H, m), 7.31 (1H, dd, *J* = 8.4, 2.1 Hz), 1.45 (9H, s). ^{13}C NMR (75 MHz, $CDCl_3$) δ 166.8 (C), 166.3 (C), 165.5 (C), 161.1 (C), 137.3 (C), 134.6 (C), 133.5 (C), 133.2 (C), 132.4 (CH), 132.2 (CH), 131.9 (C), 130.3 (C), 128.9 (CH),

128.80 (CH), 128.76 (CH), 128.6 (CH), 127.6 (CH), 127.5 (CH), 127.0 (CH), 125.8 (CH), 125.6 (CH), 124.3 (CH), 85.2 (C), 73.7 (C), 27.6 (CH_3). HRMS (ESI) *m/z*: 549.2496 $[M+NH_4]^+$, $C_{33}H_{33}N_4O_4^+$ required 549.2496.

Methyl 2-(3,4-bis(benzamido)phenyl)-2-isocyano-2-(4-methoxyphenyl)acetate (3ca)

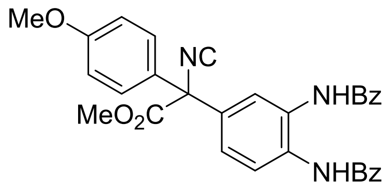

124 mg (96%) of **3ca** were obtained from **1c** (70.0 mg, 0.25 mmol) and **2a** (78.6 mg, 0.25 mmol), after flash chromatography eluting with hexane/EtOAc (6:4).

Orange oil; ^{1}H NMR (300 MHz, $CDCl_3$) δ 9.48 (1H, br s), 9.31 (1H, br s), 8.02 – 7.89 (5H, m), 7.66 (1H, d, *J* = 8.7 Hz), 7.58 (1H, d, *J* = 2.3 Hz), 7.55 – 7.39 (6H, m), 7.14 – 7.03 (3H, m), 6.74 (1H, d, *J* = 8.9 Hz), 3.77 (3H, s), 3.73 (3H, s); ^{13}C NMR (75 MHz, $CDCl_3$) δ 167.7 (C), 167.0 (C), 166.6 (C), 161.6 (C), 160.0 (C), 134.4 (C), 133.5 (C), 133.2 (C), 132.5 (CH), 132.4 (CH), 132.1 (C), 130.7 (C), 129.0 (C), 128.9 (CH), 128.5 (CH), 127.8 (CH), 127.7 (CH), 126.1 (CH), 125.5 (CH), 124.3 (CH), 114.1 (CH), 72.9 (C), 55.4 (CH_3), 54.3 (CH_3); HRMS (ESI) *m/z*: 537.2074 $[M+NH_4]^+$, $C_{31}H_{29}N_4O_5^+$ required 537.2132.

Methyl 2-(3,4-bis(benzamido)phenyl)-2-(4-chlorophenyl)-2-isocyanoacetate (3da)

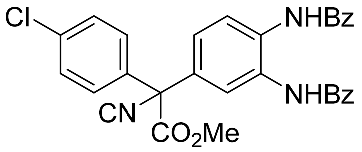

97 mg (74%) of **3da** were obtained from **1d** (69.2 mg, 0.33 mmol) and **2a** (78.6 mg, 0.25 mmol), after flash chromatography eluting with DCM/EtOAc (95:5).

Yellow solid; m.p. 145-147 ºC; ^{1}H NMR (300 MHz, DMSO-*d*$_6$) δ 10.22 (1H, br s), 10.11 (1H, br s), 7.98-7.91 (4H, m), 7.83 (1H, d, *J* = 8.6 Hz), 7.73 (1H, d, *J* = 2.4 Hz), 7.63-7.48 (8H, m), 7.44 (2H, d, *J* = 8.7 Hz), 7.28 (1H, dd, *J* = 8.6, 2.5 Hz), 3.91 (3H, s). ^{13}C NMR (75 MHz, DMSO-*d*$_6$) δ 166.8 (C), 165.8 (C), 165.5 (C), 161.8 (C), 135.6 (C), 134.3 (C), 134.1 (C), 133.9 (C), 133.2 (C), 132.2 (C), 132.0 (CH), 131.5 (C), 129.1 (CH), 129.0 (CH), 128.62 (CH), 128.55 (CH), 127.7 (CH), 127.6 (CH), 126.4 (CH), 123.9 (CH), 72.8 (C), 54.9 (CH_3). HRMS (ESI) *m/z*: 524.1378 $[M+H]^+$, $C_{30}H_{23}ClN_3O_4^+$ required 524.1372.

Methyl 2-(3,4-bis(benzamido)phenyl)-2-isocyano-2-(4-nitrophenyl)acetate (3ea)

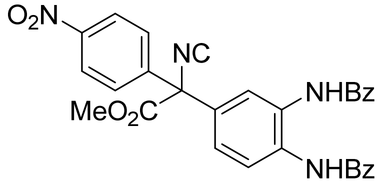

99 mg (74%) of **3ea** were obtained from **1e** (72.6 mg, 0.33 mmol) and **2a** (78.6 mg, 0.25 mmol), after flash chromatography eluting with hexane/EtOAc (7:3).

White solid; m.p. 186.3-187.7 ºC; ^{1}H NMR (300 MHz, DMSO) δ 10.20 (1H, br s), 10.10 (1H, br s), 8.33 (2H, d, *J* = 9.0 Hz), 7.91 (4H, d, *J* = 8.3 Hz), 7.81 (1H, s), 7.71 (3H, d, *J* = 9.0 Hz), 7.61 – 7.43 (6H, m), 7.27 (1H, dd, *J* = 8.6, 2.4 Hz), 3.91 (3H, s); ^{13}C NMR (75 MHz, DMSO) δ 166.3 (C), 165.9 (C), 165.6 (C), 162.6 (C), 148.0 (C), 142.9 (C), 134.0 (C), 133.9 (C), 132.8 (C), 132.4 (C), 132.1 (CH), 131.6 (C), 128.7 (CH), 128.6 (CH), 128.6 (CH), 127.7 (CH), 127.6 (CH), 126.5 (CH), 124.2 (CH), 123.9 (CH), 72.8 (C), 55.1 (CH_3); HRMS (ESI) *m/z*: 535.1583 $[M+H]^+$, $C_{30}H_{23}N_4O_6^+$ required 535.1612.

Methyl 2-(3,4-bis(benzamido)phenyl)-2-isocyano-2-(2-nitrophenyl)acetate (3fa)

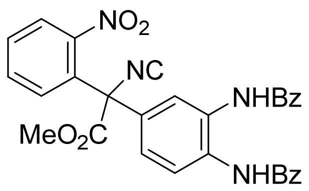

118 mg (88%) of **3fa** were obtained from **1f** (72.5 mg, 0.33 mmol) and **3a** (78.6 mg, 0.25 mmol), after flash chromatography eluting with hexane/EtOAc (6:4).

White solid; m.p. 171.2-171.9 ºC; ^{1}H NMR (400 MHz, DMSO) δ 10.27 (1H, br s), 10.17 (1H, br s), 8.25 – 8.15 (1H, m), 7.98 – 7.86 (6H, m), 7.81 – 7.73 (2H, m), 7.63 – 7.47 (6H, m), 7.38 (1H, dd, *J* = 8.6, 2.5 Hz), 7.02 – 6.95 (1H, m), 3.82 (3H, s); ^{13}C NMR (101 MHz, DMSO) δ 166.0 (C), 165.9 (C), 165.7 (C), 161.7 (C), 147.5 (C), 134.4 (CH), 134.1 (C), 133.9 (C), 132.7 (C), 132.1 (CH), 131.8 (C), 131.6 (CH), 131.5 (C), 131.1 (CH), 130.4 (C), 128.6 (CH), 128.6 (CH), 127.7 (CH), 127.6 (CH), 126.7 (CH), 126.2 (CH), 123.9 (CH), 123.6 (CH), 70.6 (C), 54.4 (CH); HRMS (ESI) *m/z*: 535.1608 [M+H]+, $C_{30}H_{23}N_4O_6^+$ required 535.1612.

Methyl 2-(3,4-bis(benzamido)phenyl)-2-isocyanopropanoate (3ga)

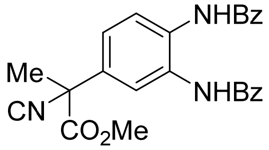

81 mg (76%) of **3ga** were obtained from **1g** (38.0 mg, 0.33 mmol) and **2a** (78.6 mg, 0.25 mmol), after flash chromatography eluting with hexane/EtOAc (6:4).

Yellow solid; m.p. 183-186 ºC; ^{1}H NMR (500 MHz, $CDCl_3$) δ 9.50 (1H, s), 9.49 (1H, s), 8.04-7.96 (4H, m), 7.63-7.50 (9H, m), 7.14 (1H, dd, *J* = 8.5, 2.5 Hz), 3.64 (3H, s), 1.68 (3H, s). ^{13}C NMR (75 MHz, $CDCl_3$) δ 168.0 (C), 166.7 (C), 166.2 (C), 160.4 (C), 134.1 (C), 133.1 (C), 132.9 (C), 132.4 (CH), 132.3 (CH), 131.6 (C), 130.7 (C), 128.8 (CH), 128.7 (CH), 127.5 (CH), 127.4 (CH), 126.3 (CH), 122.8 (CH), 122.0 (CH), 65.5 (C), 53.8 (CH_3), 27.1 (CH_3). HRMS (ESI) *m/z*: 428.1605 [M+H]$^+$, $C_{25}H_{22}N_3O_4^+$ required 428.1605.

Methyl 2-(3,4-bis(benzamido)-5-methylphenyl)-2-isocyano-2-phenylacetate (3ab)

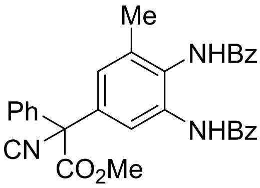

118 mg (94%) of **3ab** were obtained from **1a** (57.8 mg, 0.33 mmol) and **2b** (82.1 mg, 0.25 mmol), after flash chromatography eluting with hexane/EtOAc (6:4).

Yellow solid; m.p. 164-166 ºC; ^{1}H NMR (300 MHz, $CDCl_3$) δ 9.39 (1H, s), 9.28 (1H, br s), 7.91 (2H, d, *J* = 7.0 Hz), 7.81 (2H, d, *J* = 7.0 Hz), 7.50-7.45 (3H, m), 7.44-7.34 (4H, m), 7.31-7.15 (6H, m), 3.70 (3H, s), 2.37 (3H, s). ^{13}C NMR (75 MHz, $CDCl_3$) δ 167.3 (C), 166.7 (C), 166.2 (C), 161.9 (C), 136.8 (C), 136.6 (C), 135.1 (C), 133.4 (C), 133.3 (C), 133.0 (C), 132.21 (CH), 132.16 (CH), 130.6 (C), 129.1 (CH), 128.8 (CH), 128.6 (CH), 128.5 (CH), 127.5 (CH), 127.0 (CH), 126.9 (CH), 121.9 (CH), 73.2 (C), 54.1 (CH_3), 19.1 (CH_3). HRMS (ESI) *m/z*: 504.1923 [M+H]$^+$, $C_{31}H_{26}N_3O_4^+$ required 504.1918.

Methyl 2-(3,4-bis(benzamido)-5-fluorophenyl)-2-isocyano-2-phenylacetate (3ac)

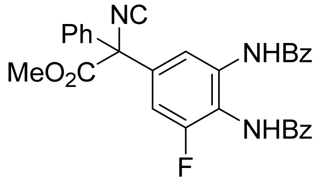

85 mg (67%) of **3ac** were obtained from **1a** (57.8 mg, 0.33 mmol) and **2c** (83.1 mg, 0.25 mmol), after flash chromatography eluting with hexane/EtOAc (7:3).

Orange oil; ^{1}H NMR (300 MHz, $CDCl_3$) δ 9.61 (1H, br s), 8.53 (1H, br s), 8.15 – 8.04 (1H, m), 8.01 – 7.90 (4H, m), 7.81 (1H,

t, J = 1.8 Hz), 7.62 – 7.32 (10H, m), 7.07 (1H, dd, J = 10.8, 2.2 Hz), 3.84 (3H, s); ^{13}C NMR (75 MHz, $CDCl_3$) δ 167.1 (C), 166.1 (C), 162.8 (C), 154.8 (C), 136.3 (C), 136.2 (C), 134.6 (d, J = 364.2 Hz, C), 134.2 (C), 134.1 (C), 133.6 (C), 132.9 (CH), 132.4 (CH), 129.5 (CH), 129.1 (CH), 129.0 (CH), 128.8 (CH), 127.8 (CH), 127.7 (CH), 127.0 (CH), 120.1 (d, J = 3.3 Hz, CH), 119.7 (d, J = 14.3 Hz, C), 111.81 (d, J = 23.7 Hz, CH), 73.2 (C), 54.6 (CH_3). ^{19}F NMR (282 MHz, $CDCl_3$) δ -119.25. HRMS (ESI) *m/z*: 530.1463 $[M+Na]^+$, $C_{30}H_{22}FN_3NaO_4^+$ required 530.1487.

Methyl 2-(3,4-bis(benzamido)-5-chlorophenyl)-2-isocyano-2-phenylacetate (3ad)

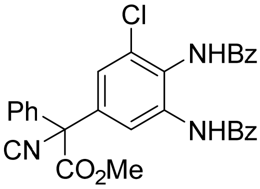

130 mg (99%) of **3ad** were obtained from **1a** (57.8 mg, 0.33 mmol) and **2d** (87.3 mg, 0.25 mmol), after flash chromatography eluting with hexane/EtOAc (6:4).

Yellow solid; m.p. 155-157 ºC; ^{1}H NMR (300 MHz, $CDCl_3$) δ 9.67 (1H, br s), 8.36 (1H, br s), 8.04 (1H, d, J = 2.2 Hz), 7.97 (2H, d, J = 7.0 Hz), 7.92 (2H, d, J = 7.0 Hz), 7.62 (1H, t, J = 7.4 Hz), 7.56-7.48 (3H, m), 7.47-7.40 (8H, m), 3.91 (3H, s). ^{13}C NMR (75 MHz, $CDCl_3$) δ 167.1 (C), 167.0 (C), 165.5 (C), 162.8 (C), 136.4 (C), 136.1 (C), 134.2 (C), 133.7 (C), 133.0 (CH), 132.7 (CH), 132.1 (C), 129.7 (C), 129.5 (CH), 129.1 (CH), 129.0 (CH), 128.7 (CH), 127.6 (CH), 127.4 (CH), 127.0 (CH), 124.7 (CH), 124.0 (CH), 73.0 (C), 54.5 (CH_3). HRMS (ESI) *m/z*: 524.1368 $[M+H]^+$, $C_{30}H_{23}ClN_3O_4^+$ required 524.1372.

Methyl 2-(4,5-bis(benzamido)-2-chlorophenyl)-2-isocyano-2-phenylacetate (3ae)

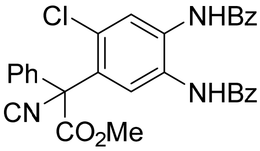

106 mg (81%) of **3ae** were obtained from **1a** (57.8 mg, 0.33 mmol) and **2e** (87 mg, 0.25 mmol), after flash chromatography eluting with hexane/EtOAc (8:2 to 6:4).

Yellow solid; m.p. 160-162 ºC; ^{1}H NMR (300 MHz, $CDCl_3$) δ 9.42 (1H, br s, NH), 8.91 (1H, br s), 7.94 (2H, d, J = 7.0 Hz), 7.90 (1H, s), 7.86 (2H, d, J = 7.0 Hz), 7.60-7.36 (11H, m), 6.88 (1H, s), 3.79 (3H, s). ^{13}C NMR (75 MHz, $CDCl_3$) δ 167.1 (C), 166.8 (C), 166.4 (C), 161.9 (C), 133.4 (C), 133.2 (C), 133.0 (C), 132.8 (C), 132.53 (CH), 132.51 (CH), 132.4 (C), 131.6 (C), 129.7 (CH), 129.1 (CH), 128.93 (CH), 128.88 (CH), 128.5 (C), 127.6 (CH), 126.94 (CH), 126.91 (CH), 72.0 (C), 54.3 (CH_3). HRMS (ESI) *m/z*: 524.1366 $[M+H]^+$, $C_{30}H_{23}ClN_3O_4^+$ required 524.1372.

Methyl 2-(4,5-bis(benzamido)-2-bromophenyl)-2-isocyano-2-phenylacetate (3af)

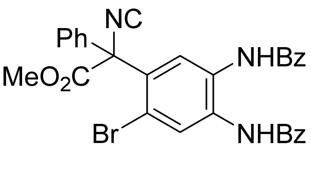

89 mg (63%) of **3af** were obtained from **1a** (57.8 mg, 0.33 mmol) and **2f** (98.2 mg, 0.25 mmol), after flash chromatography eluting with hexane/EtOAc (7:3).

White solid; m.p. 127.0-127.9 ºC; 1H NMR (500 MHz, DMSO) δ 10.11 (1H, br s), 10.06 (1H, br s), 8.20 (1H, s), 7.91 (1H, d, J = 7.0 Hz), 7.84 (1H, d, J = 7.0 Hz), 7.65 – 7.55 (7H, m), 7.54 – 7.45 (4H, m), 7.08 (1H, s), 3.88 (3H, s). ^{13}C NMR (125 MHz, DMSO) δ 166.8 (C), 165.7 (C), 165.6 (C), 161.8 (C), 133.8 (C), 133.7 (C), 133.5 (C), 133.4 (C), 132.7 (C), 132.1 (CH), 132.0 (CH), 131.0 (CH), 130.2 (C), 130.0 (CH), 129.5 (CH), 129.1 (CH), 128.6 (CH), 128.5 (CH), 127.7 (CH), 127.4 (CH), 126.6 (CH), 118.4 (C), 73.5 (C), 54.7 (CH_3); HRMS (ESI) *m/z*: 585.1101 $[M+NH_4]^+$, $C_{30}H_{26}BrN_4O_4^+$ required 585.1132.

Methyl 2-(4,5-bis(benzamido)-2,3-dimethylphenyl)-2-isocyano-2-phenylacetate (3ag)

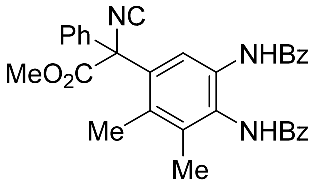

97 mg (75%) of **3ag** were obtained from **1a** (57.8 mg, 0.33 mmol) and **2g** (85.6 mg, 0.25 mmol), after flash chromatography eluting with hexane/EtOAc (7:3).

Colorless oil; ^{1}H NMR (300 MHz, $CDCl_3$) δ 8.77 (br s, 1H, NH), 8.63 (1H, br s), 7.97 – 7.88 (2H, m), 7.81 – 7.72 (2H, m), 7.58 – 7.43 (6H, m), 7.42 – 7.32 (6H, m), 6.98 (1H, s), 3.83 (3H, s), 2.25 (3H, s), 2.14 (3H, s); ^{13}C NMR (75 MHz, $CDCl_3$) δ 168.3 (C), 166.6 (C), 166.1 (C), 161.9 (C), 137.2 (C), 135.7 (C), 135.0 (C), 134.8 (C), 133.8 (C), 133.5 (C), 132.3 (CH), 132.2 (CH), 130.7 (C), 130.5 (C), 129.4 (CH), 129.1 (CH), 129.0 (CH), 128.8 (CH), 127.6 (CH), 127.5 (CH), 127.2 (CH), 123.1 (CH), 73.6 (C), 54.4 (CH_3), 18.1 (CH_3), 16.3 (CH_3); HRMS (ESI) *m/z*: 540.1869 $[M+Na]^+$, $C_{32}H_{27}N_3NaO_4^+$ required 540.1894.

Methyl 2-(4,5-bis(benzamido)-2,3-difluorophenyl)-2-isocyano-2-phenylacetate (3ah)

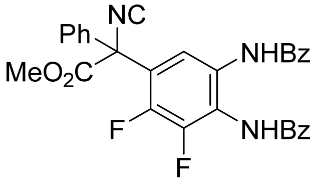

93 mg (71%) of **3ah** were obtained from **1a** (57.8 mg, 0.33 mmol) and **2h** (87.5 mg, 0.25 mmol), after flash chromatography eluting with hexane/EtOAc (7:3).

White solid; m.p. 94.9-96.0 ºC; ^{1}H NMR (500 MHz, CDCl3) δ 9.26 (1H, br s), 8.67 (1H, br s), 7.97 – 7.90 (2H, m), 7.86 – 7.80 (2H, m), 7.63 – 7.56 (1H, m), 7.53 – 7.46 (5H, m), 7.45 – 7.38 (5H, m), 7.13 (1H, d, *J* = 4.9 Hz), 3.85 (3H, s). ^{13}C NMR (126 MHz, $CDCl_3$) δ 166.9 (C), 166.8 (C), 166.1 (C), 162.9 (C), 146.4 (dd, $^{1}J_{C-F}$ = 252.8. $^{2}J_{C-F}$ = 13.2 Hz, C), 145.5 (dd, $^{1}J_{C-F}$ = 250.7, $^{2}J_{C-F}$ = 15.6 Hz, C), 133.3 (C), 133.1 (CH), 132.5 (CH), 132.5 (C), 132.3 (C), 129.9 (CH), 129.3 (CH), 129.2 (CH), 128.9 (CH), 128.7 (C), 127.9 (CH), 127.5 (CH), 126.7 (CH), 126.2 (d, $^{2}J_{C-F}$ = 9.3 Hz, C), 122.3 (d, $^{2}J_{C-F}$ = 11.4 Hz, C), 120.1 (CH), 69.7 (C), 54.7 (CH_3). ^{19}F NMR (282 MHz, $CDCl_3$) δ -136.67 (d, *J* = 20.5 Hz), -141.75 (d, *J* = 19.6 Hz); HRMS (ESI) *m/z*: 548.1370 $[M+Na]^+$, $C_{30}H_{21}F_2N_3NaO_4^+$ required 548.1392.

Methyl 2-(3,4-bis(4-chlorobenzamido)phenyl)-2-isocyano-2-phenylacetate (3ai)

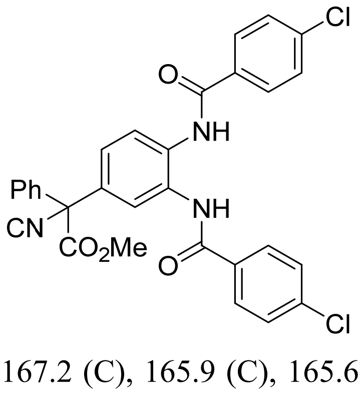

105 mg (75%) of **3ai** were obtained from **1a** (57.8 mg, 0.33 mmol) and **2i** (96.0 mg, 0.25 mmol), after flash chromatography eluting with hexane/EtOAc (5:5).

White solid; m.p. 208-210 ºC; ^{1}H NMR (300 MHz, $CDCl_3$) δ 9.49 (1H, br s), 9.41 (1H, br s), 7.94-7.88 (4H, m), 7.57 (1H, d, *J* = 8.6 Hz), 7.54 (1H, d, *J* = 2.3 Hz), 7.46-7.39 (4H, m), 7.35-7.21 (3H, m), 7.13-7.08 (2H, m), 7.02 (1H, dd, *J* = 8.6, 2.3 Hz), 3.74 (3H, s). ^{13}C NMR (75 MHz, $CDCl_3$) δ 167.2 (C), 165.9 (C), 165.6 (C), 162.1 (C), 138.9 (C), 138.8 (C), 136.5 (C), 134.3 (C), 134.2 (C), 131.7 (C), 131.5 (C), 131.2 (C), 130.5 (C), 129.3 (CH), 129.2 (CH), 129.1 (CH), 128.7 (CH), 126.7 (CH), 126.1 (CH), 125.7 (CH), 124.1 (CH), 73.0 (C), 54.2 (CH_3). HRMS (ESI) *m/z*: 575.1250 $[M+NH_4]^+$, $C_{30}H_{25}Cl_2N_4O_4^+$ required 575.1247.

Methyl 2-(3,4-bis(4-chlorobenzamido)-5-methylphenyl)-2-isocyano-2-phenylacetate (3aj)

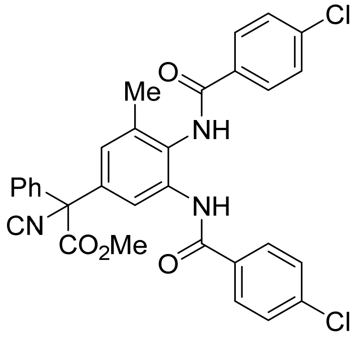

117 mg (82%) of **3aj** were obtained from **1a** (57.8 mg, 0.33 mmol) and **2j** (99.1 mg, 0.25 mmol), after flash chromatography eluting with hexane/EtOAc (5:5).

White solid; m.p. 217-219 ºC; ^{1}H NMR (300 MHz, DMSO-d_6) δ 10.07 (1H, br s), 9.86 (1H, br s), 7.96 (2H, d, *J* = 8.7 Hz), 7.86 (2H, d, *J* = 8.7 Hz), 7.62-7.47 (8H, m), 7.47-7.38 (2H, m), 7.17 (1H, d, *J* = 1.9 Hz), 3.91 (3H, s), 2.27 (3H, s). ^{13}C NMR (75 MHz, DMSO-d_6) δ 167.1 (C), 164.6 (C), 164.2 (C), 161.4 (C), 137.3 (C), 136.61 (C), 136.56 (C), 136.52 (C), 134.9 (C), 134.8 (C), 133.0 (C), 132.8 (C), 130.8 (C), 129.59 (CH), 129.57 (CH), 129.4 (CH), 129.1 (CH), 128.6 (CH), 128.5 (CH), 126.9 (CH), 125.3 (CH), 121.3 (CH), 73.4 (C), 54.7 (CH_3), 18.6 (CH_3). HRMS (ESI) *m/z*: 589.1405 $[M+NH_4]^+$, $C_{31}H_{27}Cl_2N_4O_4^+$ required 589.1404.

Methyl 2-(4,5-bis(4-chlorobenzamido)-2-methylphenyl)-2-isocyano-2-phenylacetate (3ak)

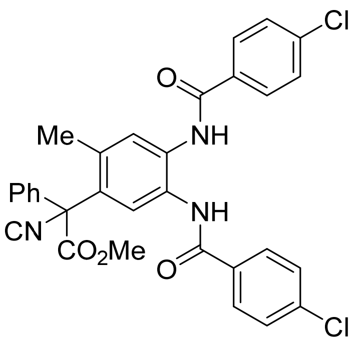

121 mg (85%) of **3ak** were obtained from **1a** (57.8 mg, 0.33 mmol) and **2k** (99.0 mg, 0.25 mmol), after flash chromatography eluting with hexane/EtOAc (5:5).

White solid; m.p. 137-139 ºC; ^{1}H NMR (300 MHz, $CDCl_3$) δ 9.58 (1H, br s), 9.42 (1H, br s), 7.93 (2H, d, *J* = 8.7 Hz), 7.90 (2H, d, *J* = 8.7 Hz), 7.46 (4H, t, *J* = 8.4 Hz), 7.38 (1H, s), 7.36-7.24 (3H, m), 7.21-7.13 (2H, m), 6.98 (1H, s), 3.73 (3H, s), 1.78 (3H, s). ^{13}C NMR (75 MHz, $CDCl_3$) δ 167.5 (C), 165.7 (C), 165.5 (C), 162.0 (C), 138.8 (C), 138.7 (C), 135.6 (C), 134.0 (C), 133.8 (C), 131.4 (C), 131.4 (C), 131.2 (C), 129.23 (CH), 129.18 (CH), 129.15 (CH), 129.1 (CH), 128.7 (CH), 127.7 (C), 126.8 (CH), 72.5 (C), 54.2 (CH_3), 19.6 (CH_3). HRMS (ESI) *m/z*: 589.1406 $[M+NH_4]^+$, $C_{31}H_{27}Cl_2N_4O_4^+$ required 589.1404.

Methyl 2-(3,4-bis(1-naphthamido)phenyl)-2-isocyano-2-phenylacetate (3al)

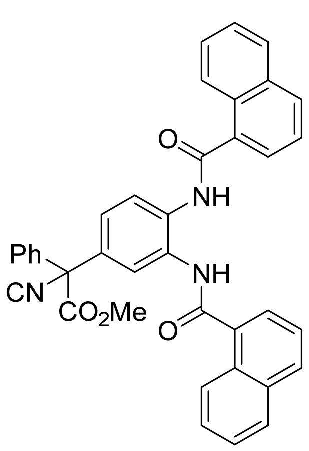

127 mg (86%) of **3al** were obtained from **1a** (57.8 mg, 0.33 mmol) and **2l** (104 mg, 0.25 mmol), after flash chromatography eluting with hexane/EtOAc (6:4).

White solid; m.p. 107-110 ºC; ^{1}H NMR (300 MHz, $CDCl_3$) δ 9.18 (1H, br s), 8.81 (1H, br s), 8.25 (1H, d, *J* = 8.5 Hz), 8.10 (1H, d, *J* = 8.5 Hz), 7.87-7.77 (5H, m), 7.72 (1H, d, *J* = 1.7 Hz), 7.70-7.65 (1H, m), 7.61 (1H, d, *J* = 6.9 Hz), 7.48-7.23 (13H, m), 3.87 (3H, s). ^{13}C NMR (75 MHz, $CDCl_3$) δ 168.6 (C), 168.4 (C), 167.5 (C), 161.9 (C), 136.6 (C), 134.6 (C), 133.62 (C), 133.57 (C), 132.9 (C), 132.5 (C), 131.8 (C), 131.6 (CH), 131.4 (CH), 130.3 (C), 130.0 (C), 129.9 (C), 129.3 (CH), 128.8 (CH), 128.3 (CH), 127.31 (CH), 127.26 (CH), 127.1 CH), 126.5 (CH), 125.7

(CH), 125.6 (CH), 125.6 (CH), 125.0 (CH), 124.9 (CH), 124.6 (CH), 124.5 (CH), 124.3 (CH), 73.4 (C), 54.3 (CH_3). HRMS (ESI) *m/z*: 607.2343 $[M+NH_4]^+$, $C_{38}H_{31}N_4O_4^+$ required 607.2340.

Methyl 2,2-bis(3,4-bis(benzamido)phenyl)-2-isocyanoacetate (3ha)

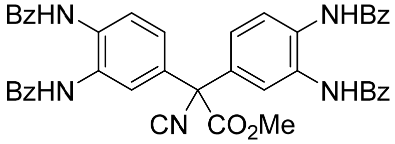

60 mg (33%) of **3ha** were obtained from **1h** (33 mg, 30 µL, 0.33 mmol and **2a** (78.6 mg, 0.25 mmol), after flash chromatography eluting with hexane/EtOAc (6:4).

White solid; m.p. 180-181 ºC; ^{1}H NMR (500 MHz, $CDCl_3$) δ 9.72 (2H, br s), 9.63 (2H, s), 8.03 – 7.95 (4H, m), 7.93 – 7.88 (4H, m), 7.75 (2H, d, *J* = 2.4 Hz), 7.58 (2H, d, *J* = 8.7 Hz), 7.56 – 7.52 (2H, m), 7.52 – 7.48 (2H, m), 7.48 – 7.43 (4H, m), 7.41 – 7.36 (4H, m), 7.15 (2H, dd, *J* = 8.6, 2.4 Hz), 3.78 (3H, s); ^{13}C NMR (125 MHz, $CDCl_3$) δ 167.3 (C), 167.1 (C), 166.7 (C), 162.4 (C), 133.8 (C), 133.8 (C), 133.5 (C), 132.5 (CH), 132.4 (CH), 132.1 (C), 130.5 (C), 128.9 (CH), 128.9 (CH), 127.8 (CH), 127.8 (CH), 125.6 (CH), 125.4 (CH), 125.1 (CH), 72.7 (C), 54.6 (CH_3). HRMS (ESI) *m/z*: 728.2512 $[M+H]^+$, $C_{44}H_{34}N_5O_6^+$ required 728.2504.

Methyl 6,7-bis(benzamido)-4-phenyl-4H-benzo[e][1,3]oxazine-4-carboxylate (3an)

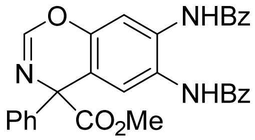

125 mg (96%) of **3an** were obtained from **1a** (57.8 mg, 0.33 mmol) and **2n** (83.0 mg, 0.25 mmol), after flash chromatography eluting with hexane/EtOAc (5:5).

Pink solid; m.p. 180-182 ºC; ^{1}H NMR (300 MHz, $CDCl_3$) δ 9.46 (1H, br s), 8.57 (1H, br s), 7.94 (2H, d, *J* = 7.0 Hz), 7.89 (2H, d, *J* = 7.0 Hz), 7.59 (1H, s), 7.55-7.42 (6H, m), 7.34 (1H, s), 7.33 (1H, s), 7.30-7.19 (5H, m), 3.79 (3H, s). ^{13}C NMR (75 MHz, $CDCl_3$) δ 171.6 (C), 167.0 (C), 166.0 (C), 145.6 (C), 144.5 (C), 142.8 (C), 133.7 (C), 133.2 (C), 133.1 (C), 132.5 (CH), 132.2 (CH), 128.8 (CH), 128.7 (CH), 128.5 (CH), 127.9 (CH), 127.5 (CH), 127.0 (CH), 126.7 (C), 125.6 (CH), 117.3 (C), 112.7 (CH), 65.4 (C), 53.4 (CH_3). HRMS (ESI) *m/z*: 541.2083 $[M+H_2O+NH_4]^+$, $C_{30}H_{29}N_4O_6^+$ required 541.2082.

Methyl 6,7-bis(benzamido)-4-(2-nitrophenyl)-4H-benzo[e][1,3]oxazine-4-carboxylate (3fn)

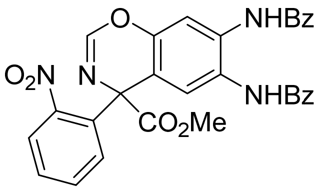

113 mg (80%) of **3fn** were obtained from **1f** (73.0 mg, 0.33 mmol) and **2n** (83.1 mg, 0.25 mmol), after flash chromatography eluting with hexane/EtOAc (4:6).

White solid; m.p. 260-262 ºC; ^{1}H NMR (300 MHz, DMSO-d_6) δ 10.19 (1H, br s), 10.10 (1H, br s), 8.11-7.84 (5H, m), 7.81-7.48 (11H, m), 7.32 (1H, d, *J* = 7.7 Hz), 3.73 (3H, s). ^{13}C NMR (75 MHz, DMSO-d_6) δ 169.4 (C), 165.9 (C), 165.6 (C), 148.2 (C), 146.6 (C), 144.2 (C), 135.3 (C), 134.0 (C), 133.9 (C), 133.6 (C), 133.0 (CH), 132.1 (CH), 132.0 (CH), 129.9 (CH), 129.7 (CH), 128.7 (CH), 128.5 (CH), 127.9 (C), 127.7 (CH), 127.6 (CH), 125.5 (CH), 125.3 (CH), 114.6 (C), 63.8 (C), 53.0 (CH_3). HRMS (ESI) *m/z*: 586.1930 $[M+H_2O+NH_4]^+$, $C_{30}H_{28}N_5O_8^+$ required 586.1932.

Synthesis of compound 3aa at 2.5 mmol-scale

α-Phenyl isocyanoacetate **1a** (578 mg, 3.3 mmol, 1.3 equiv) was added to a solution of *o*-benzoquinone diimide **2a** (786 mg, 2.5 mmol, 1 equiv) and silver oxide (29 mg, 0.125 mmol, 0.05 equiv) in CH_2Cl_2 (30 mL). The mixture was stirred at rt for 1.5 hours. Then, the reaction mixture was purified by flash column chromatography eluting with hexane/EtOAc (6:4) to give compound **3aa** (1.10 g, 90%) with identical spectroscopic features as those reported for **3aa** in the 0.25 mmol-scale reaction.

Synthesis and characterization data for compounds 4

To a solution of compounds **3** (0.2 mmol, 1 equiv) in MeOH (2 mL, 0.1 M) was added 6 M HCl (aq) (10 drops). The reaction was stirred for 4 h and quenched with $NaHCO_3$(aq) sat. until pH 9. The methanol was removed under reduced pressure and the aqueous mixture was extracted with CH_2Cl_2 (3 × 30 mL). The combined organic phases were washed with brine (40 mL), dried over Na_2SO_4, and concentrated under reduced pressure. The mixture was chromatographed using hexane/EtOAc mixtures as eluent.

Methyl 2-amino-2-(3,4-bis(benzamido)phenyl)-2-phenylacetate (4aa)

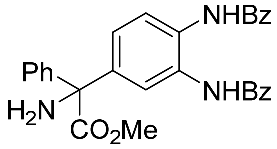

95 mg (99%) of **4aa** were obtained from **3aa** (98 mg), after flash chromatography eluting with hexane/EtOAc (5:5).

Brown solid; m.p. 191-194 ºC; ^{1}H NMR (300 MHz, $CDCl_3$) δ 9.45 (1H, br s, NH), 9.13 (1H, br s), 8.04-7.93 (4H, m), 7.61 (1H, d, *J* = 8.6 Hz), 7.57 (1H, d, *J* = 2.3 Hz), 7.55-7.39 (6H, m), 7.22 (1H, d, *J* = 2.3 Hz), 7.21-7.10 (4H, m), 3.67 (3H, s), 1.87 (2H, br s). ^{13}C NMR (75 MHz, $CDCl_3$) δ 174.6 (C), 166.7 (C), 166.2 (C), 143.8 (C), 141.2 (C), 133.6 (C), 133.3 (C), 132.3 (CH), 132.1 (CH), 130.5 (C), 129.9 (C), 128.8 (CH), 128.7 (CH), 128.2 (CH), 127.6 (CH), 127.6 (CH), 127.5 (CH), 127.2 (CH), 126.6 (CH), 125.5 (CH), 124.4 (CH), 67.9 (C), 52.9 (CH_3). HRMS (ESI) *m/z*: 480.1919 $[M+H]^+$, $C_{29}H_{26}N_3O_4^+$ required 480.1918.

***tert*-Butyl 2-amino-2-(3,4-bis(benzamido)phenyl)-2-phenylacetate (4ba)**

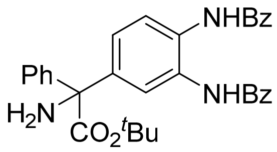

90 mg (86%) of **4ba** were obtained from **3ba** (103 mg), after flash chromatography eluting with hexane/EtOAc (5:5).

Brown solid; m.p. 140-142 ºC; ^{1}H NMR (300 MHz, $CDCl_3$) δ 9.49 (1H, br s), 9.12 (1H, br s), 7.99 (4H, d, *J* = 8.3 Hz), 7.66-7.57 (2H, m), 7.55-7.36 (6H, m), 7.21-7.13 (5H, m), 1.93 (2H, br s), 1.37 (9H, s). ^{13}C NMR (75 MHz, $CDCl_3$) δ 173.0 (C), 166.6 (C), 166.2 (C), 144.3 (C), 141.8 (C), 133.7 (C), 133.4 (C), 132.2 (CH), 132.1 (CH), 130.3 (C), 129.8 (C), 128.8 (CH), 128.7 (CH), 128.0 (CH), 127.6 (CH), 127.5 (CH), 127.3 (CH), 127.2 (CH), 126.7 (CH), 125.1 (CH), 124.5 (CH), 82.6 (C), 68.0 (C), 27.7 (CH_3). HRMS (ESI) *m/z*: 522.2390 $[M+H]^+$, $C_{32}H_{32}N_3O_4^+$ required 522.2387.

Methyl 2-amino-2-(3,4-bis(benzamido)-5-methylphenyl)-2-phenylacetate (4ab)

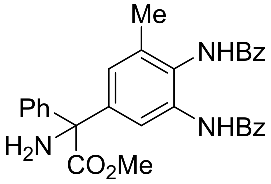

98 mg (99%) of **4ab** were obtained from **3ab** (101 mg), after flash chromatography eluting with hexane/EtOAc (5:5).

Yellow solid; m.p. 172-174 ºC; ^{1}H NMR (300 MHz, $CDCl_3$) δ 9.40 (1H, br s), 8.97 (1H, br s), 7.91 (2H, d, *J* = 7.0 Hz), 7.83 (2H, d, *J* = 7.1 Hz), 7.55-7.31 (8H, m), 7.23-6.99 (6H, m), 3.54 (3H, s), 2.36 (3H, s), 1.76 (2H, br s). ^{13}C NMR (75 MHz, $CDCl_3$) δ 174.3 (C), 166.8 (C), 166.0 (C), 143.7 (C), 142.3 (C), 136.0 (C), 133.5 (C), 133.2 (C), 132.9 (C), 132.14 (CH), 132.07 (CH), 129.3 (C), 128.8 (CH), 128.5 (CH), 128.2 (CH), 128.0 (CH), 127.7 (CH), 127.45 (CH), 127.42 (CH), 127.35 (CH), 121.8 (CH), 67.8 (C), 52.7 (CH_3), 19.0 (CH_3). HRMS (ESI) *m/z*: 494.2073 $[M+H]^+$, $C_{30}H_{28}N_3O_4^+$ required 494.2074.

Methyl 2-amino-2-(4,5-bis(benzamido)-2-chlorophenyl)-2-phenylacetate (4ae)

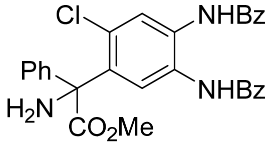

102 mg (99%) of **4ae** were obtained from **3ae** (105 mg), after flash chromatography eluting with hexane/EtOAc (4:6).

Brown solid; m.p. 155-157 ºC; ^{1}H NMR (300 MHz, $CDCl_3$) δ 9.56 (1H, br s, 8.89 (1H, br s), 7.96 (2H, d, *J* = 6.9 Hz), 7.90 (2H, d, *J* = 7.1 Hz), 7.81 (1H, s), 7.60-7.40 (8H, m), 7.30-7.27 (2H, m), 6.96 (1H, s), 3.63 (3H, s), 2.18 (2H, br s). ^{13}C NMR (75 MHz, $CDCl_3$) δ 174.9 (C), 166.7 (C), 166.1 (C), 139.7 (C), 139.6 (C), 133.3 (C), 133.0 (C), 132.4 (CH), 132.3 (CH), 131.7 (C), 130.8 (C), 128.8 (CH), 128.4 (C), 128.3 (CH), 128.1 (CH), 127.6 (CH), 127.5 (CH), 127.4 (CH), 126.4 (CH), 67.0 (C), 52.9 (CH_3). HRMS (ESI) *m/z*: 514.1532 $[M+H]^+$, $C_{29}H_{25}ClN_3O_4^+$ required 514.1528.

References

1. Wang, F.; Xu, P.; Liu, B.-B.; Wang, S.-Y.; Ji, S.-J. *Org. Chem. Front.* **2019**, *6*, 3754–3758.

2. Huang, R.; Chen, X.; Mou, C.; Luo, G.; Li, Y.; Li, X.; Xue, W.; Jin, Z.; Chi, Y. R. *Org. Lett.* **2019**, *21*, 4340−4344.

3. Li, J.-L.; Han, B.; Jiang, K.; Du, W.; Chen, Y.-C. *Bioorg. Med. Chem. Lett.* **2009**, *19*, 3952–3954.

4. Rombouts, J. A.; Ravensbergen, J.; Frese, R. N. ; Kennis, J. T. M.; Ehlers, A. W.; Slootweg, J. C.; Ruijter, E.; Lammertsma, K. Orru, R. V. A. *Chem. Eur. J.* **2014**, *20*, 10285–10291.

***N,N'*-((Cyclohexa-3,5-diene-1,2-diylidene)dibenzamide (2a)**

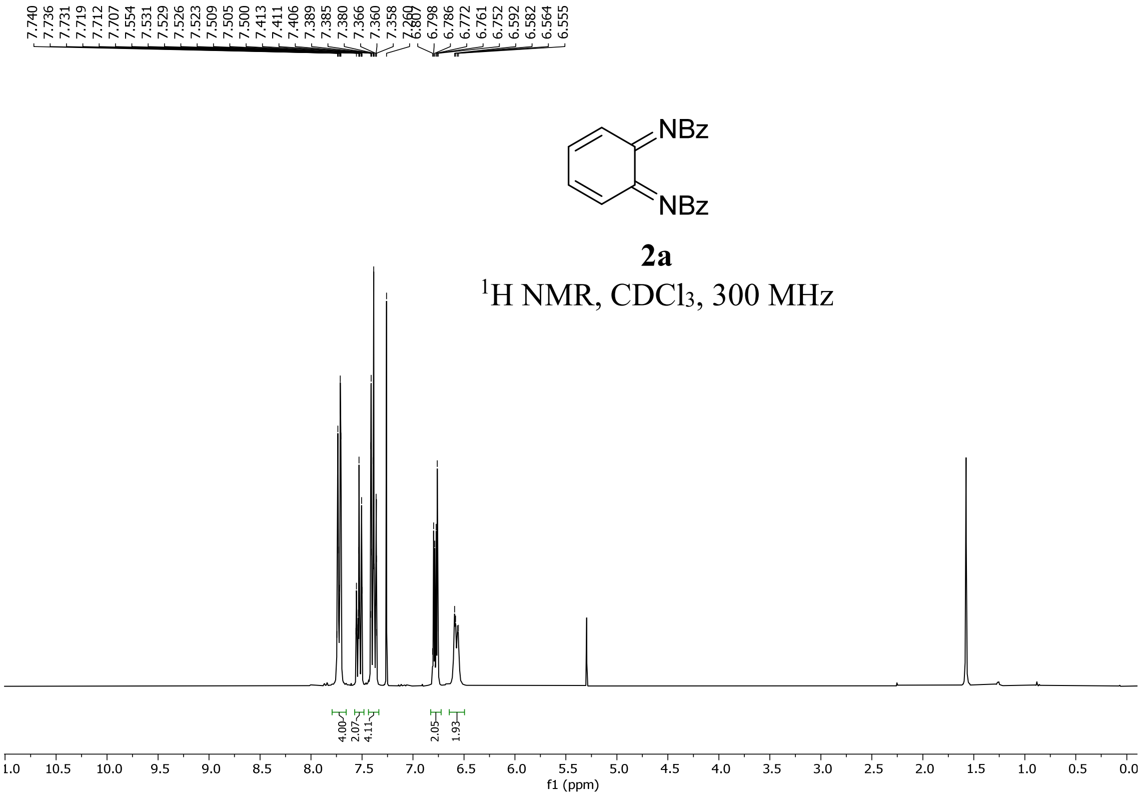

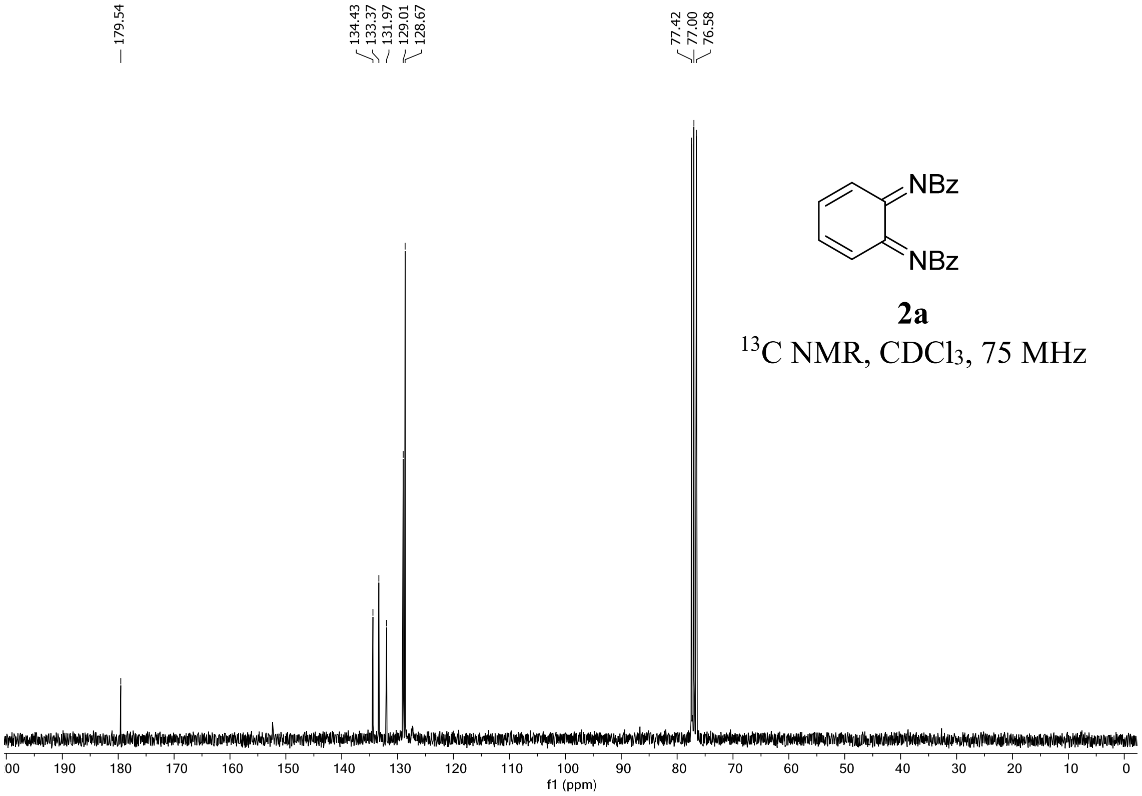

***N,N'*-(3-Methylcyclohexa-3,5-diene-1,2-diylidene)dibenzamide (2b)**

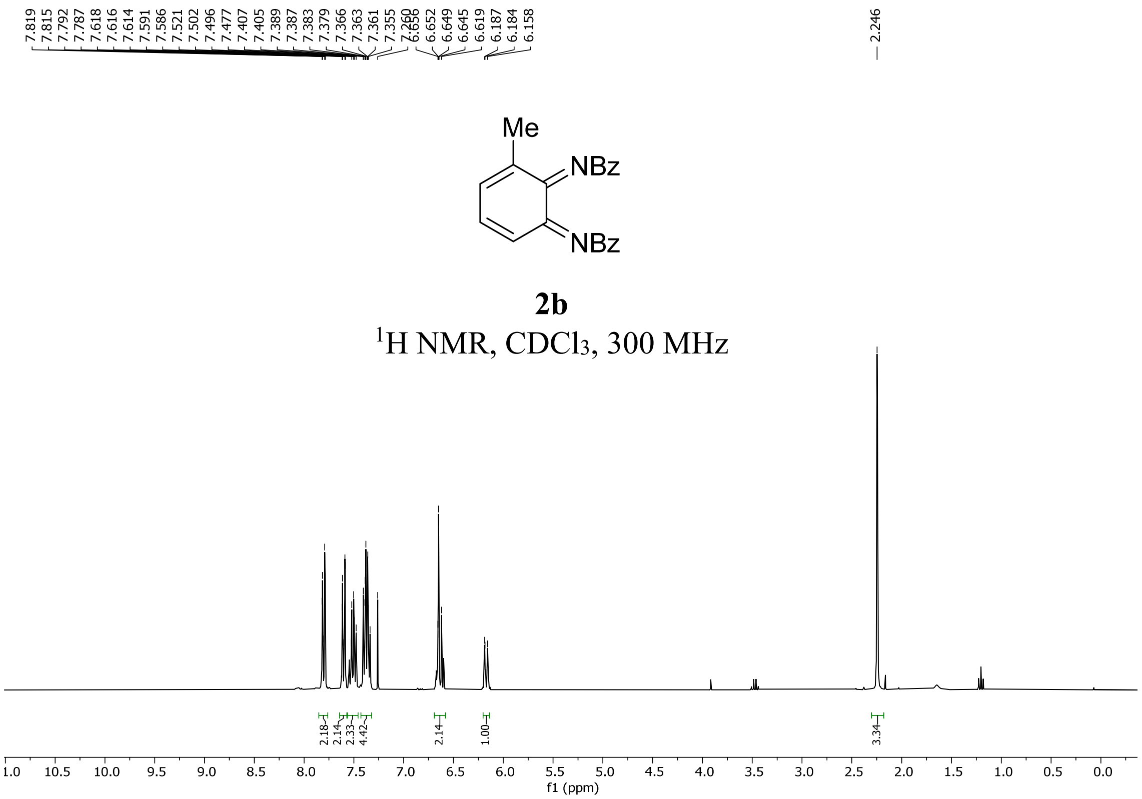

2b
^{1}H NMR, $CDCl_3$, 300 MHz

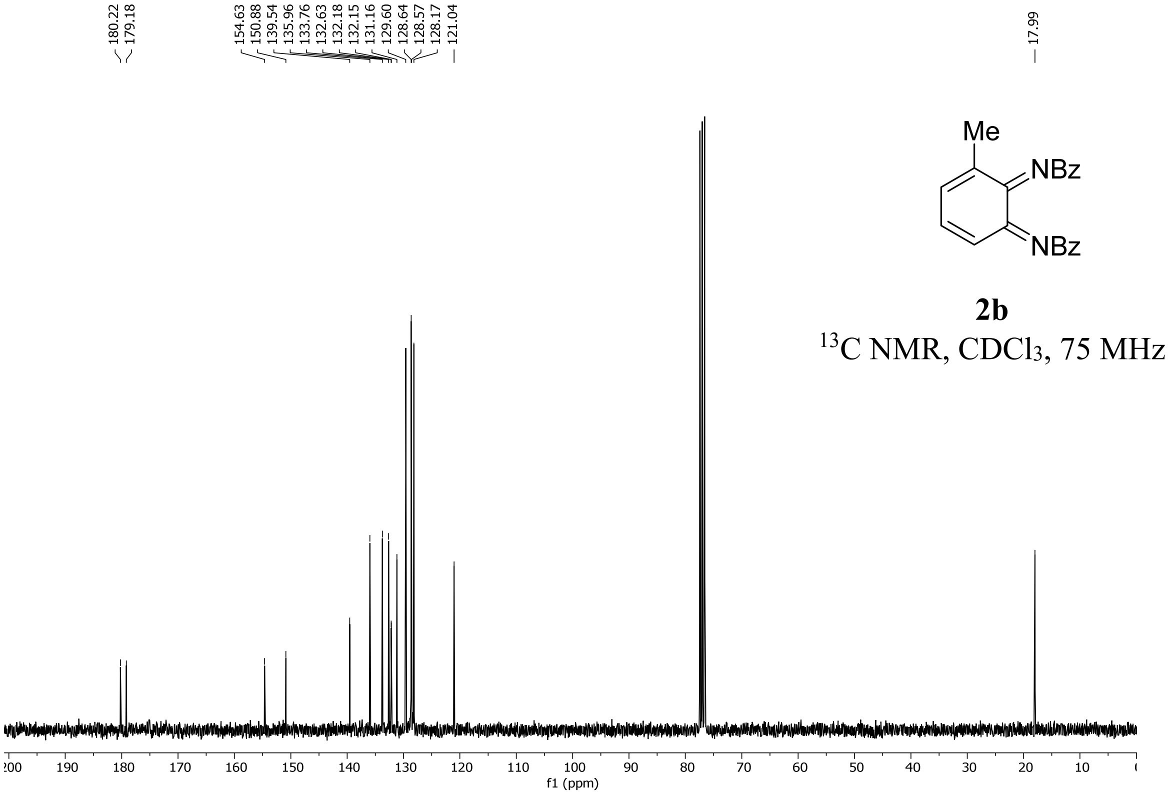

2b
^{13}C NMR, $CDCl_3$, 75 MHz

***N,N'*-(3-Fluorocyclohexa-3,5-diene-1,2-diylidene)dibenzamide (2c)**

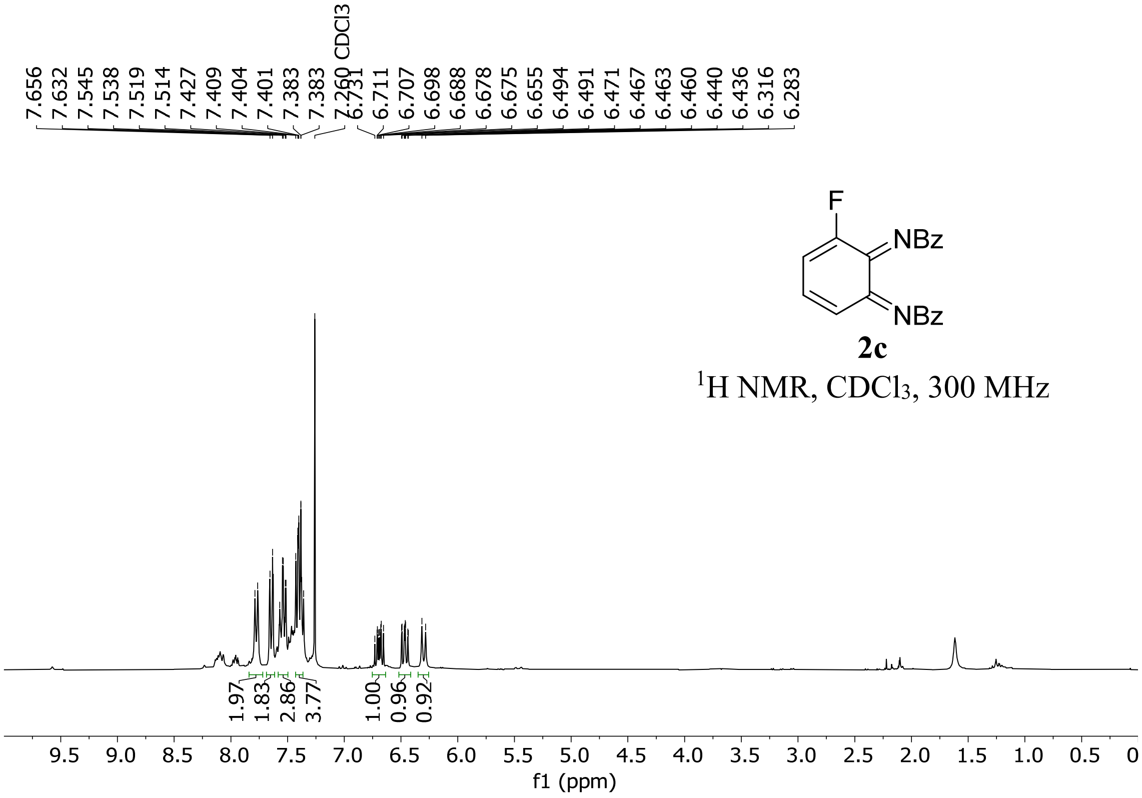

2c

^{1}H NMR, $CDCl_3$, 300 MHz

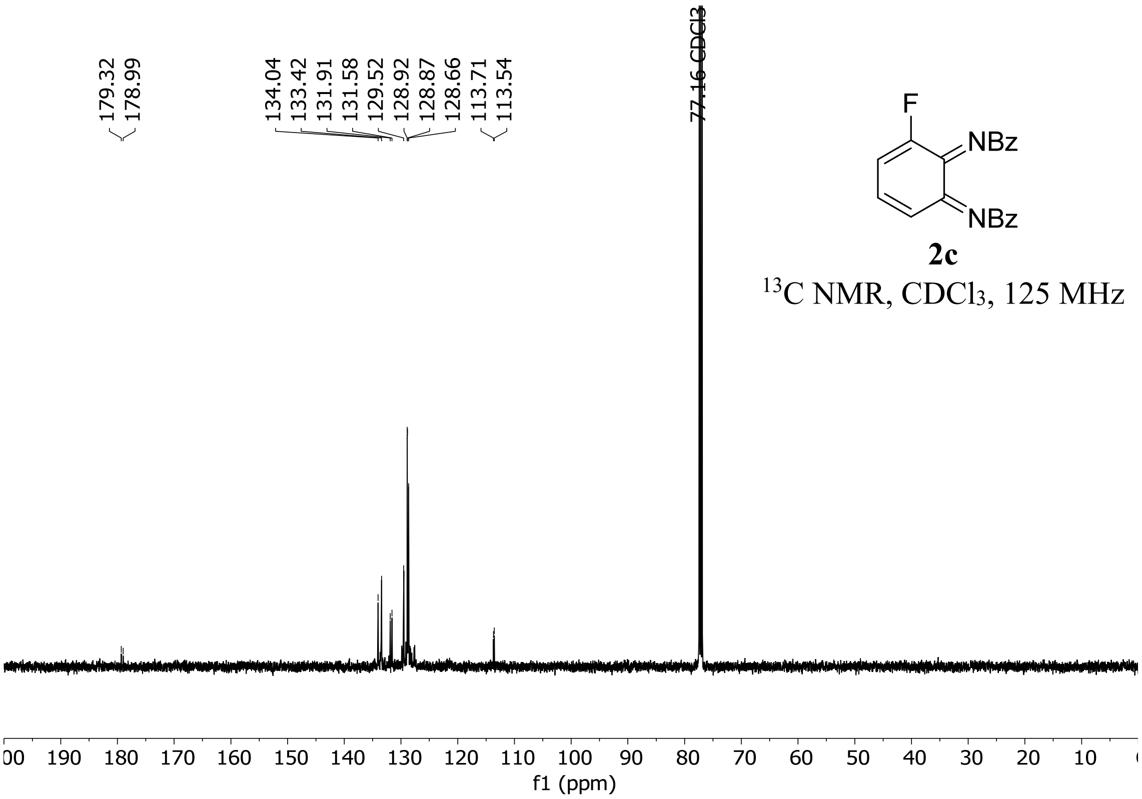

2c

^{13}C NMR, $CDCl_3$, 125 MHz

***N,N'*-(3-Chlorocyclohexa-3,5-diene-1,2-diylidene)dibenzamide (2d)**

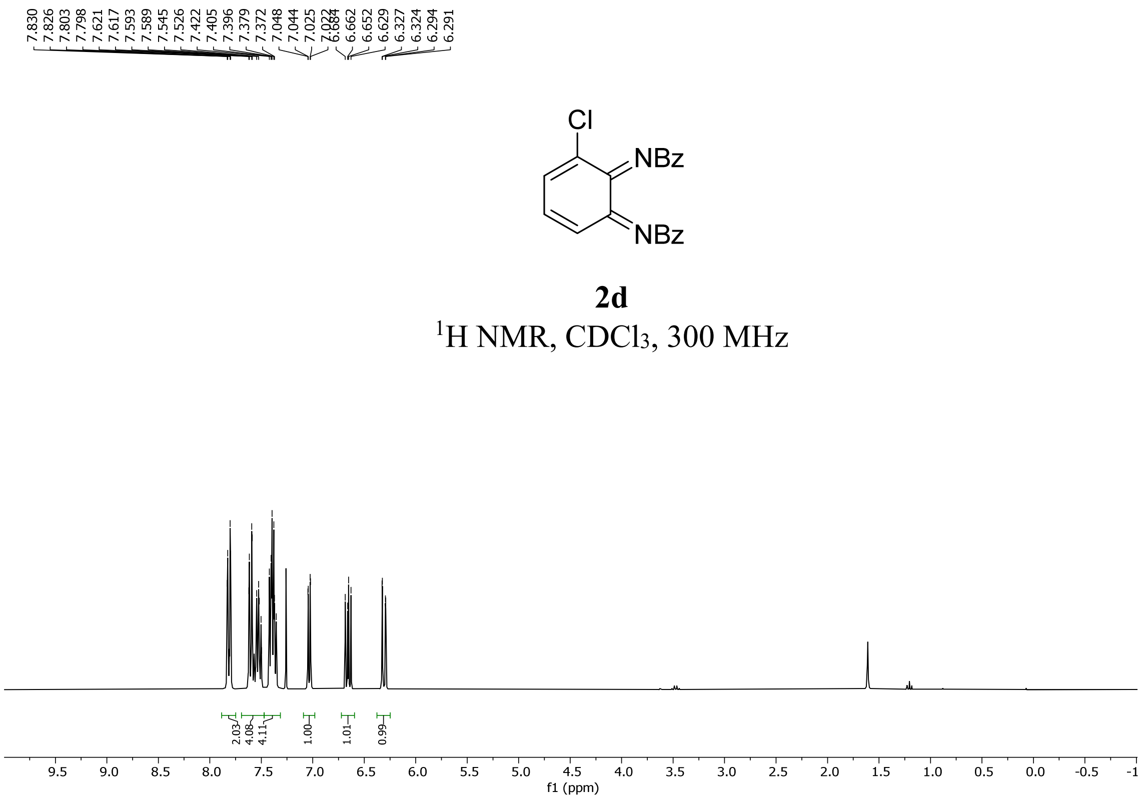

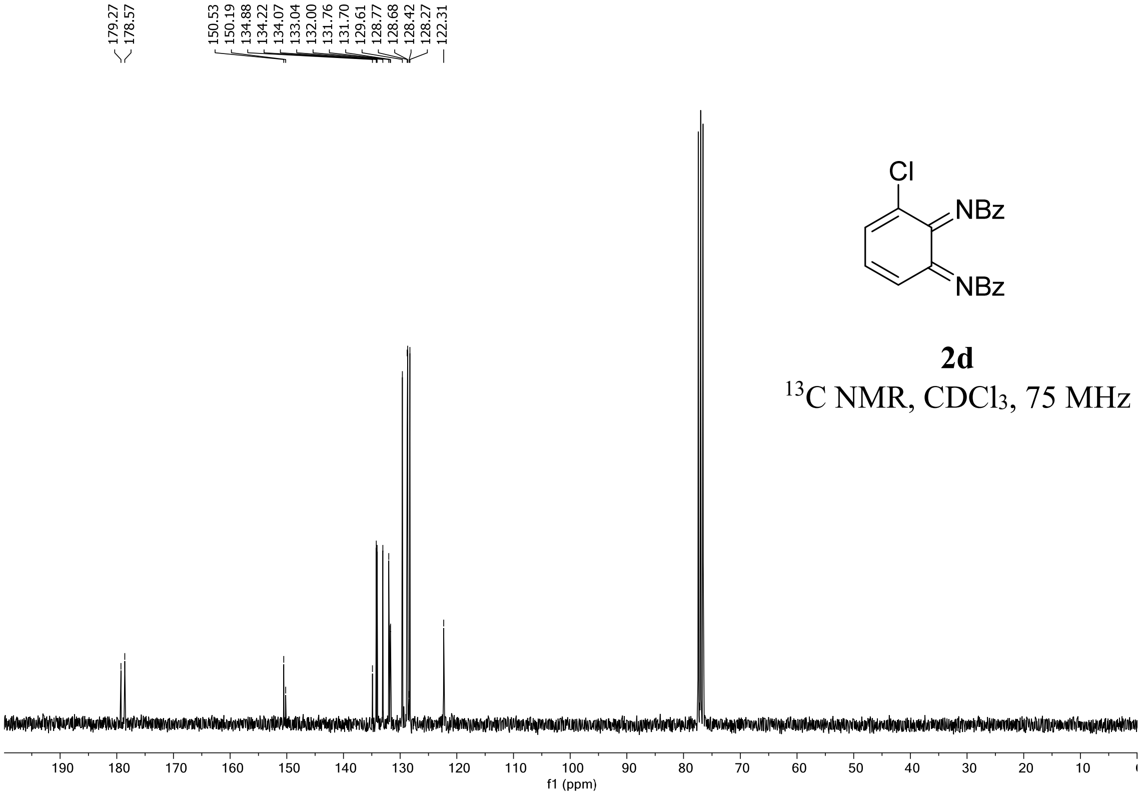

***N,N'*-(4-Chlorocyclohexa-3,5-diene-1,2-diylidene)dibenzamide (2e)**

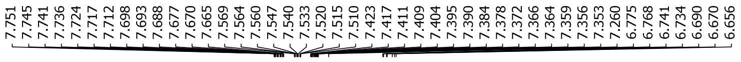

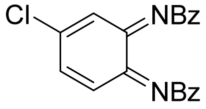

2e

^{1}H NMR, $CDCl_3$, 300 MHz

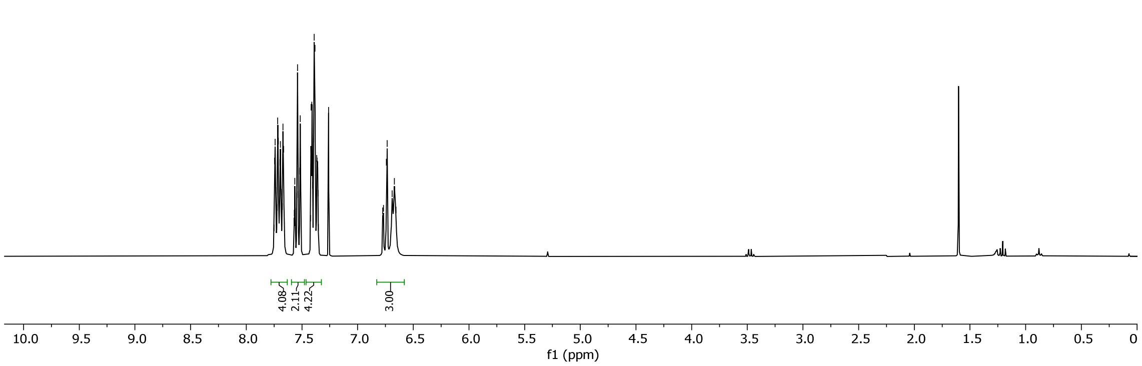

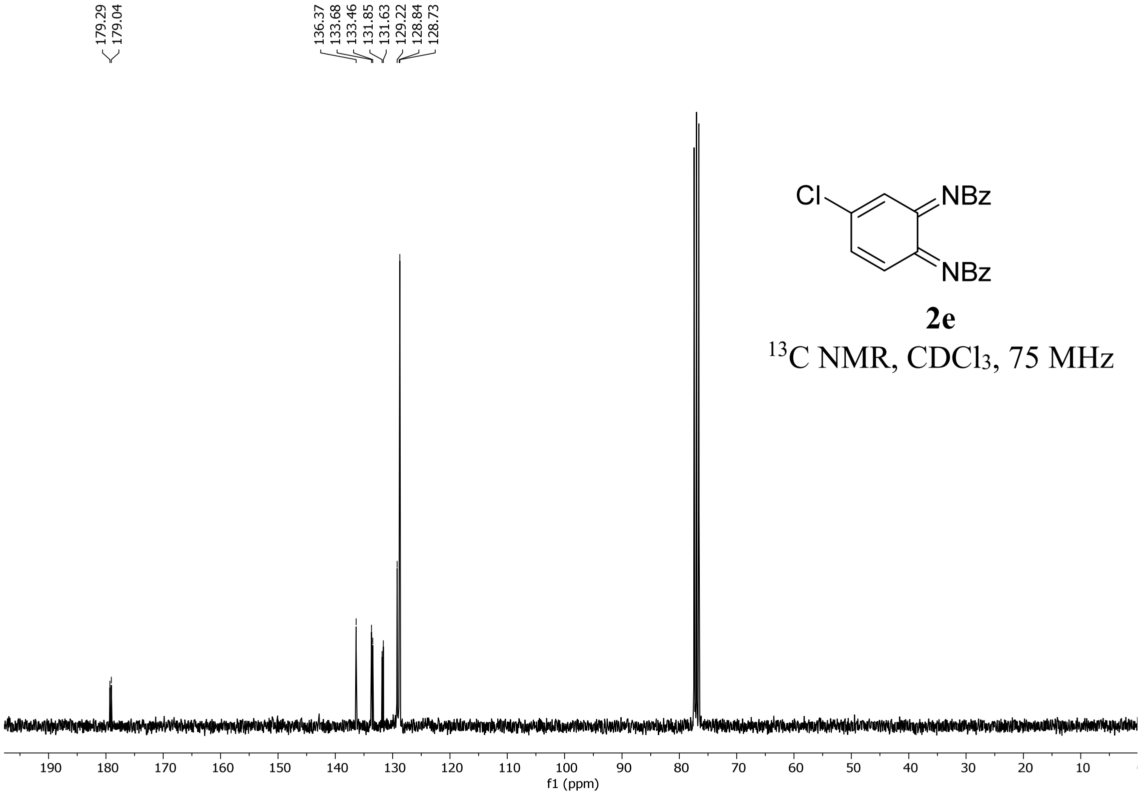

2e

^{13}C NMR, $CDCl_3$, 75 MHz

***N,N'*-(4-Bromocyclohexa-3,5-diene-1,2-diylidene)dibenzamide (2f)**

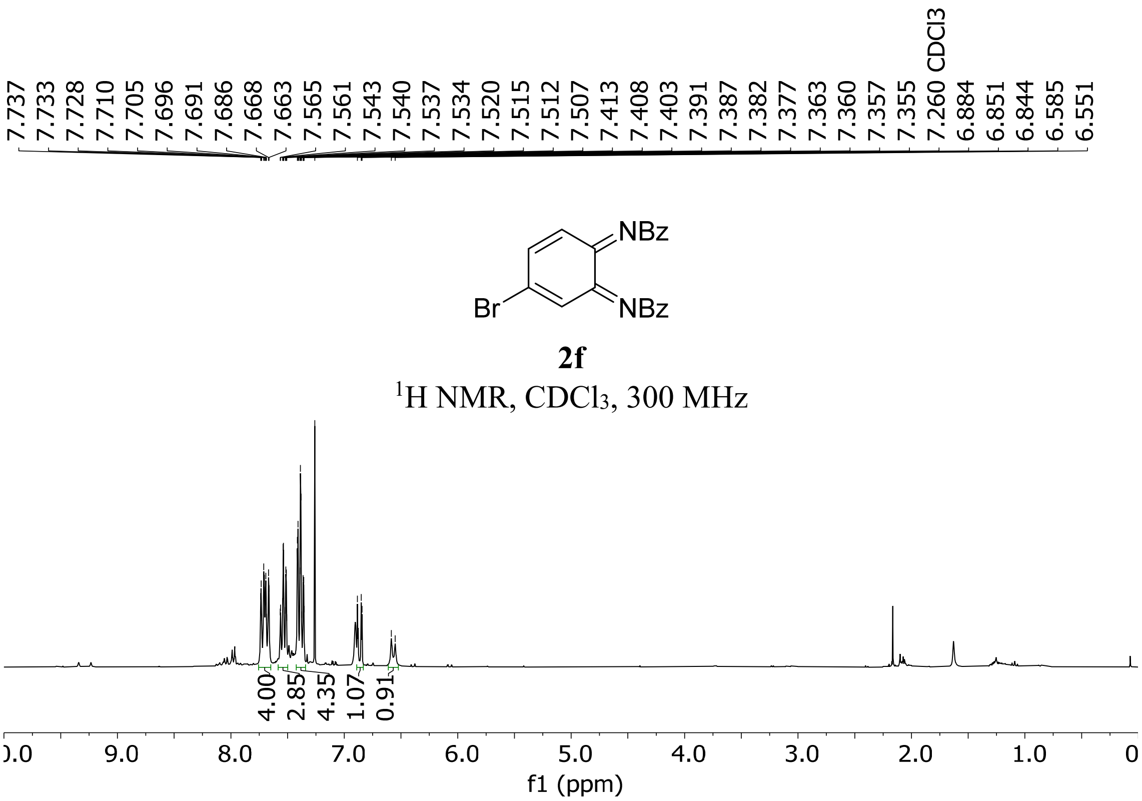

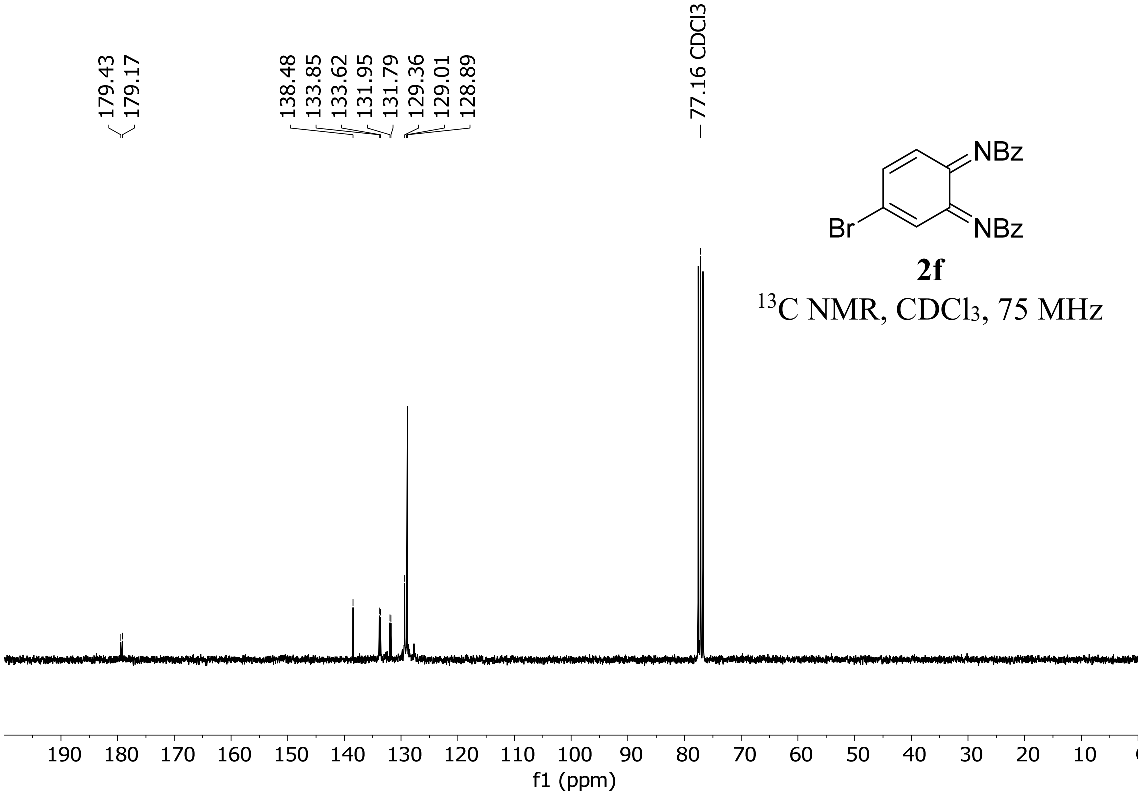

***N,N'*-(3,4-Dimethylcyclohexa-3,5-diene-1,2-diylidene)dibenzamide (2g)**

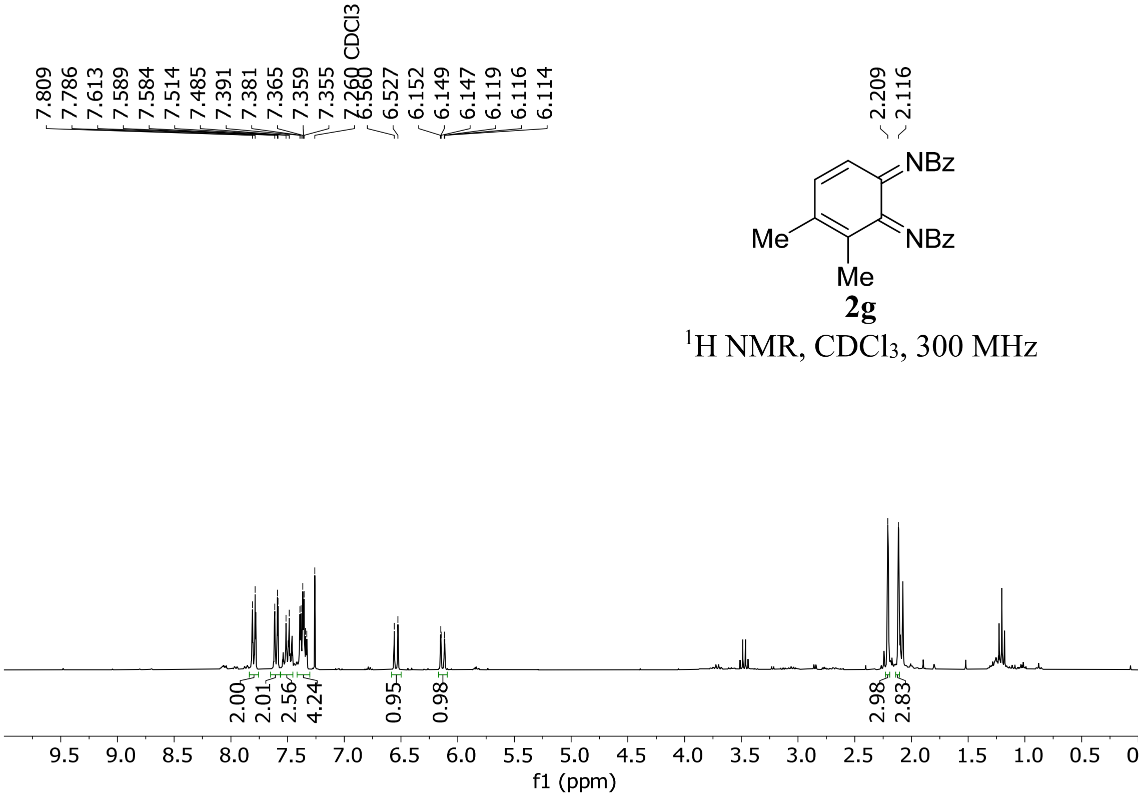

***N,N'*-(3,4-Difluorocyclohexa-3,5-diene-1,2-diylidene)dibenzamide (2h)**

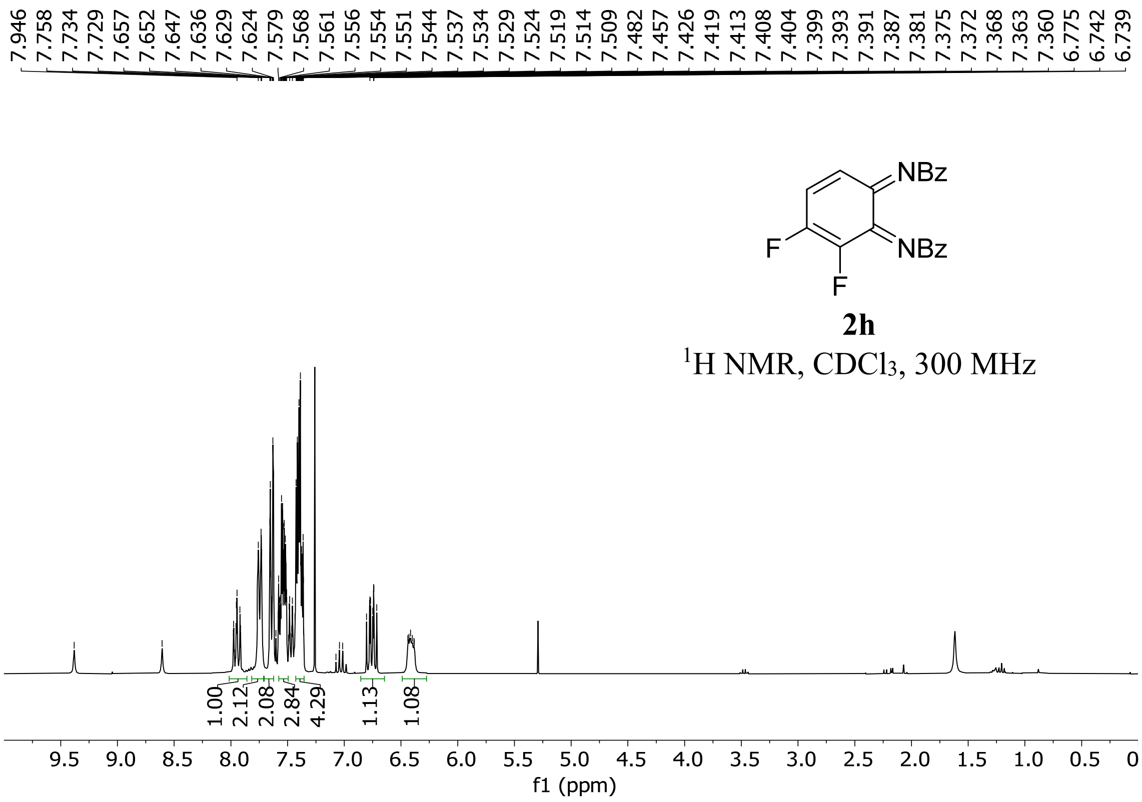

2h

^{1}H NMR, $CDCl_3$, 300 MHz

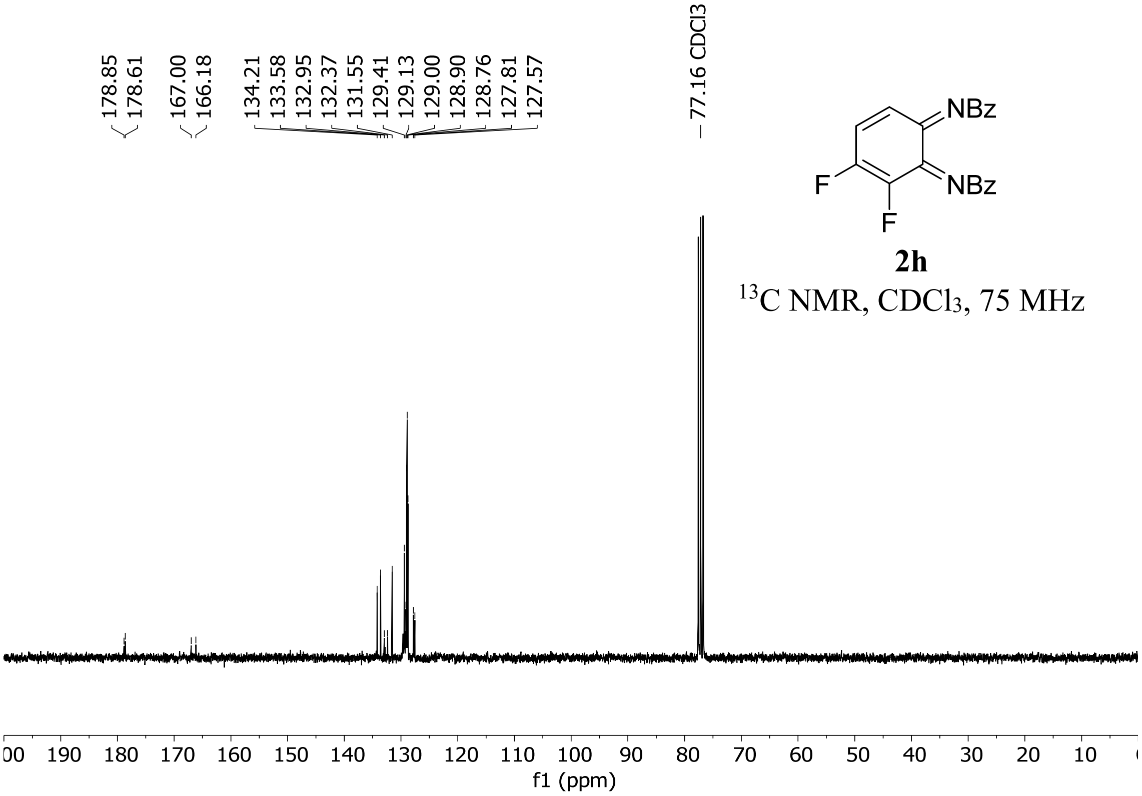

2h

^{13}C NMR, $CDCl_3$, 75 MHz

N,N'-(Cyclohexa-3,5-diene-1,2-diylidene)bis(4-chlorobenzamide) (2i)

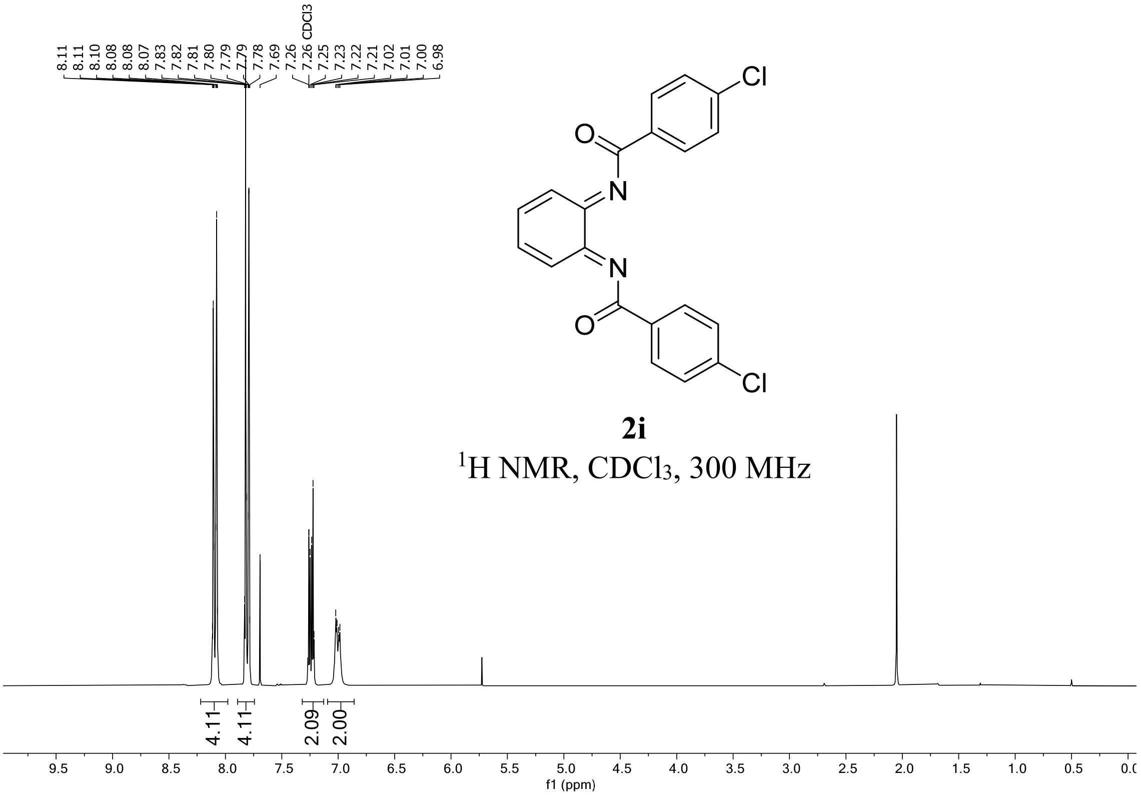

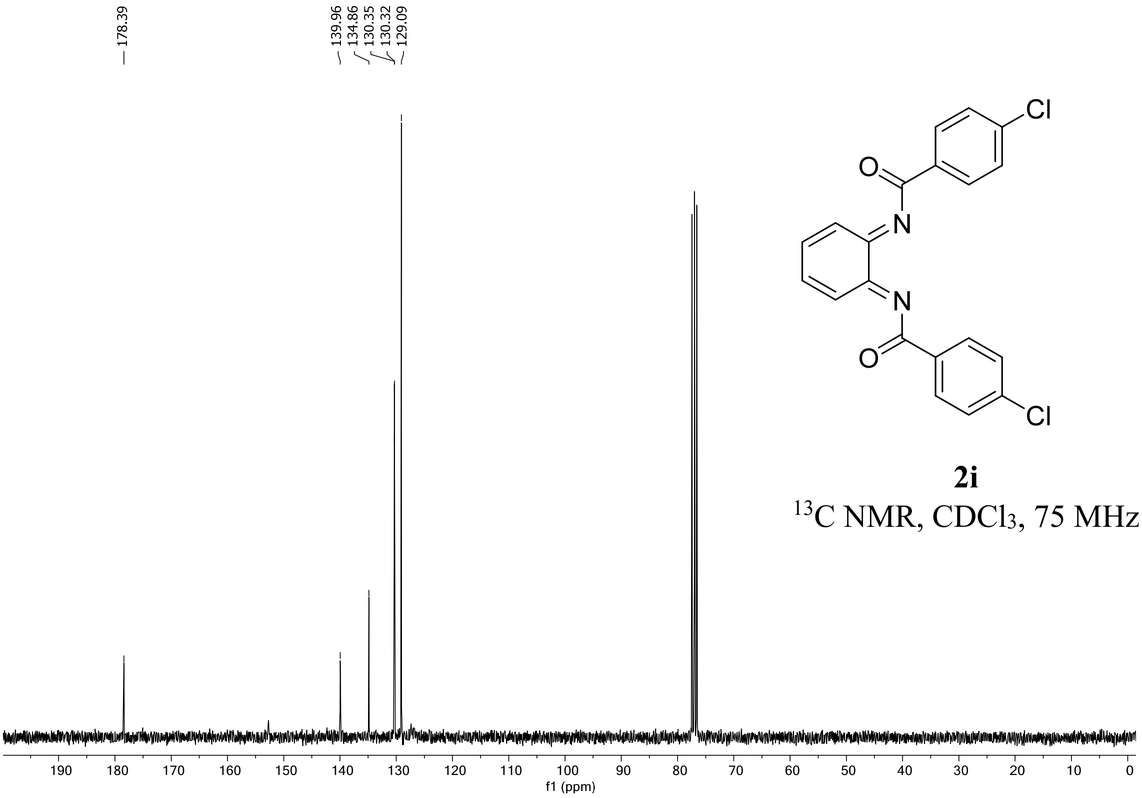

N,N'-(3-methylcyclohexa-3,5-diene-1,2-diylidene)bis(4-chlorobenzamide) (2j)

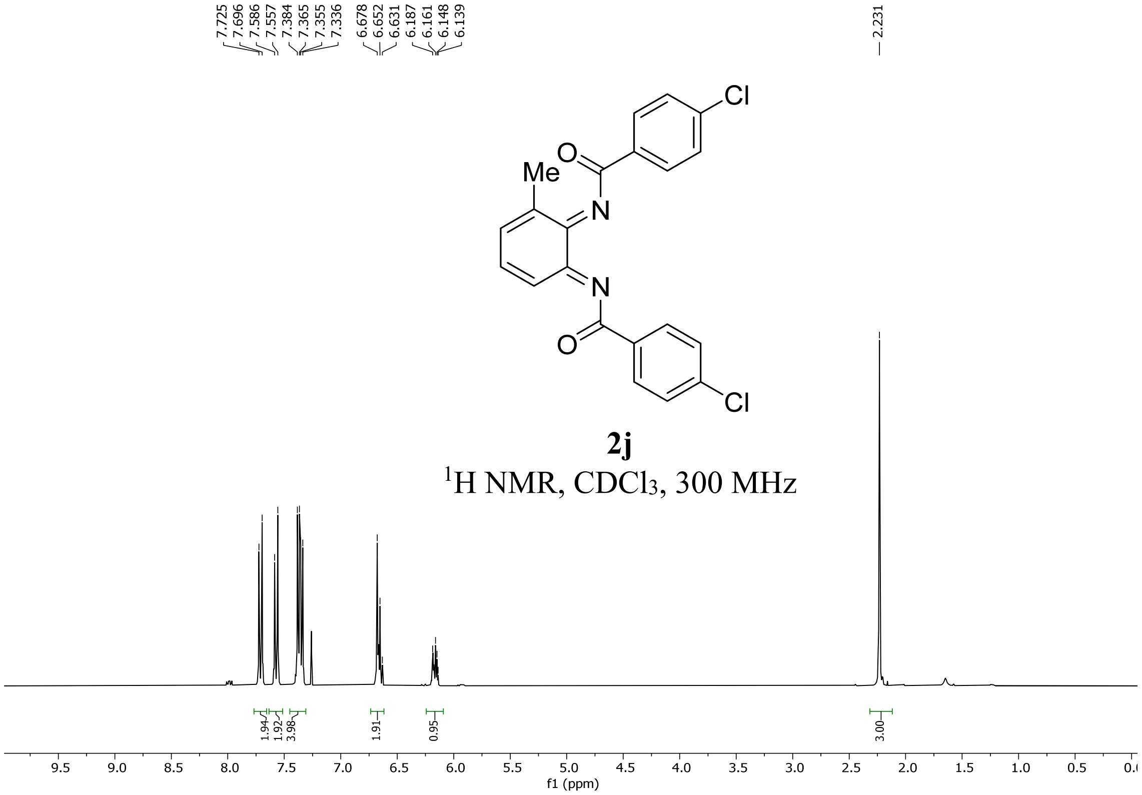

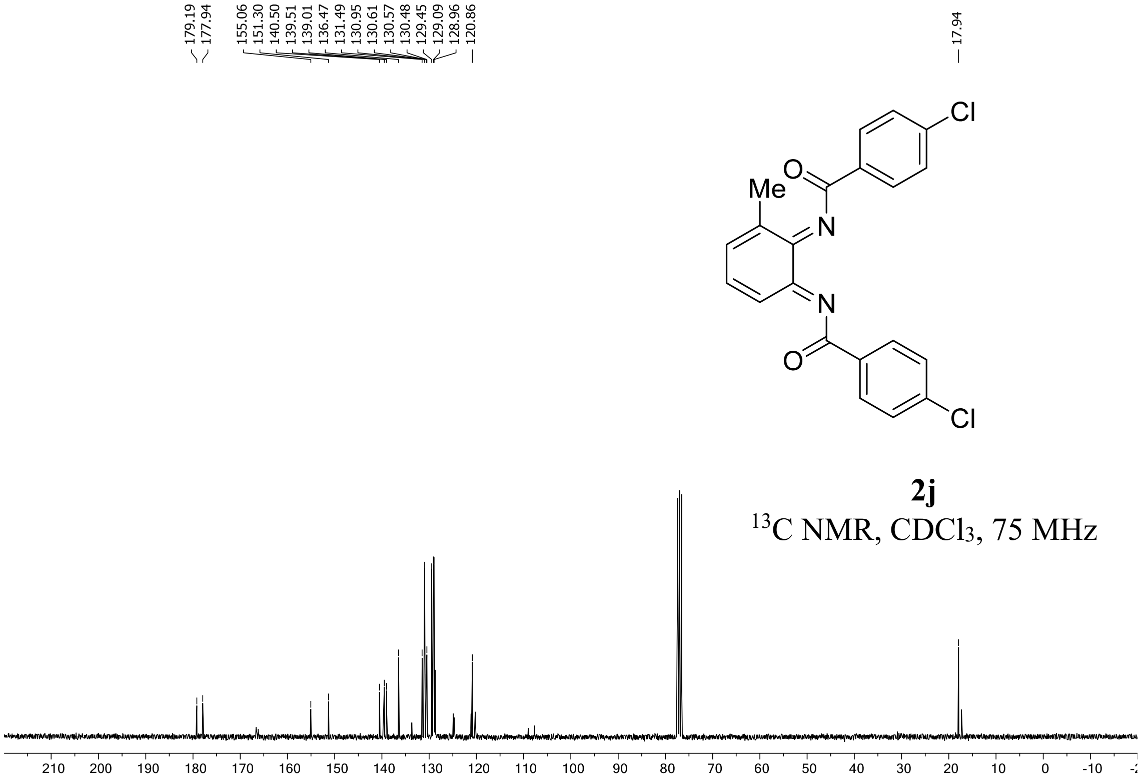

***N,N'*-(4-methylcyclohexa-3,5-diene-1,2-diylidene)bis(4-chlorobenzamide) (2k)**

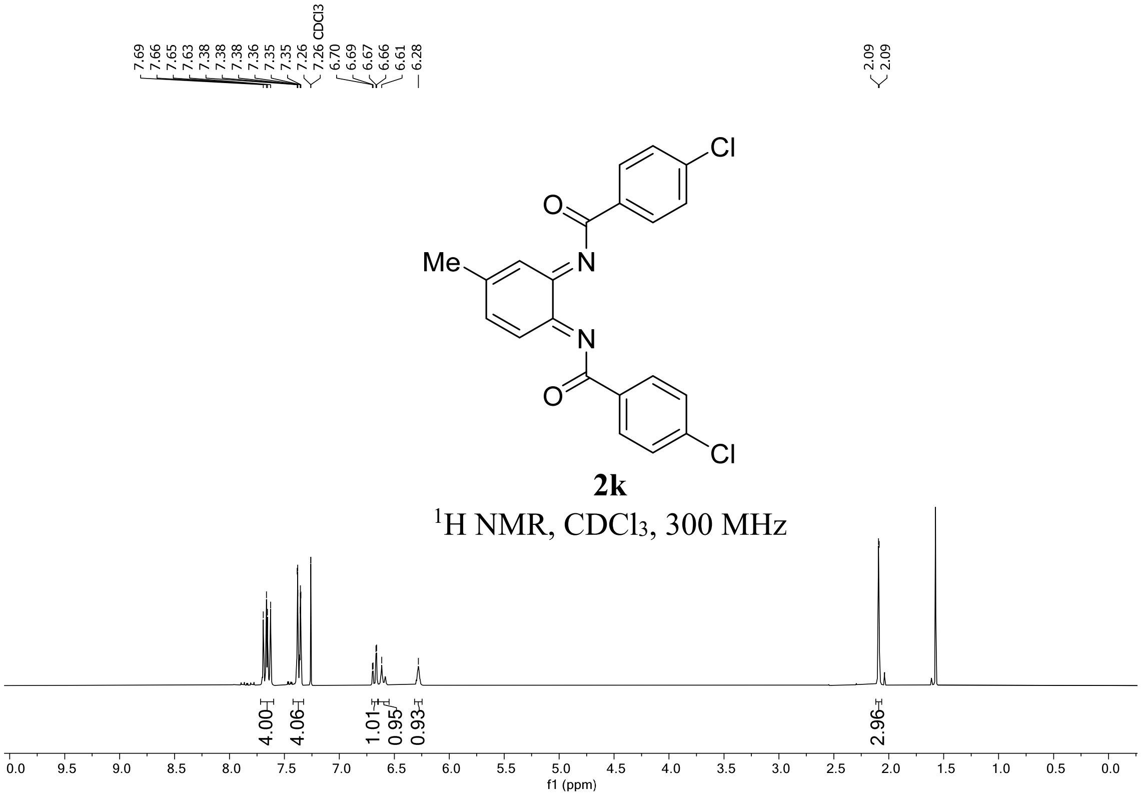

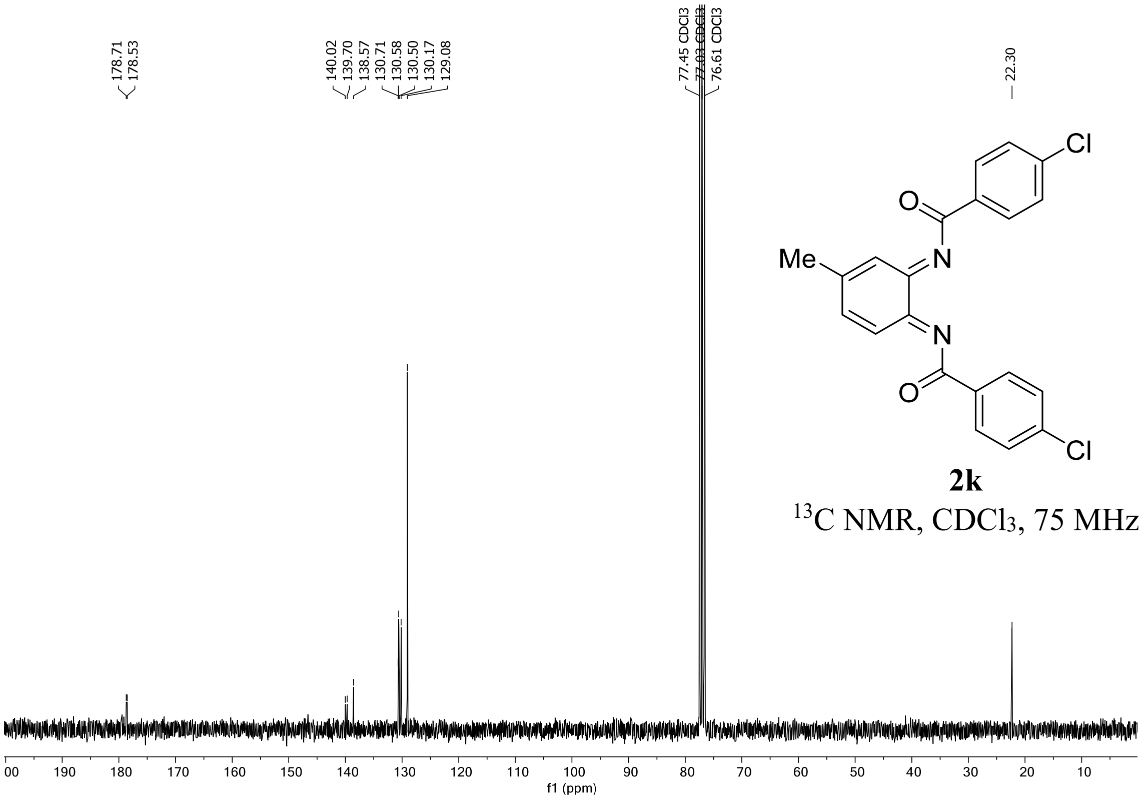

***N,N'*-(cyclohexa-3,5-diene-1,2-diylidene)bis(1-naphthamide) (2l)**

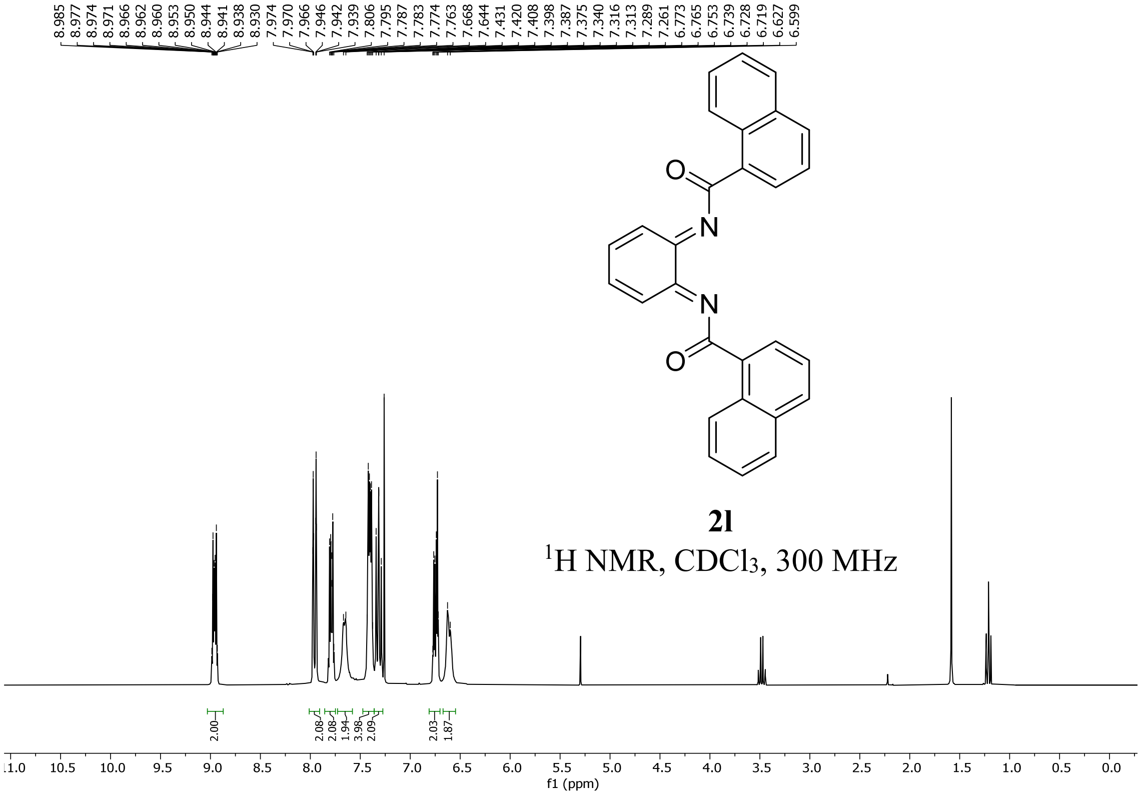

2l
^{1}H NMR, $CDCl_3$, 300 MHz

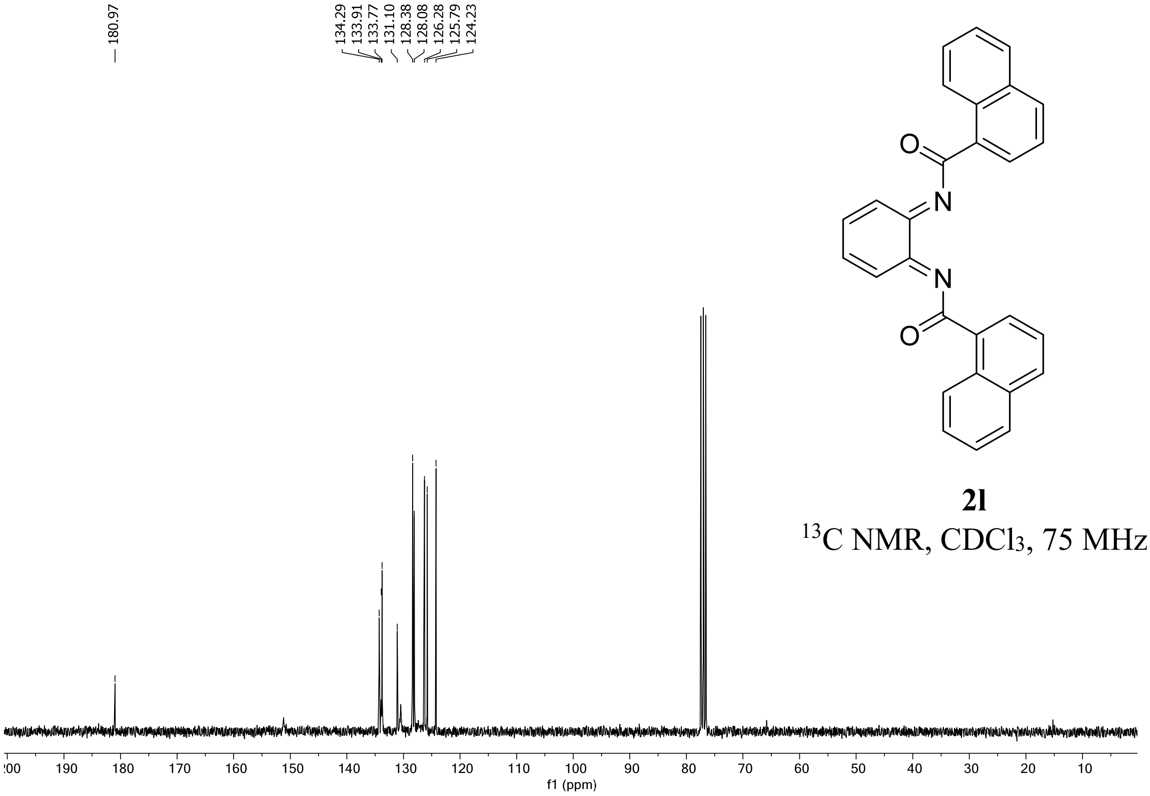

2l
^{13}C NMR, $CDCl_3$, 75 MHz

***N*,*N*'-(Cyclohexa-3,5-diene-1,2-diylidene)bis(4-methylbenzenesulfonamide) 2m**

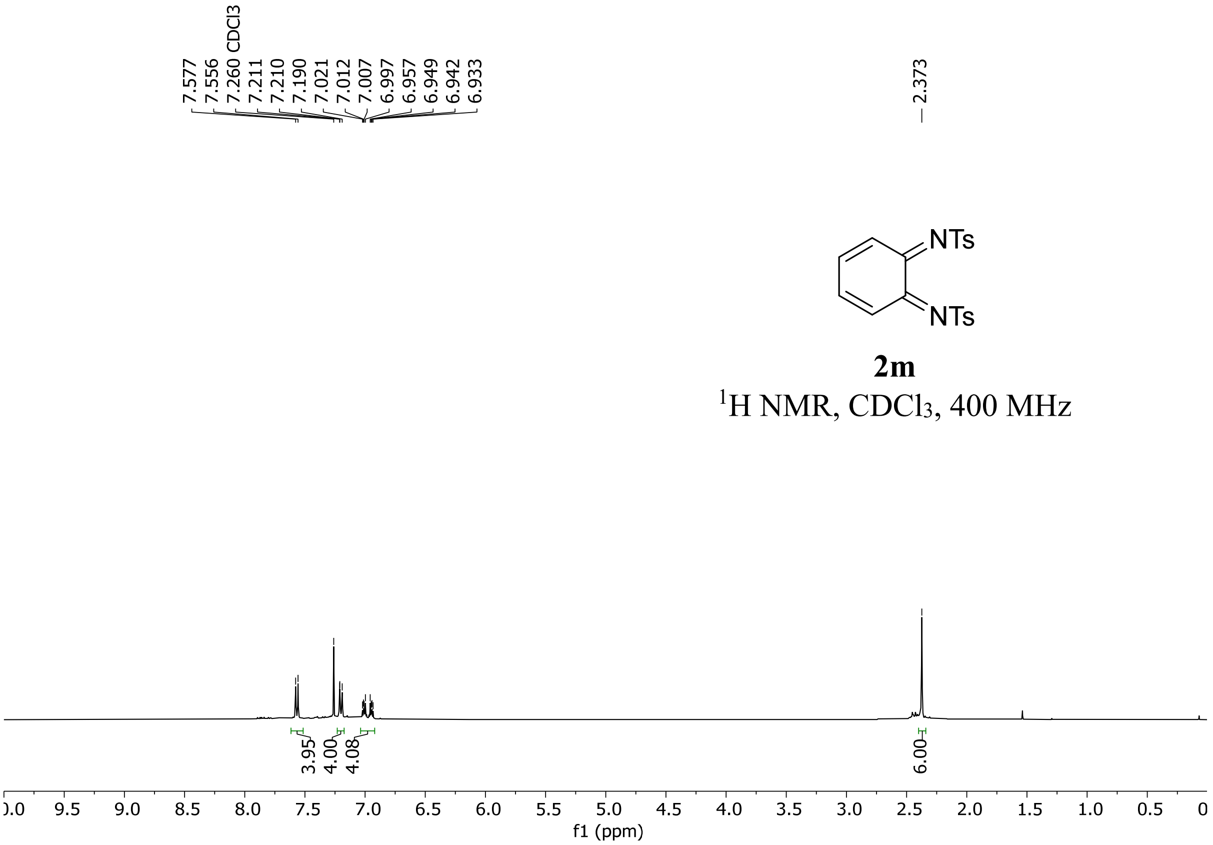

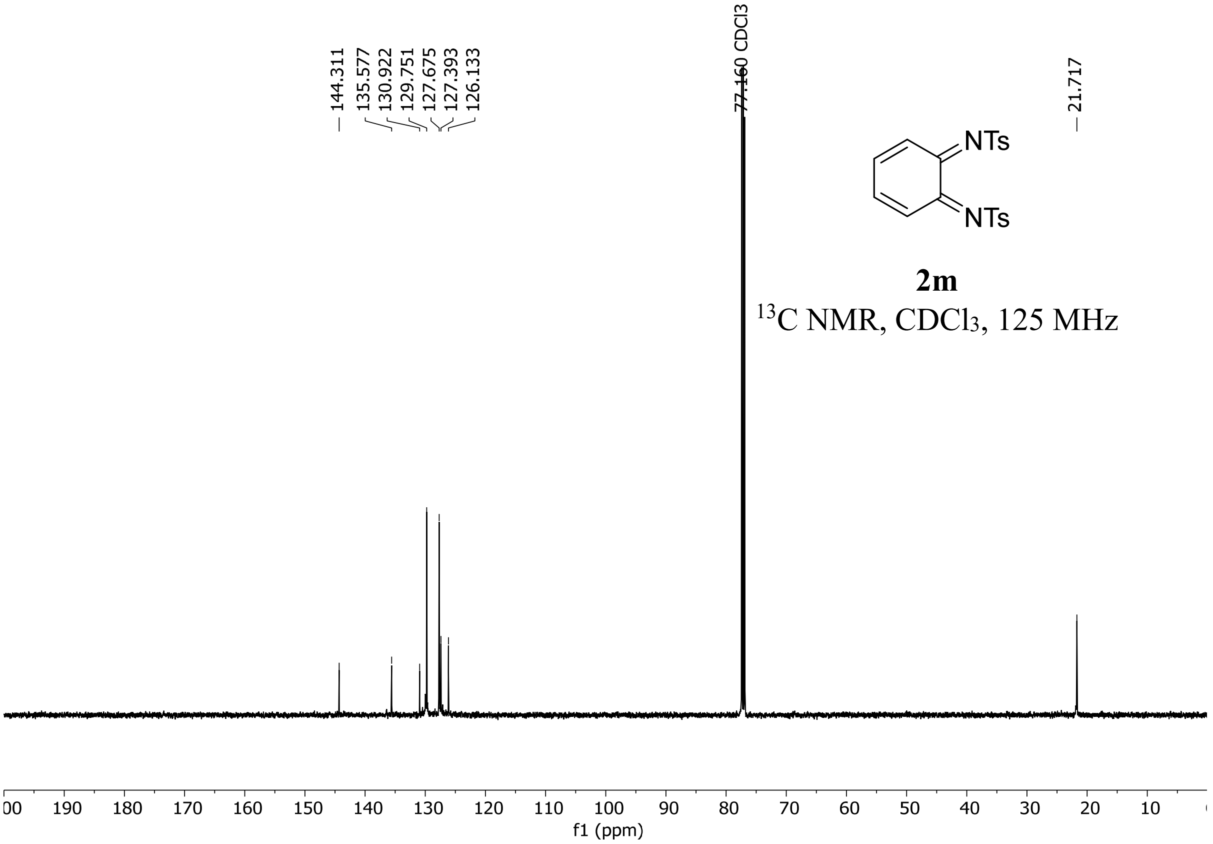

***N,N'*-(4-Hydroxycyclohexa-3,5-diene-1,2-diylidene)dibenzamide (2n)**

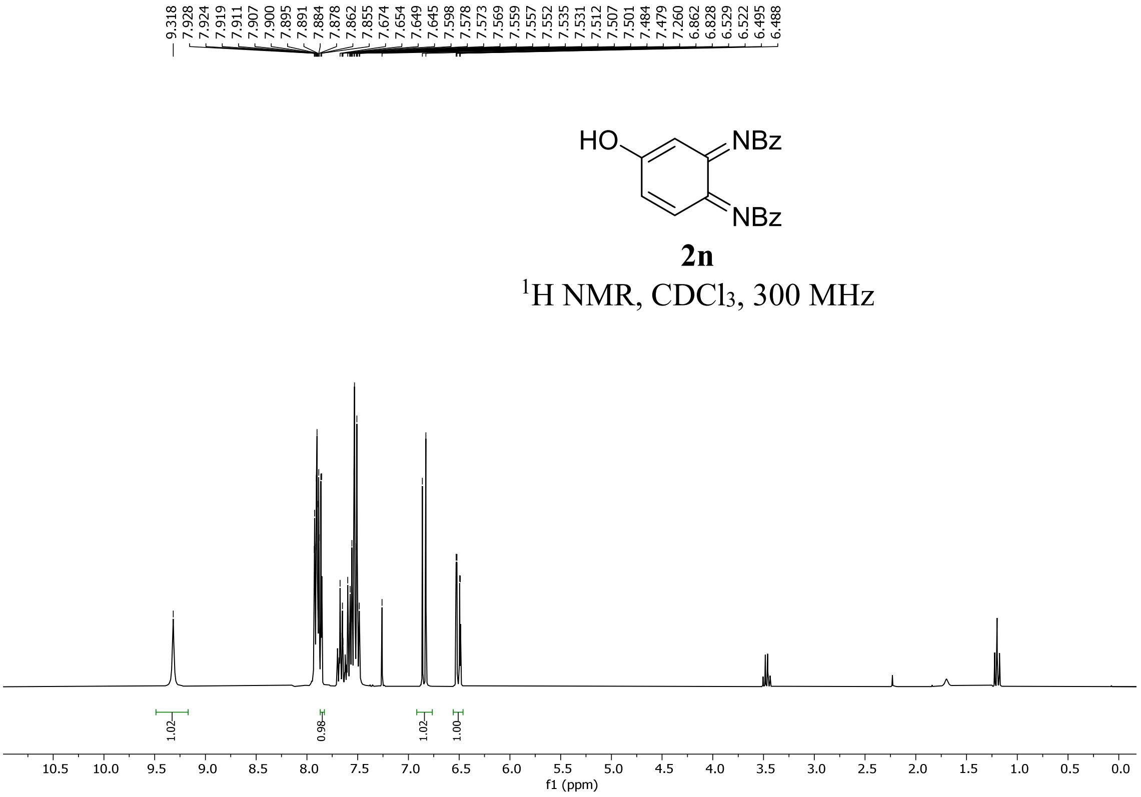

2n

^{1}H NMR, $CDCl_3$, 300 MHz

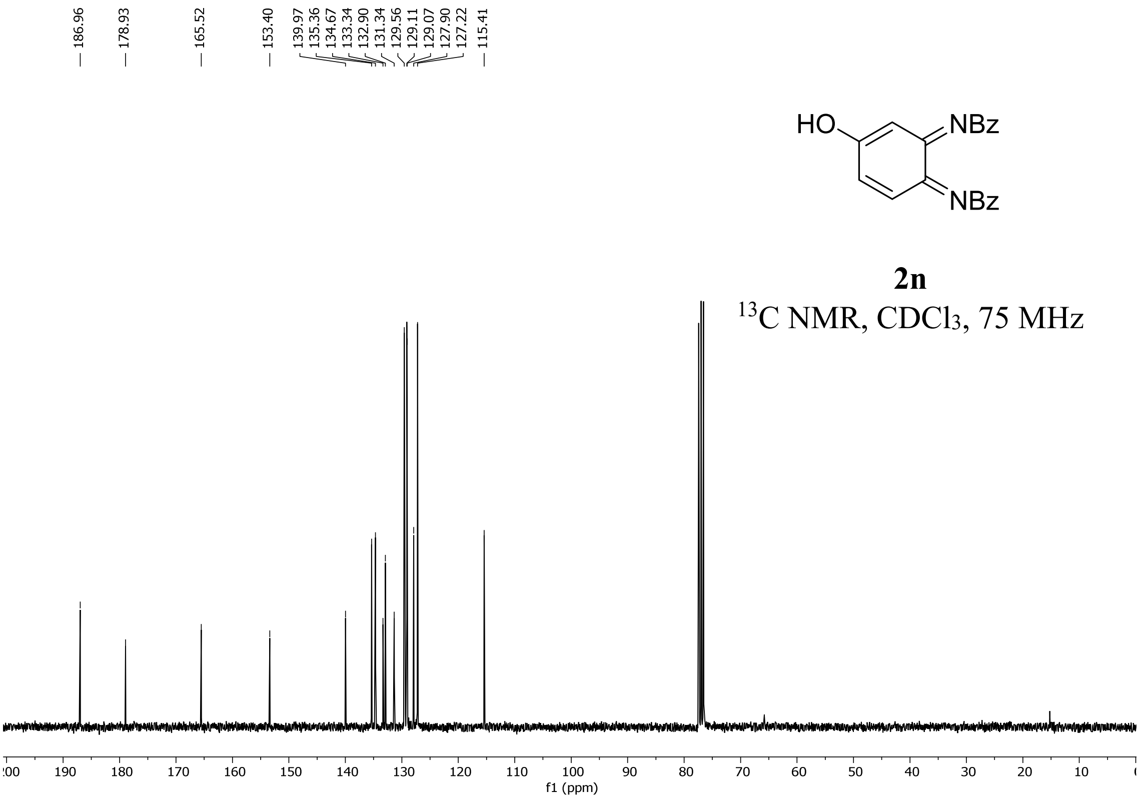

2n

^{13}C NMR, $CDCl_3$, 75 MHz

Methyl 2-(3,4-bis(benzamido)phenyl)-2-isocyano-2-phenylacetate (3aa)

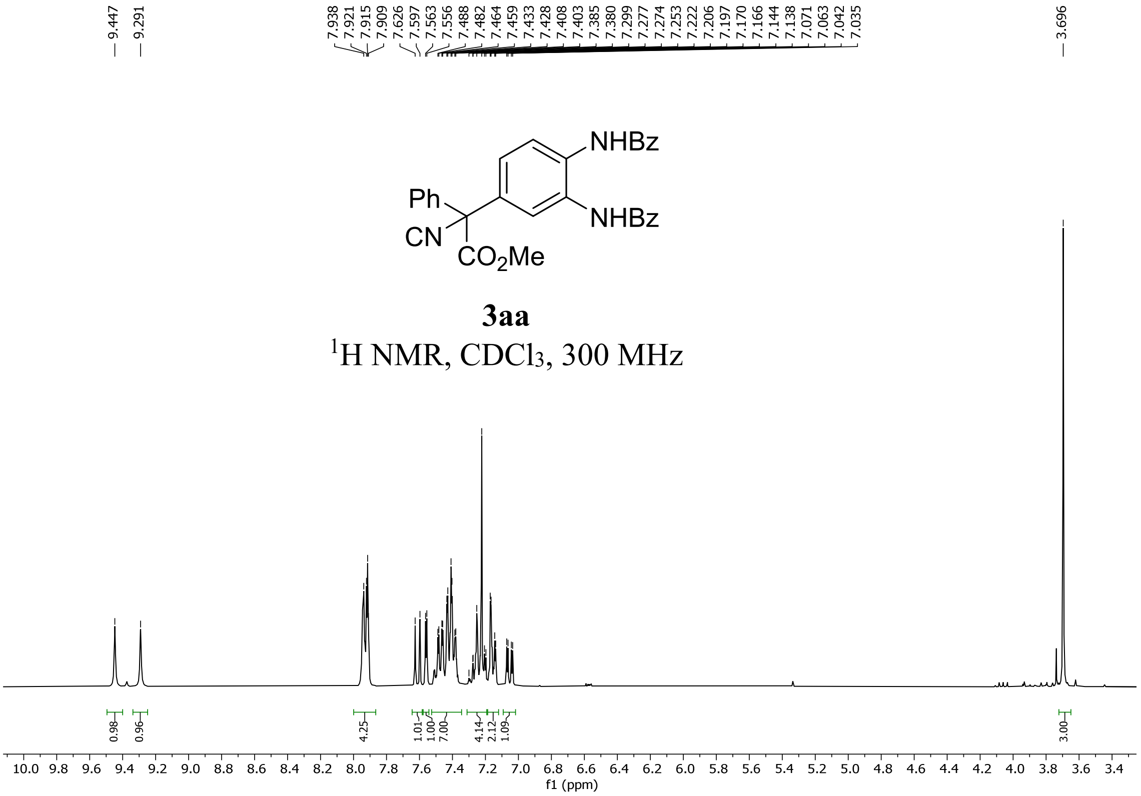

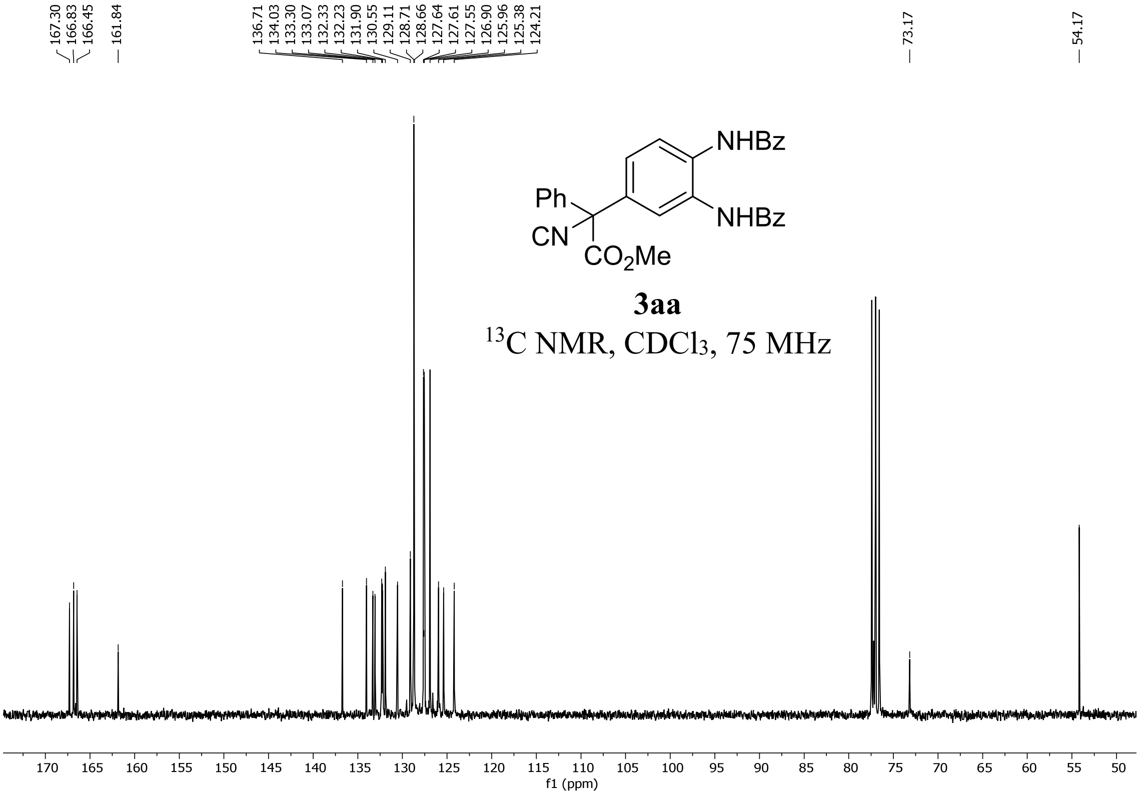

***tert*-Butyl 2-(3,4-bis(benzamido)phenyl)-2-isocyano-2-phenylacetate (3ba)**

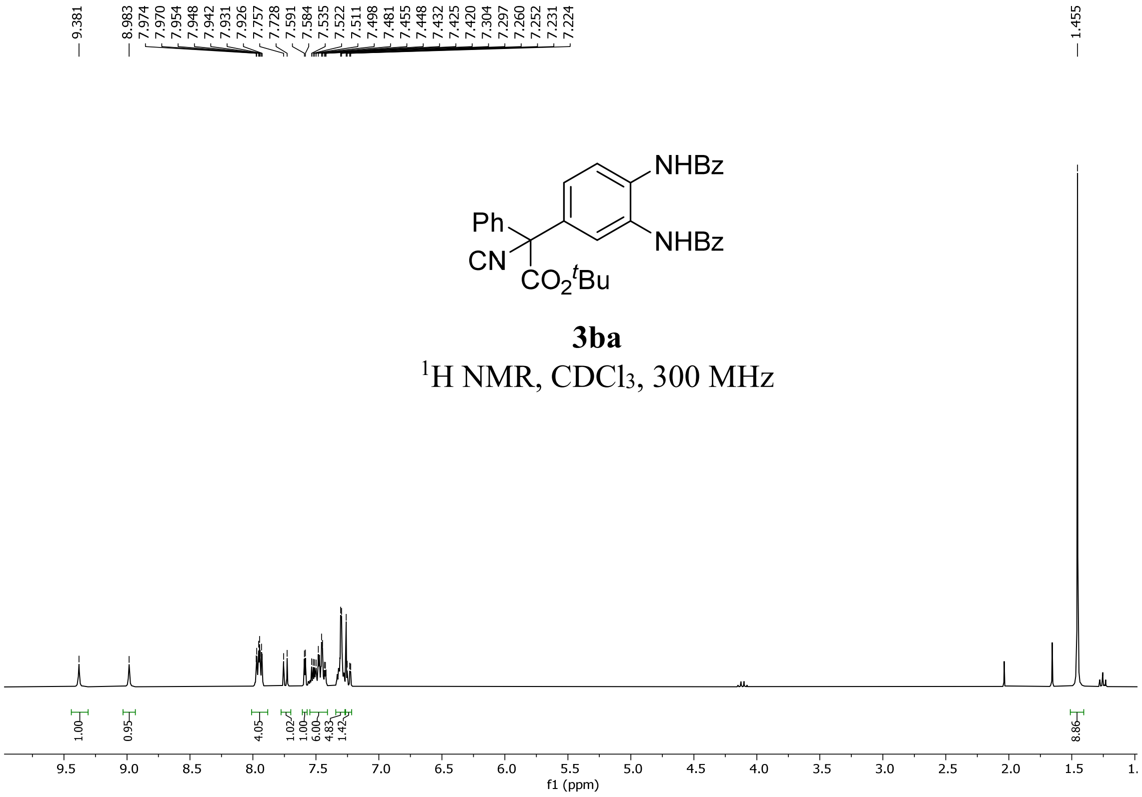

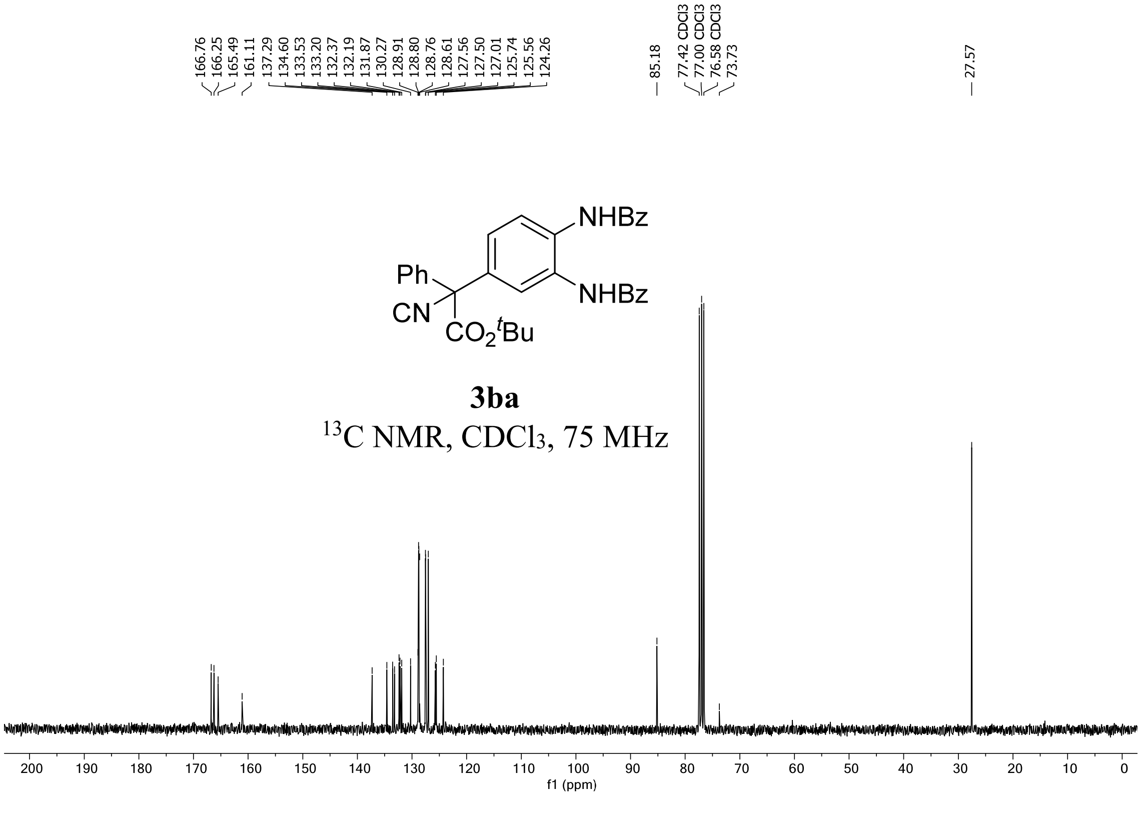

Methyl 2-(3,4-bis(benzamido)phenyl)-2-isocyano-2-(4-methoxyphenyl)acetate (3ca)

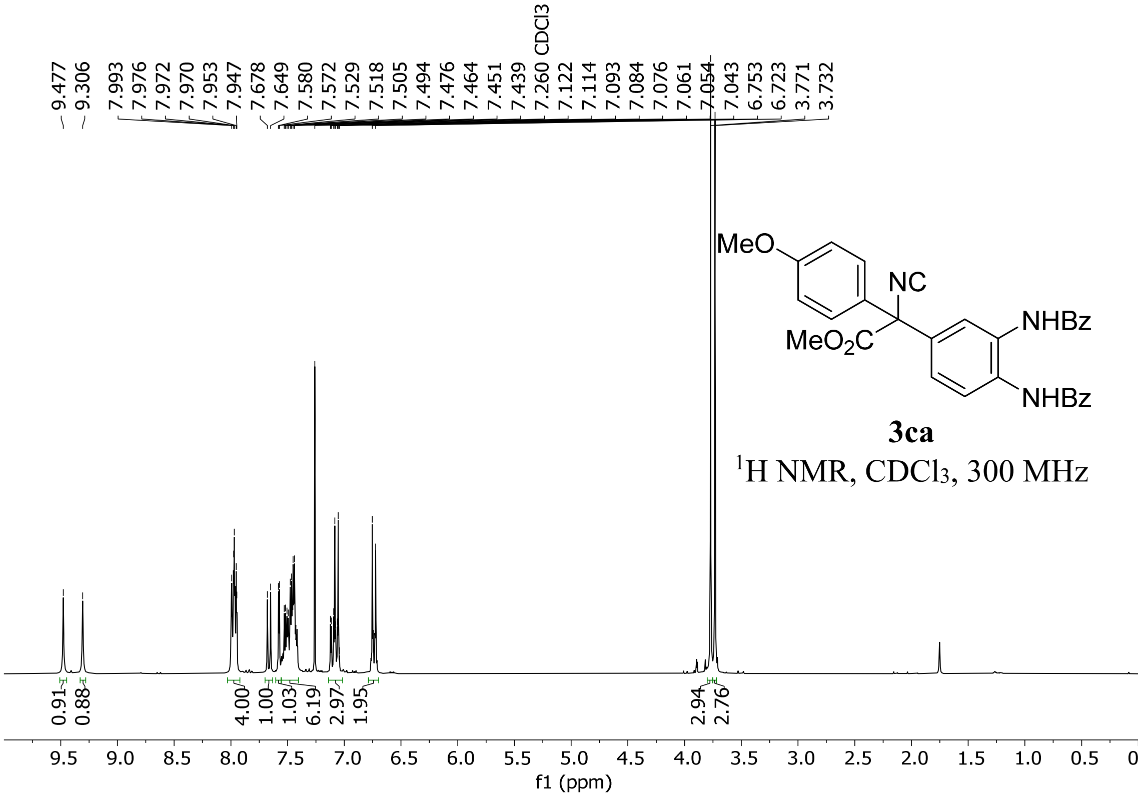

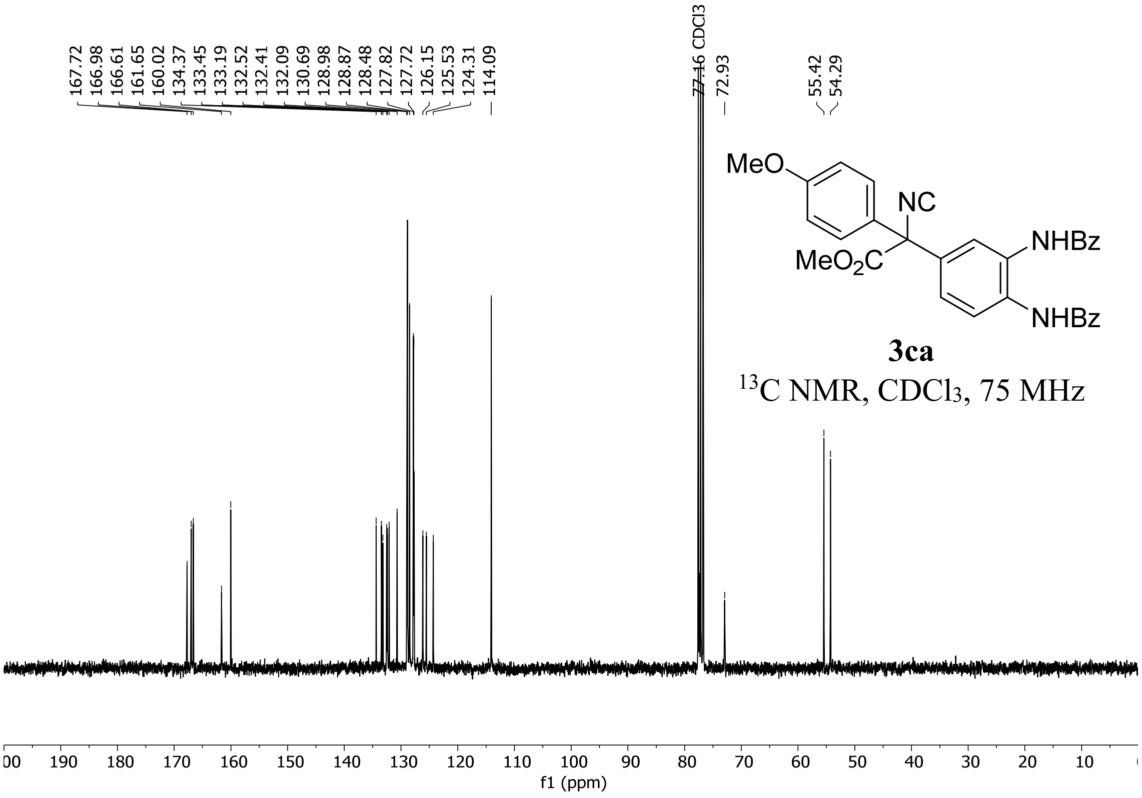

Methyl 2-(3,4-bis(benzamido)phenyl)-2-(4-chlorophenyl)-2-isocyanoacetate (3da)

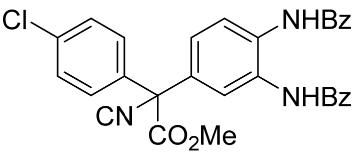

3da

^{1}H NMR, DMSO-d_6, 300 MHz

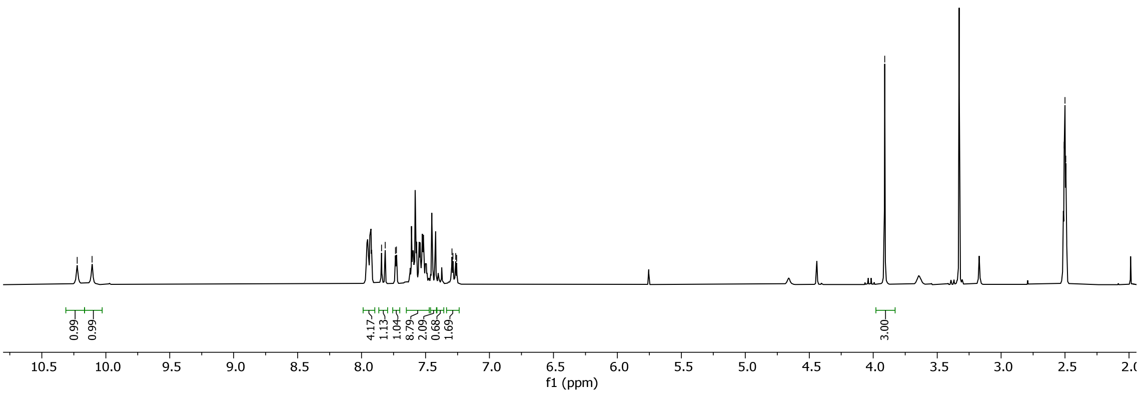

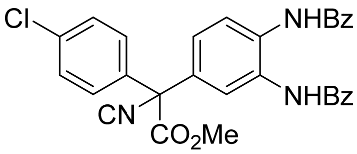

3da

^{13}C NMR, DMSO-d_6, 75 MHz

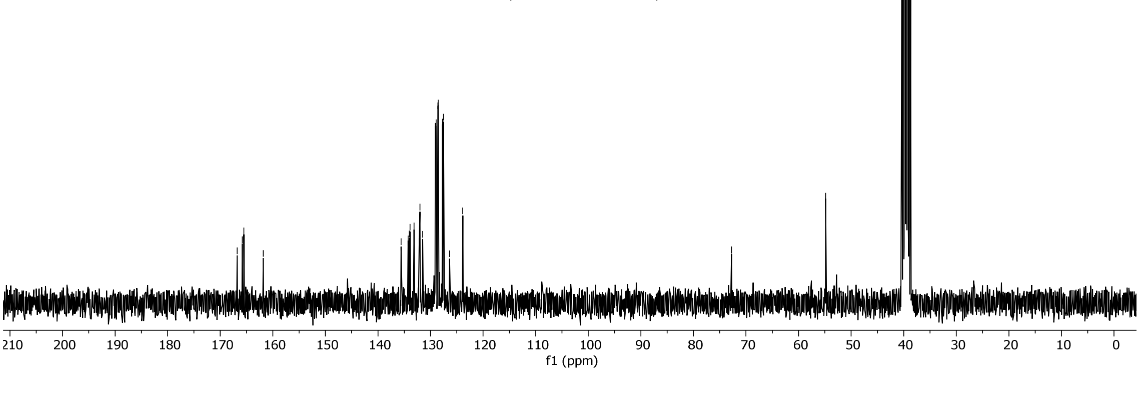

Methyl 2-(3,4-bis(benzamido)phenyl)-2-isocyano-2-(4-nitrophenyl)acetate (3ea)

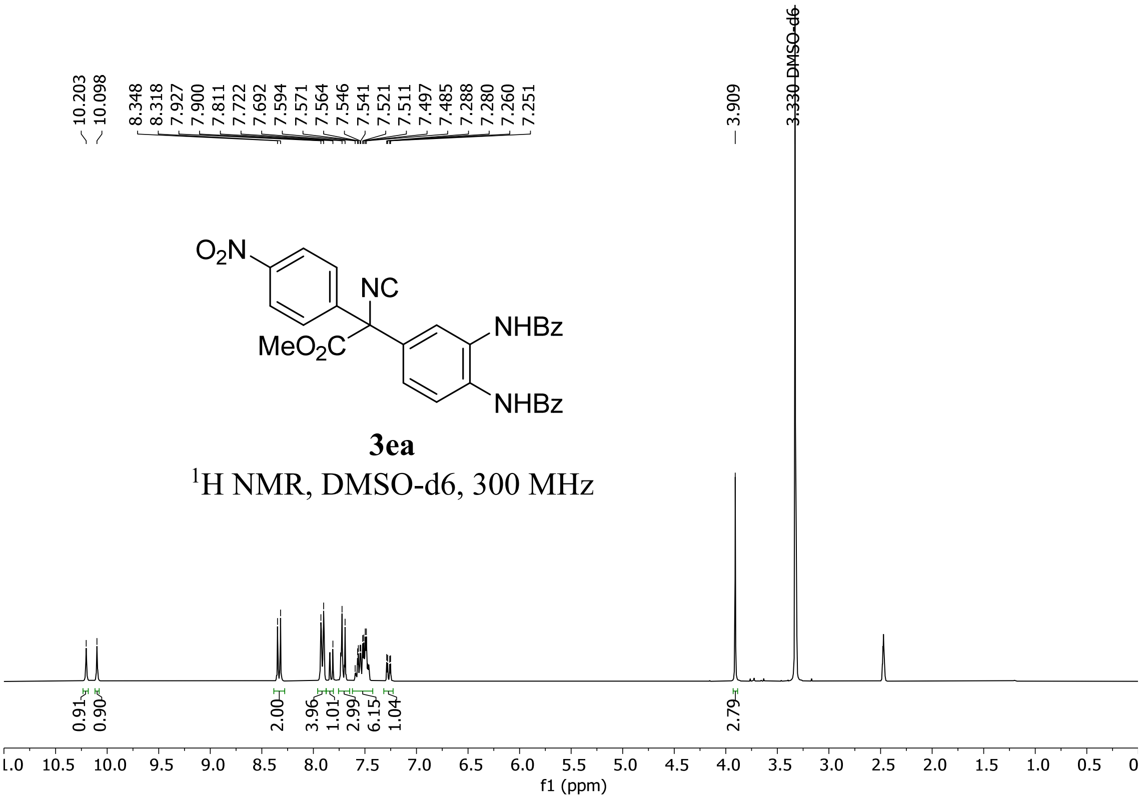

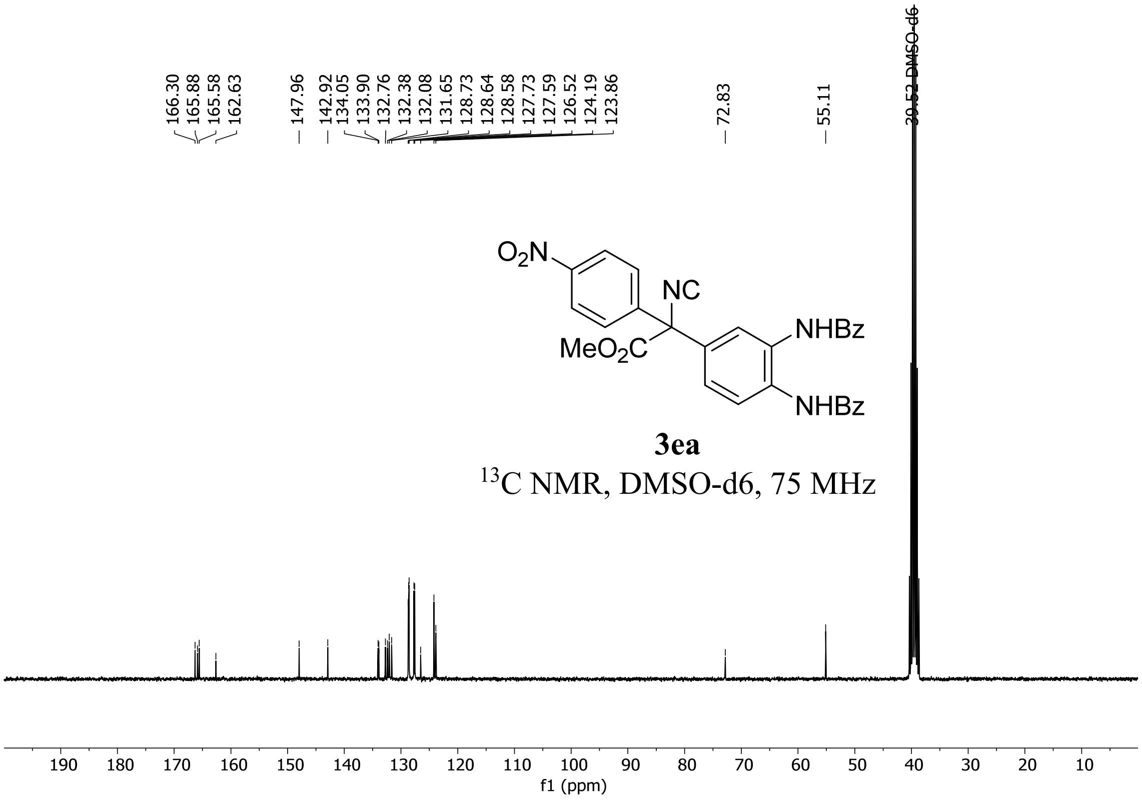

Methyl 2-(3,4-bis(benzamido)phenyl)-2-isocyano-2-(2-nitrophenyl)acetate (3fa)

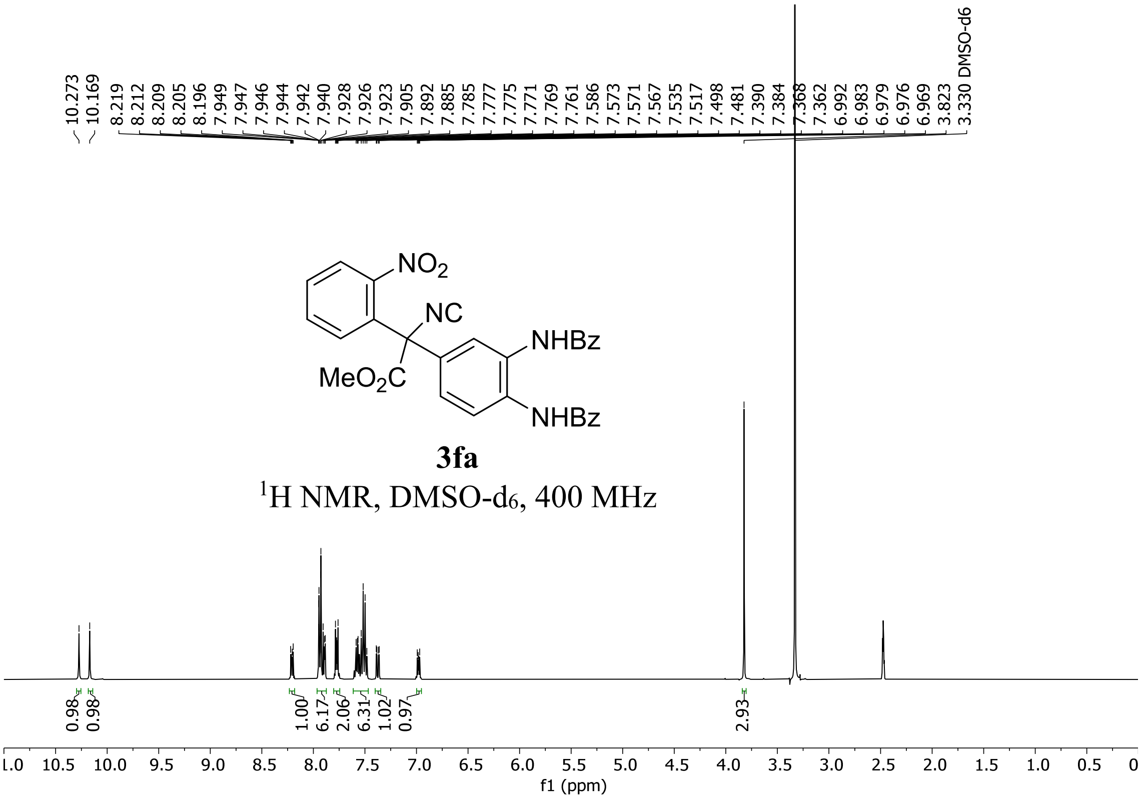

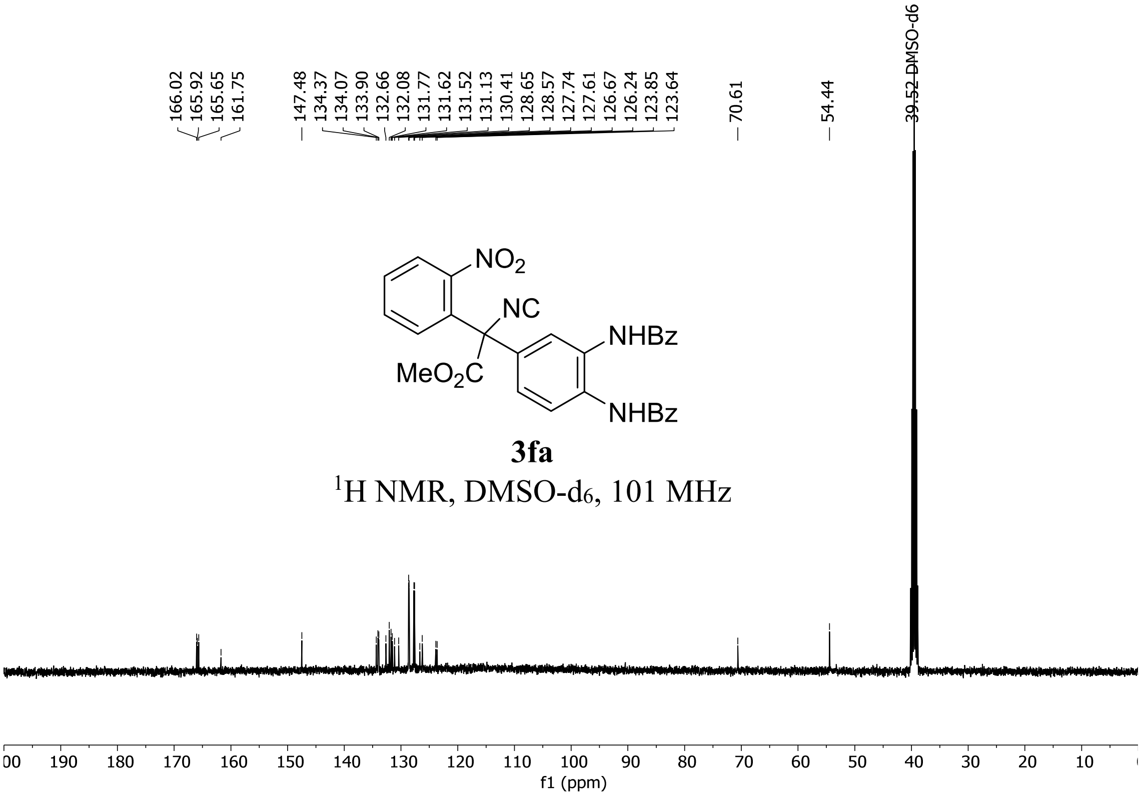

Methyl 2-(3,4-bis(benzamido)phenyl)-2-isocyanopropanoate (3ga)

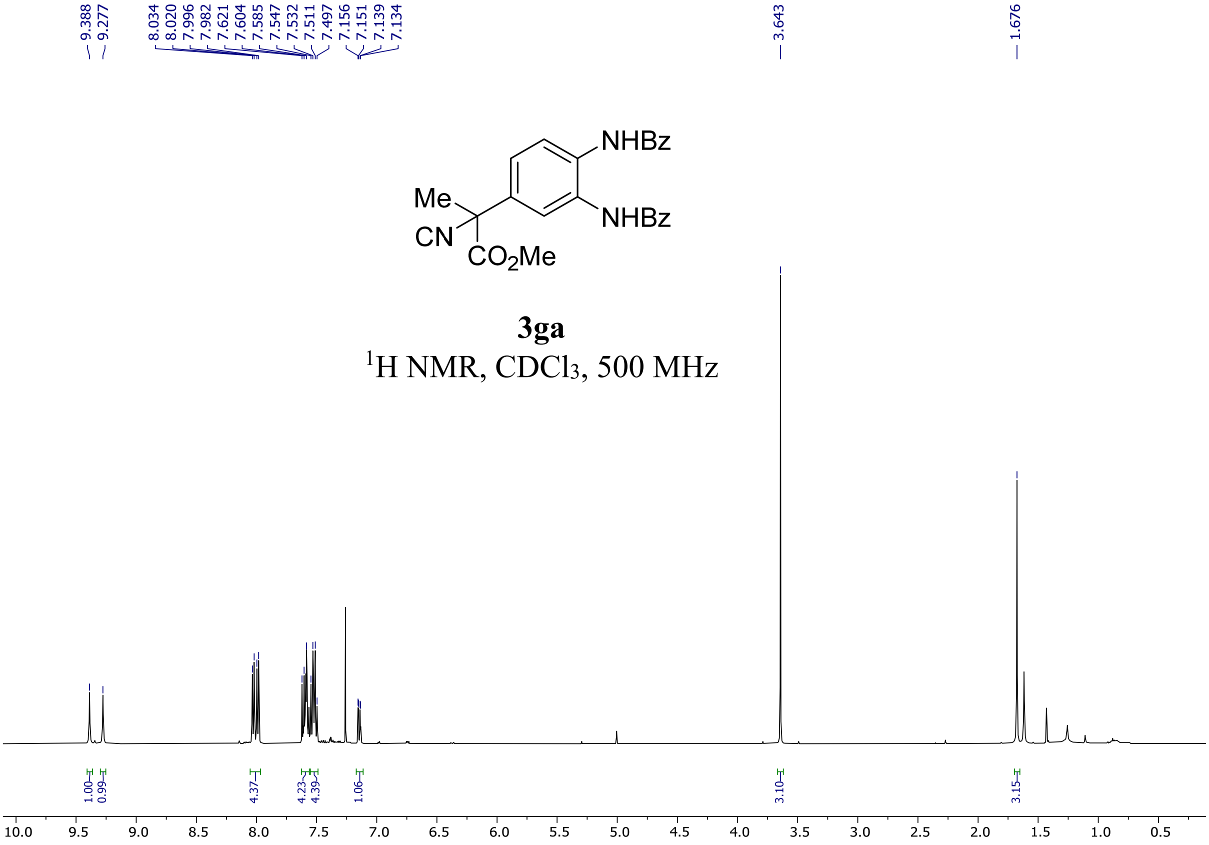

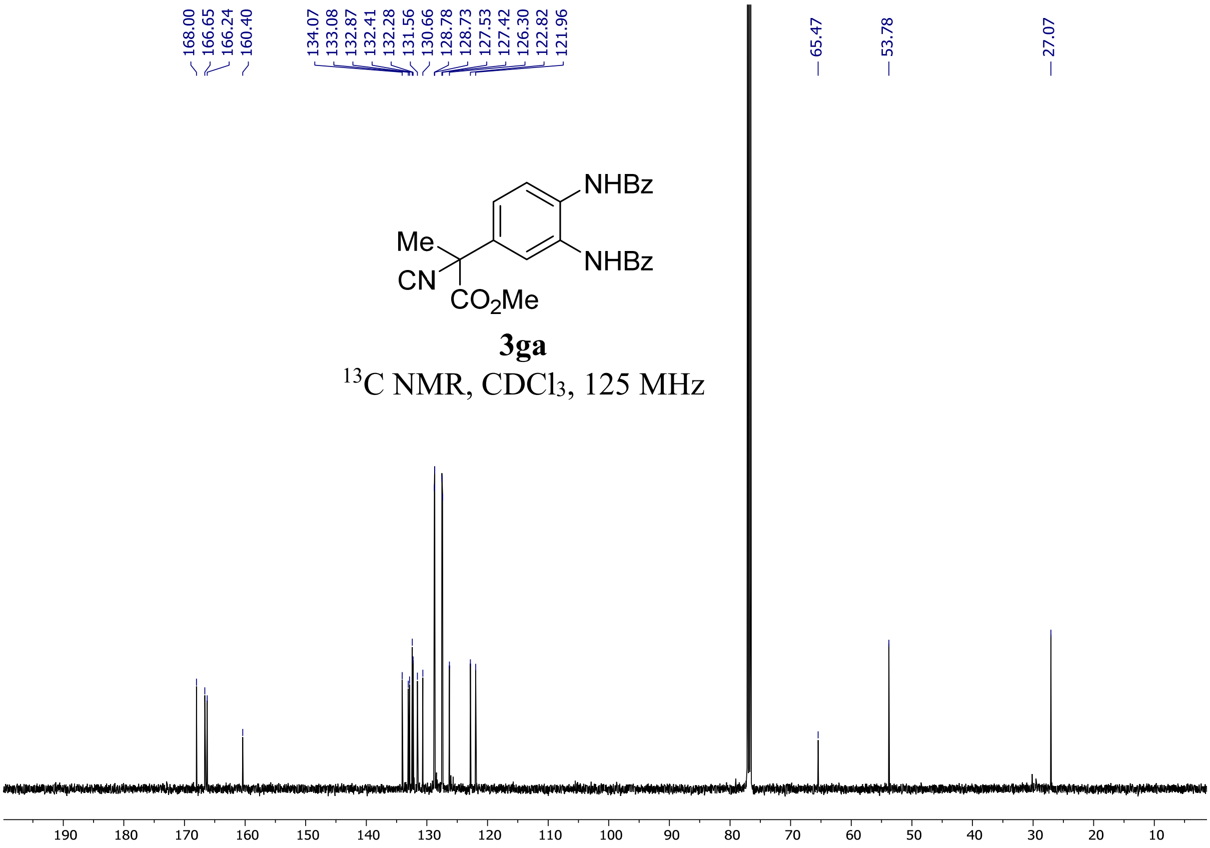

Methyl 2-(3,4-bis(benzamido)-5-methylphenyl)-2-isocyano-2-phenylacetate (3ab)

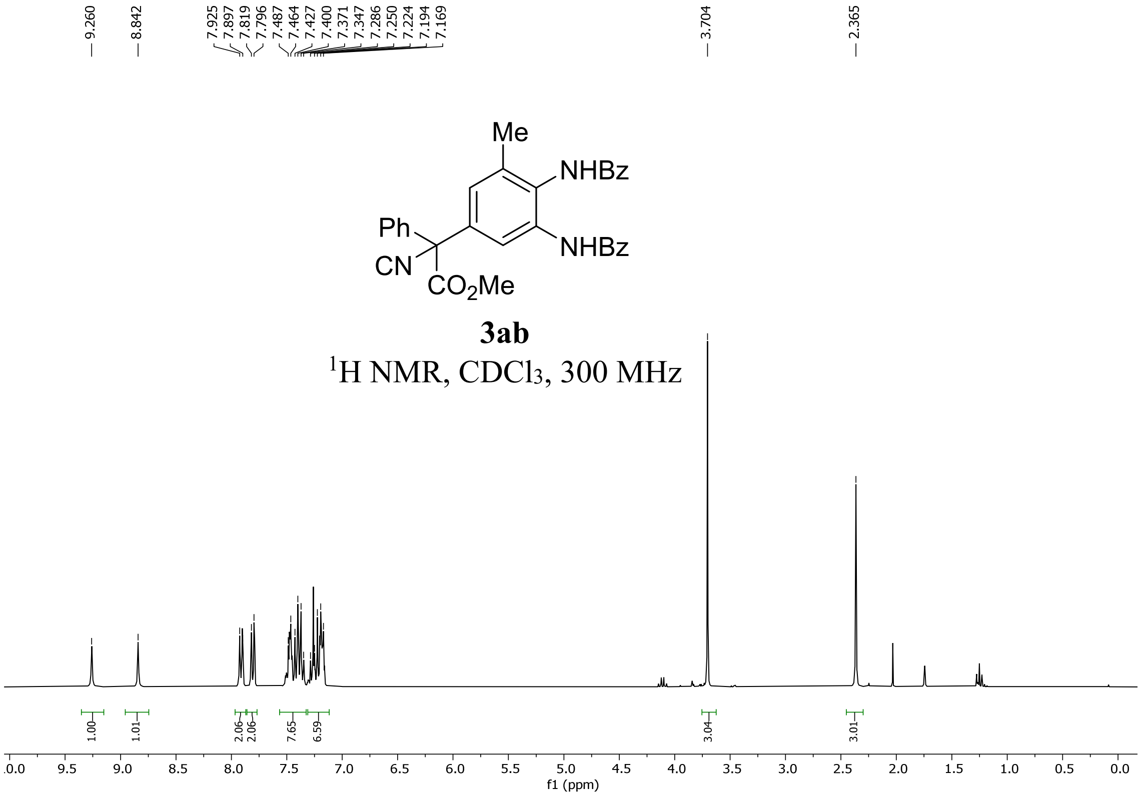

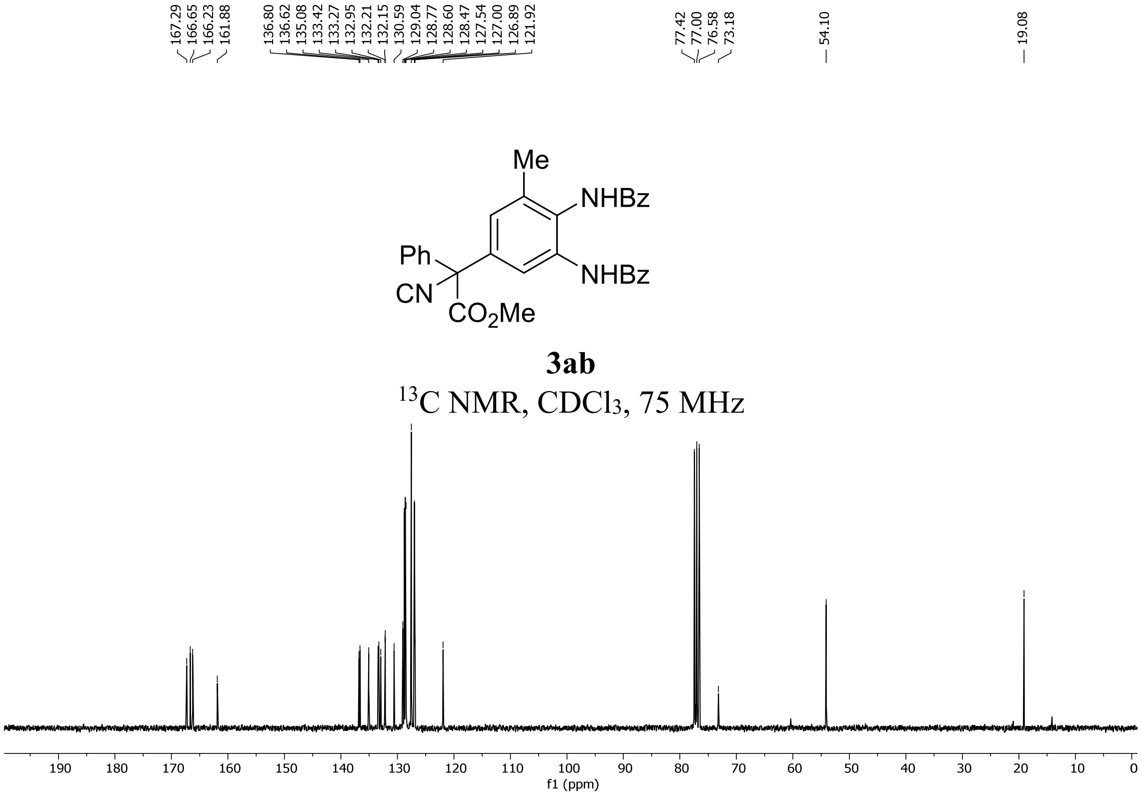

Methyl 2-(3,4-bis(benzamido)-5-fluorophenyl)-2-isocyano-2-phenylacetate (3ac)

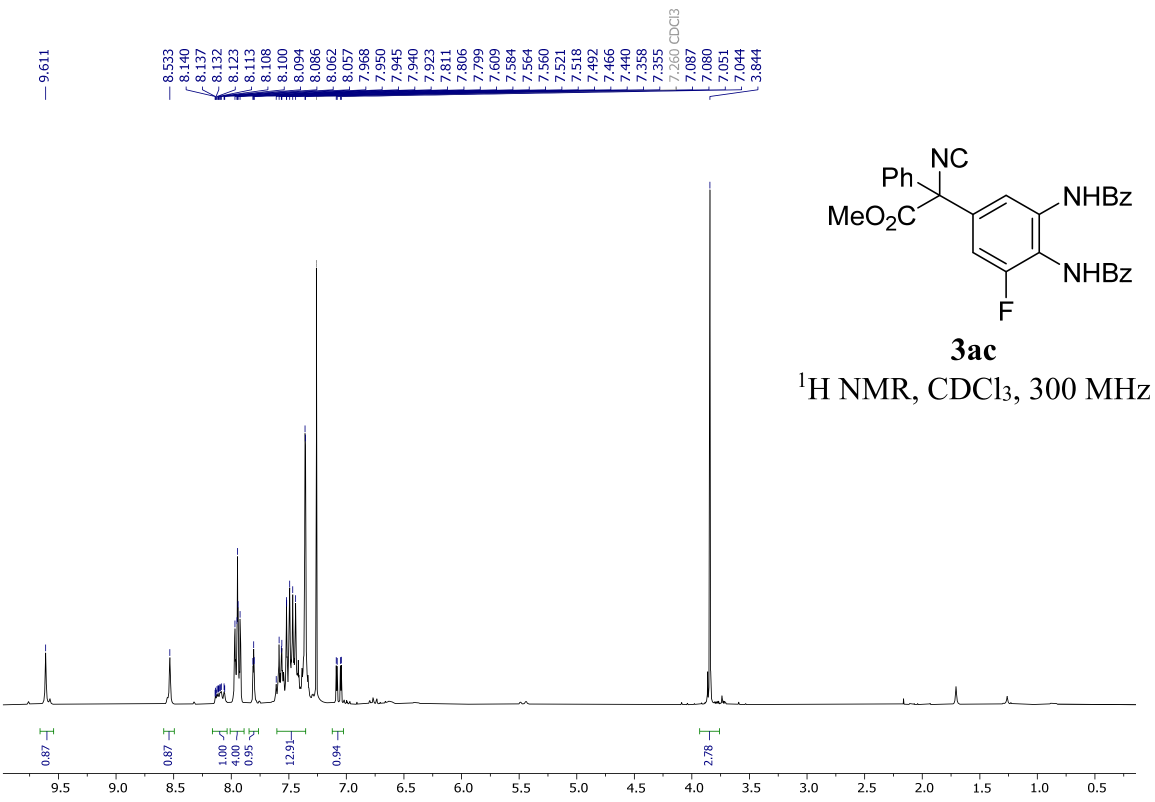

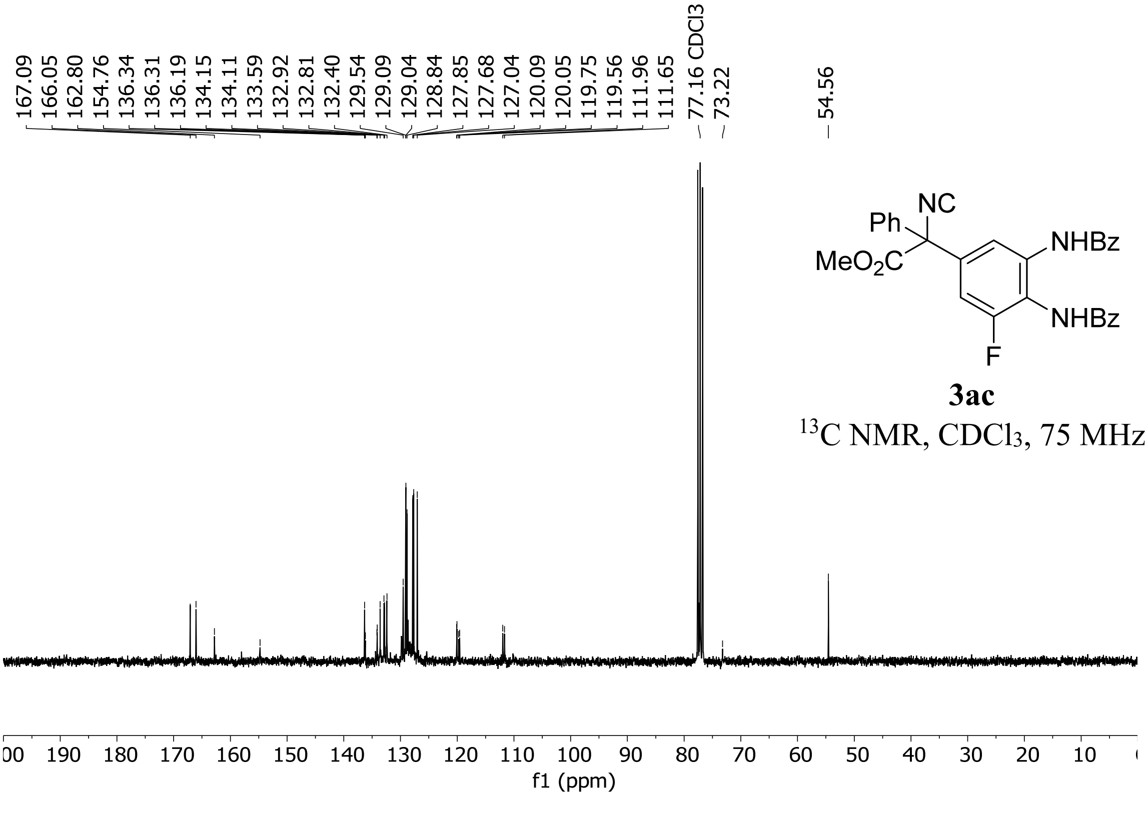

Methyl 2-(3,4-bis(benzamido)-5-chlorophenyl)-2-isocyano-2-phenylacetate (3ad)

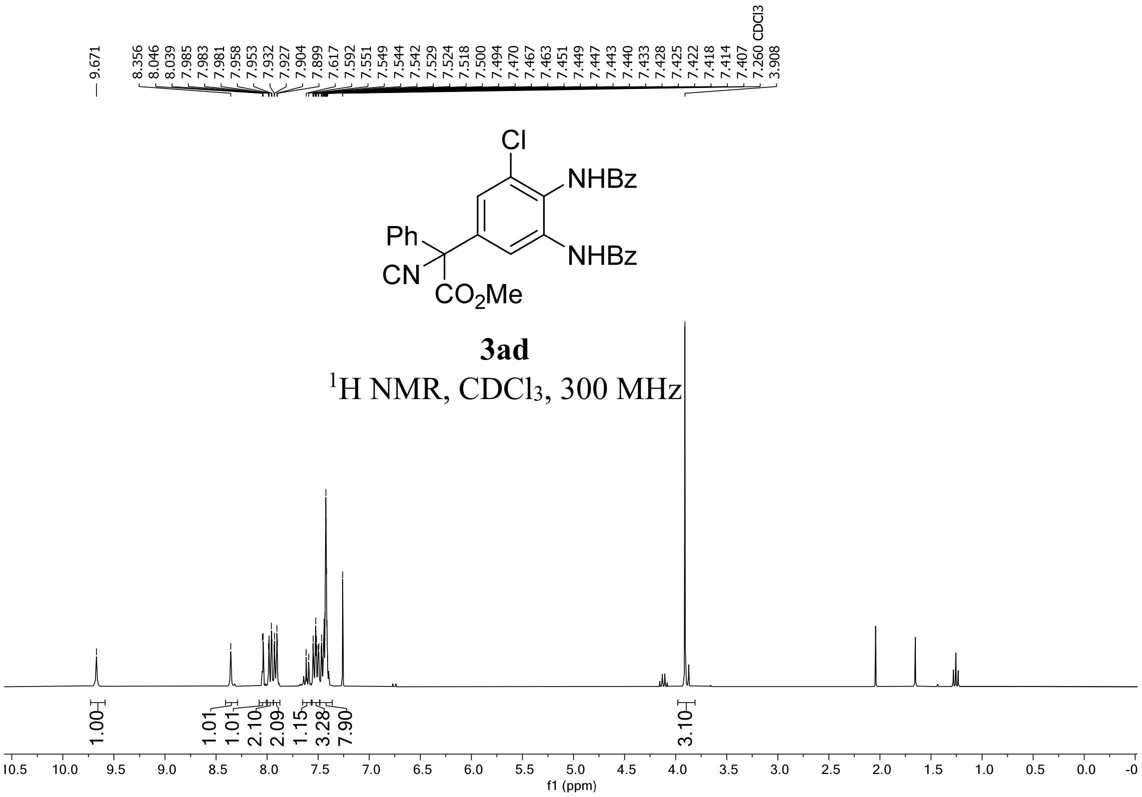

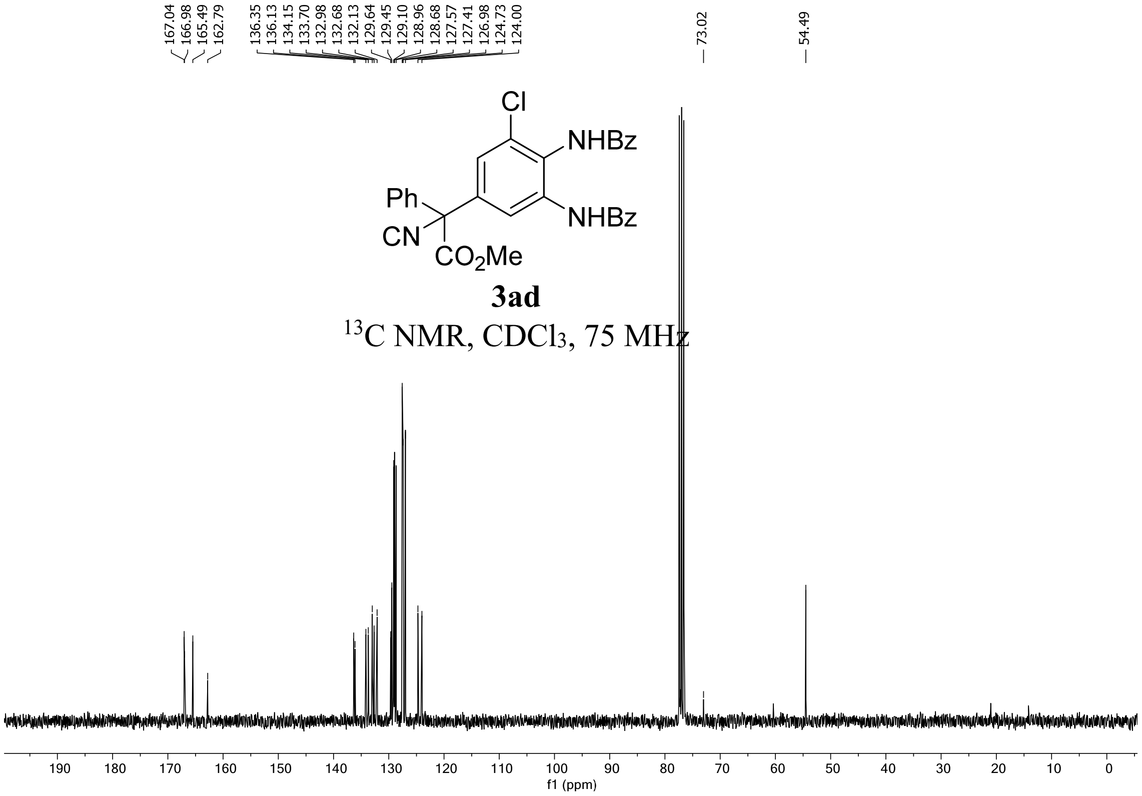

Methyl 2-(4,5-bis(benzamido)-2-chlorophenyl)-2-isocyano-2-phenylacetate (3ae)

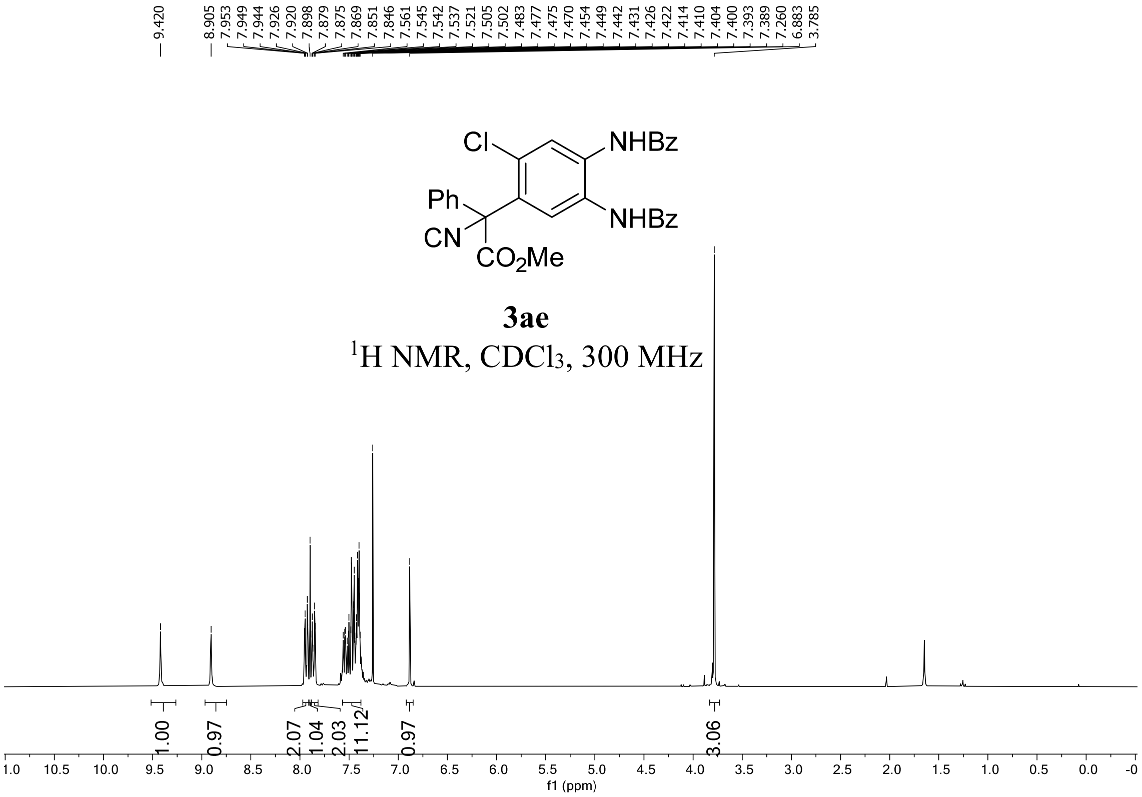

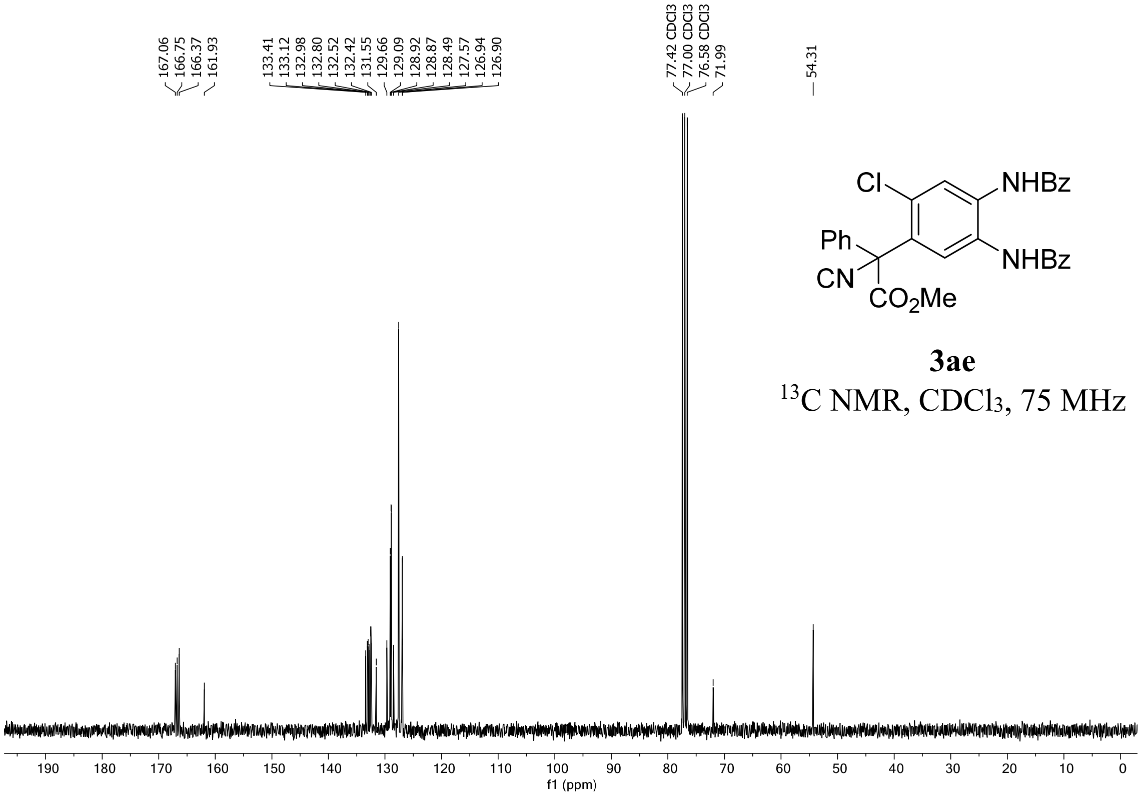

Methyl 2-(4,5-bis(benzamido)-2-bromophenyl)-2-isocyano-2-phenylacetate (3af)

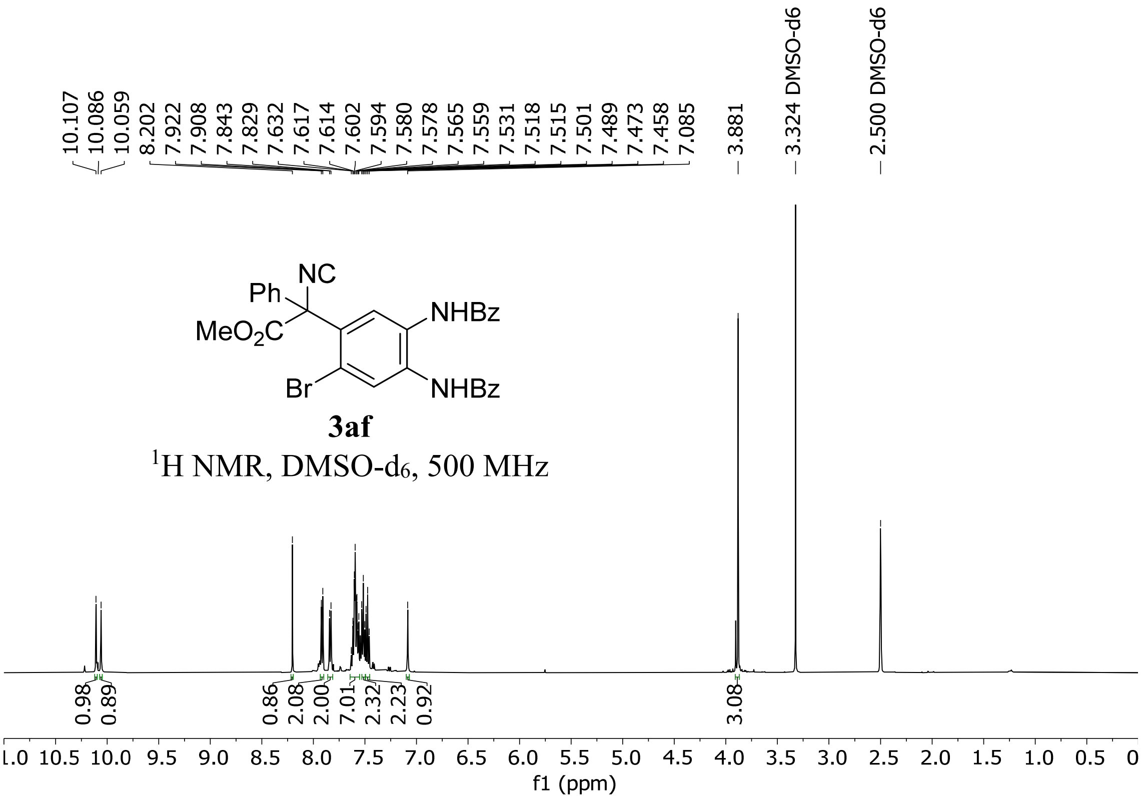

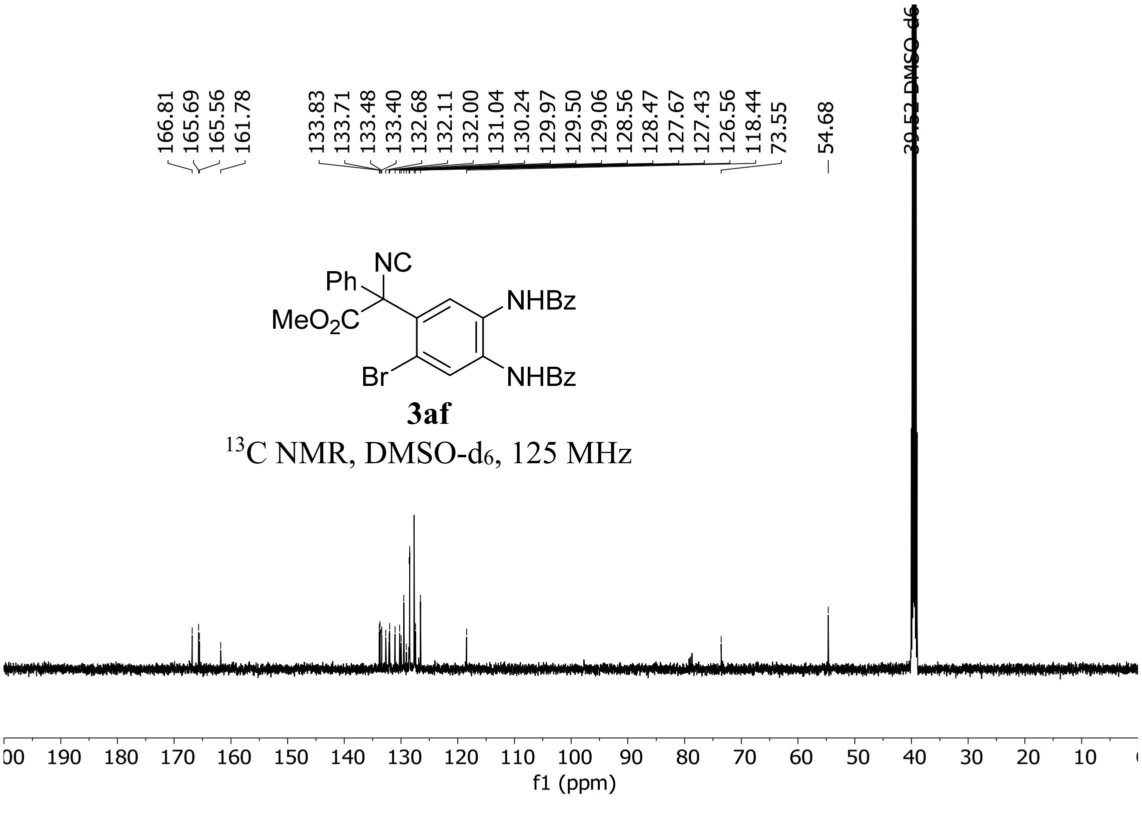

Methyl 2-(4,5-bis(benzamido)-2,3-dimethylphenyl)-2-isocyano-2-phenylacetate (3ag)

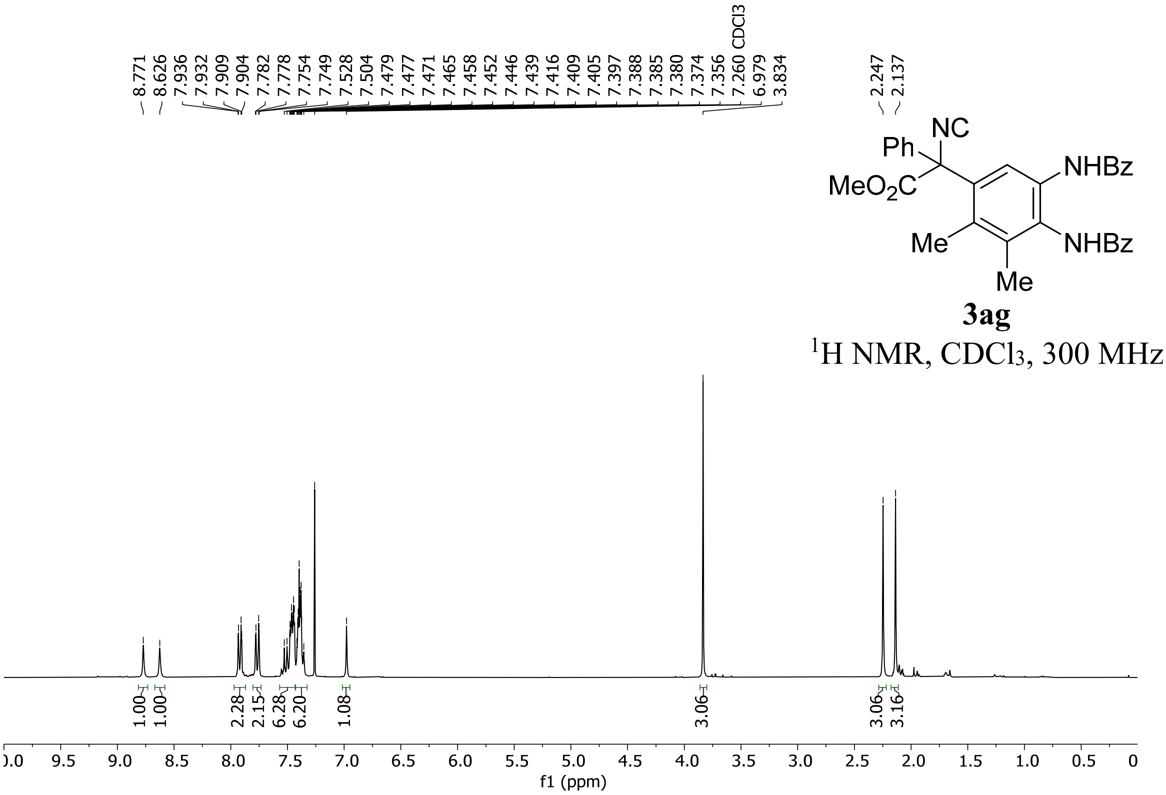

3ag
^{1}H NMR, $CDCl_3$, 300 MHz

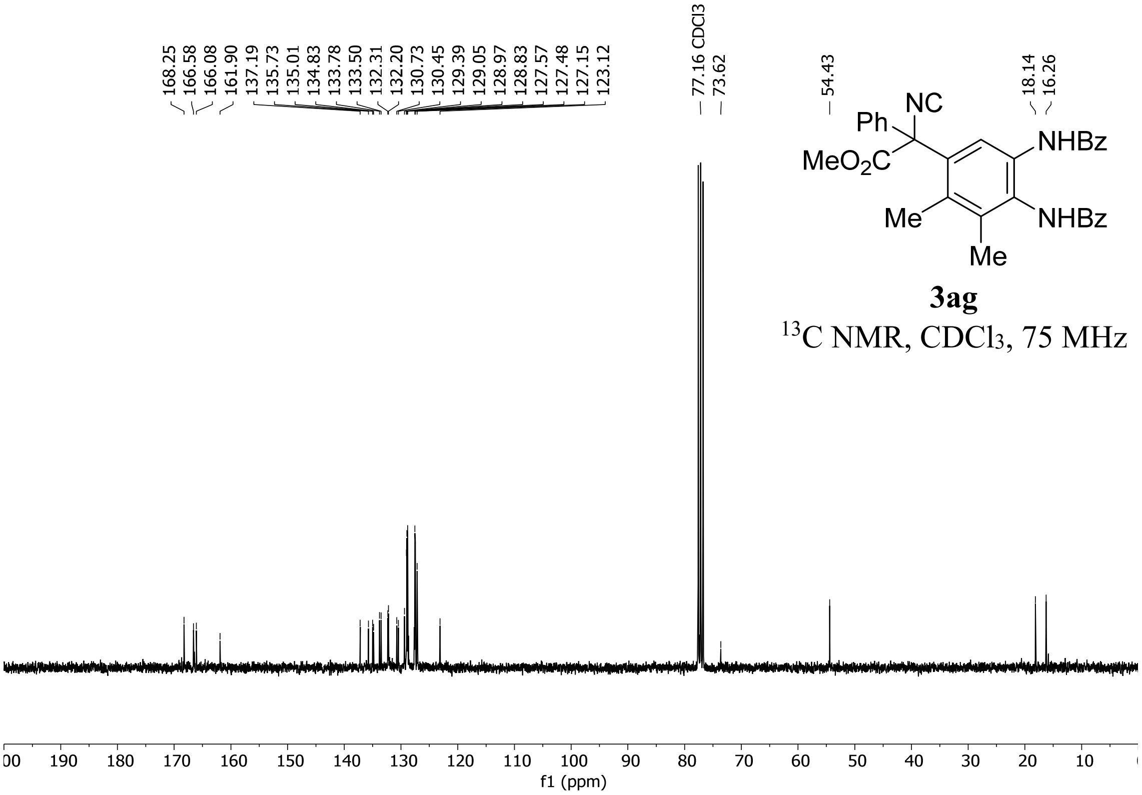

3ag
^{13}C NMR, $CDCl_3$, 75 MHz

Methyl 2-(4,5-bis(benzamido)-2,3-difluorophenyl)-2-isocyano-2-phenylacetate (3ah)

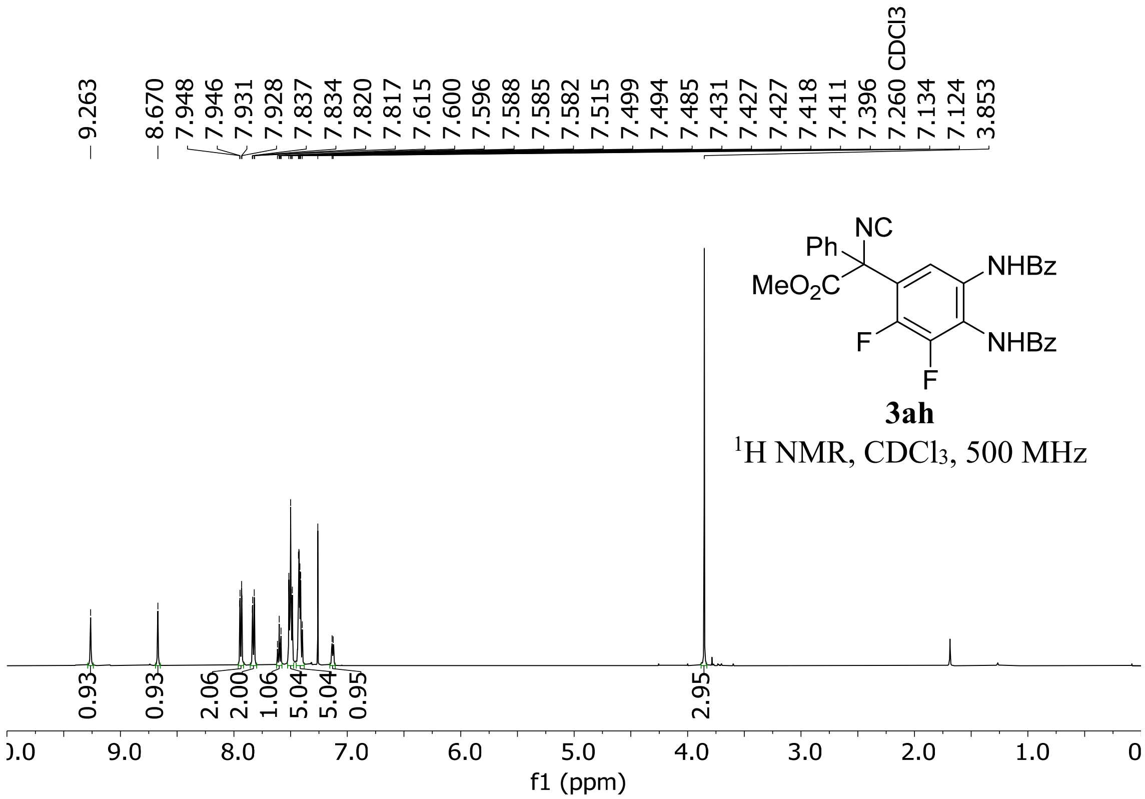

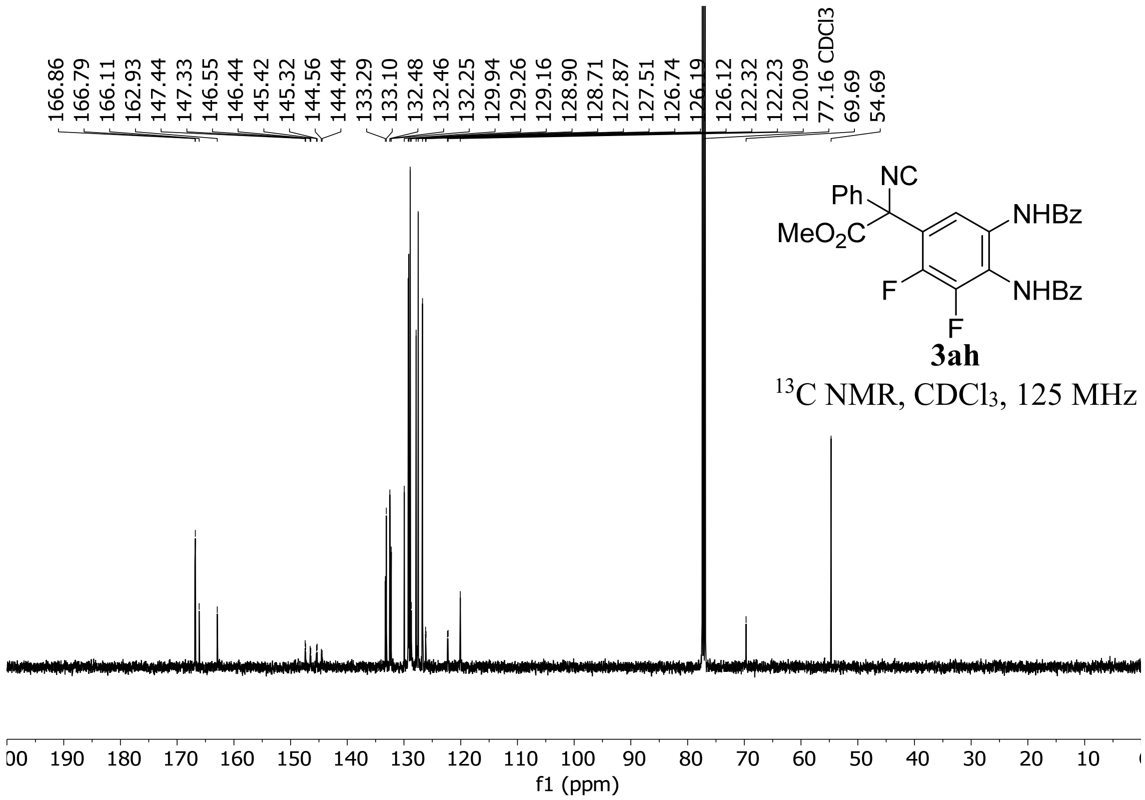

Methyl 2-(3,4-bis(4-chlorobenzamido)phenyl)-2-isocyano-2-phenylacetate (3ai)

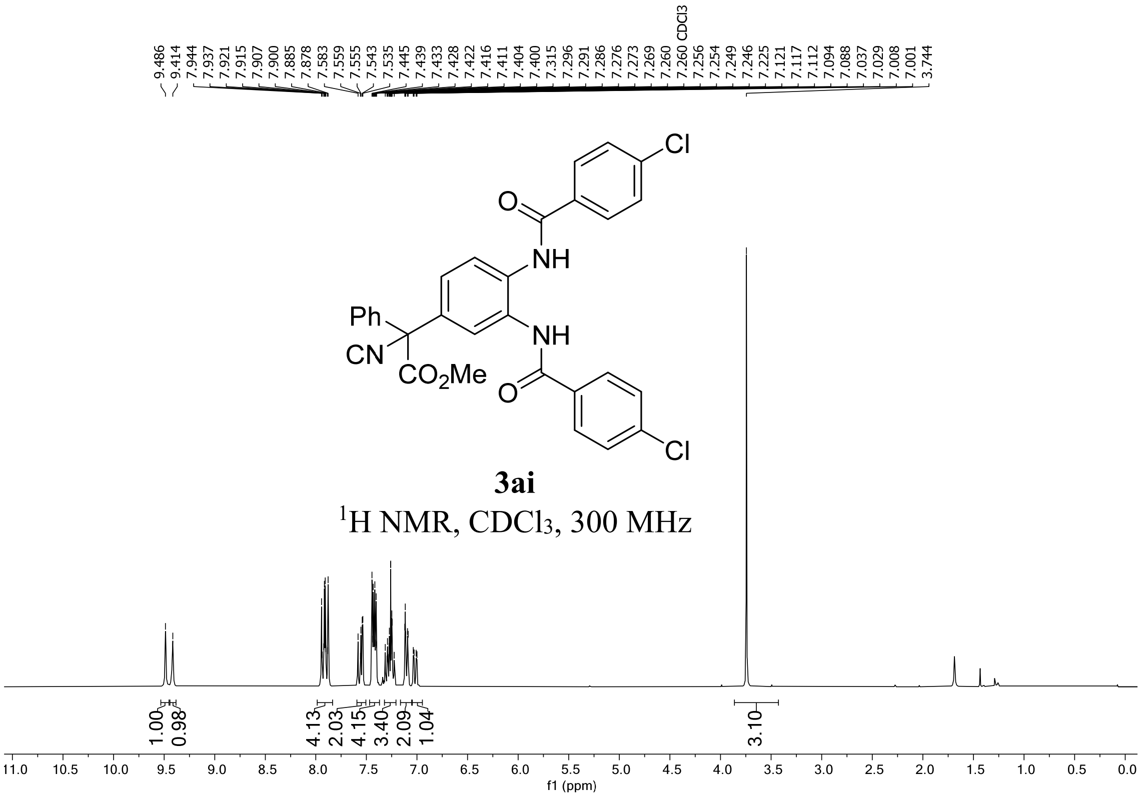

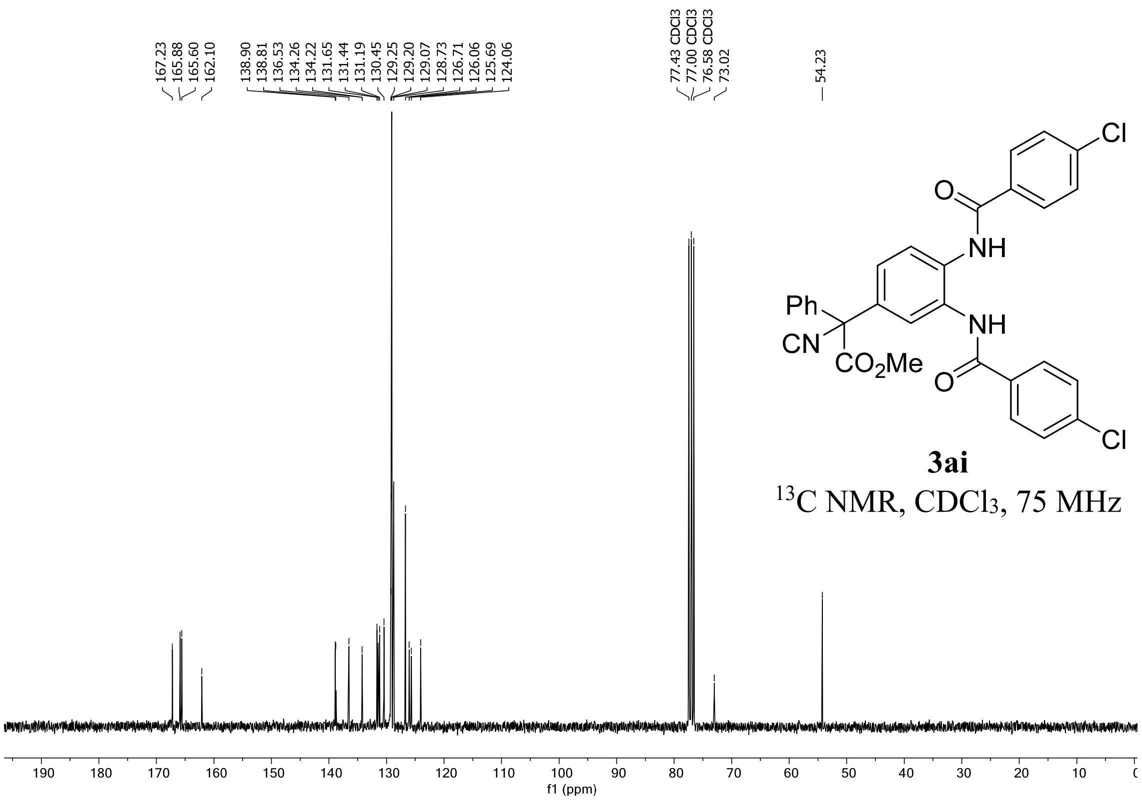

Methyl 2-(3,4-bis(4-chlorobenzamido)-5-methylphenyl)-2-isocyano-2-phenylacetate (3aj)

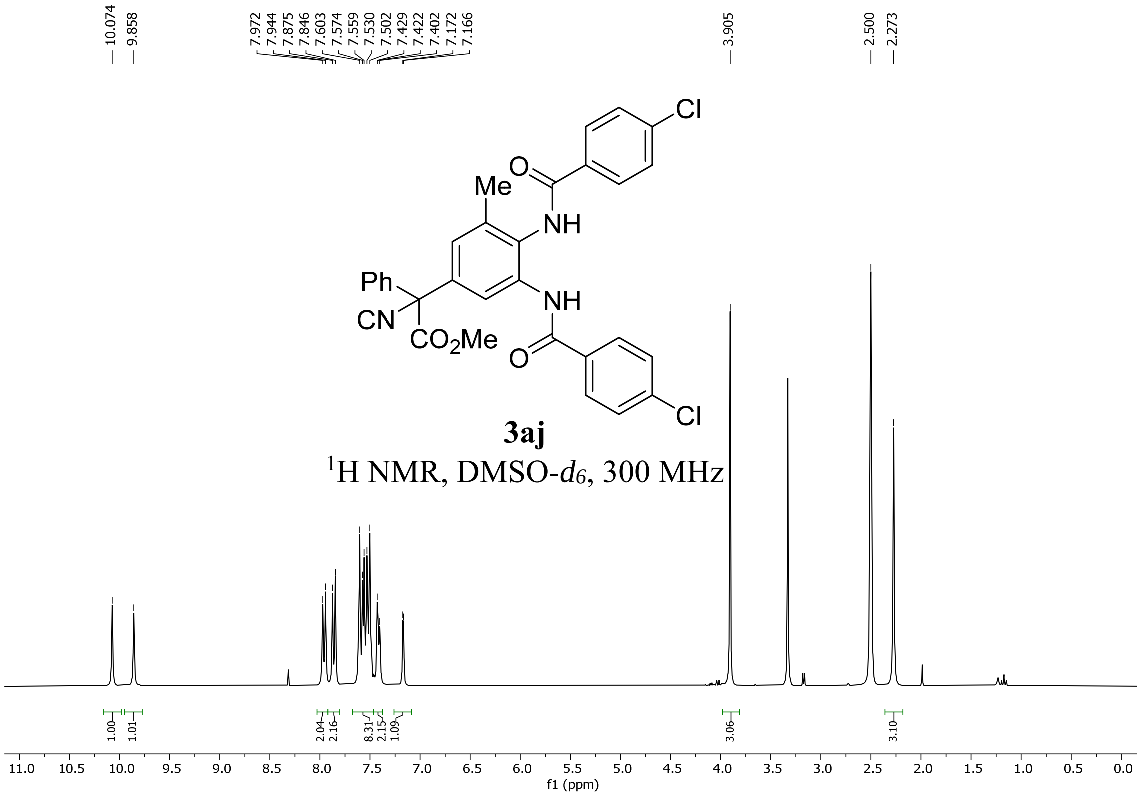

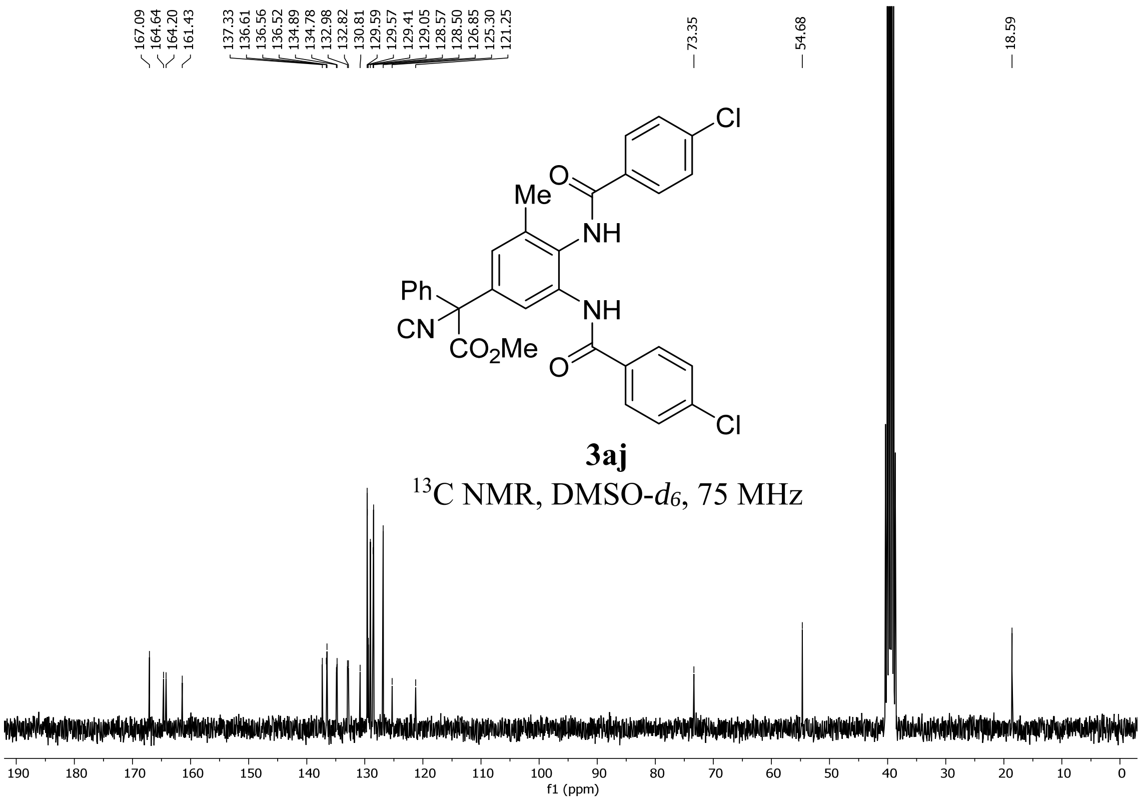

Methyl 2-(4,5-bis(4-chlorobenzamido)-2-methylphenyl)-2-isocyano-2-phenylacetate (3ak)

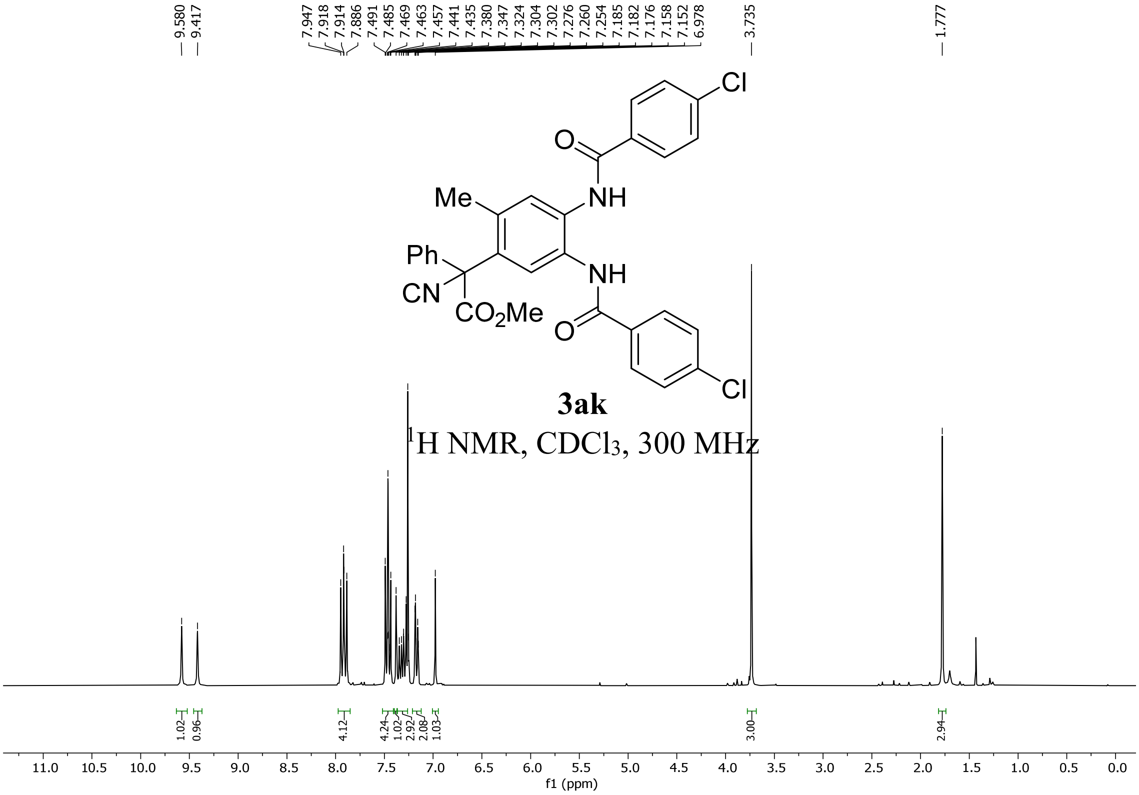

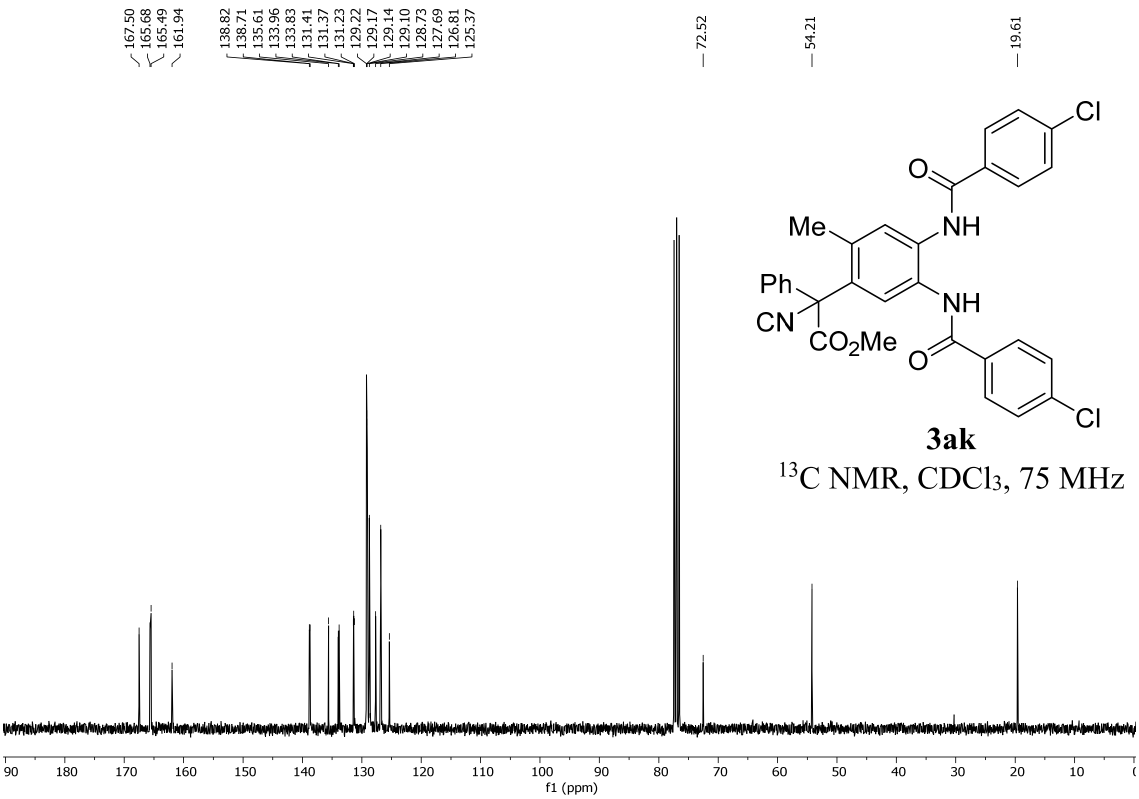

Methyl 2-(3,4-bis(1-naphthamido)phenyl)-2-isocyano-2-phenylacetate (3al)

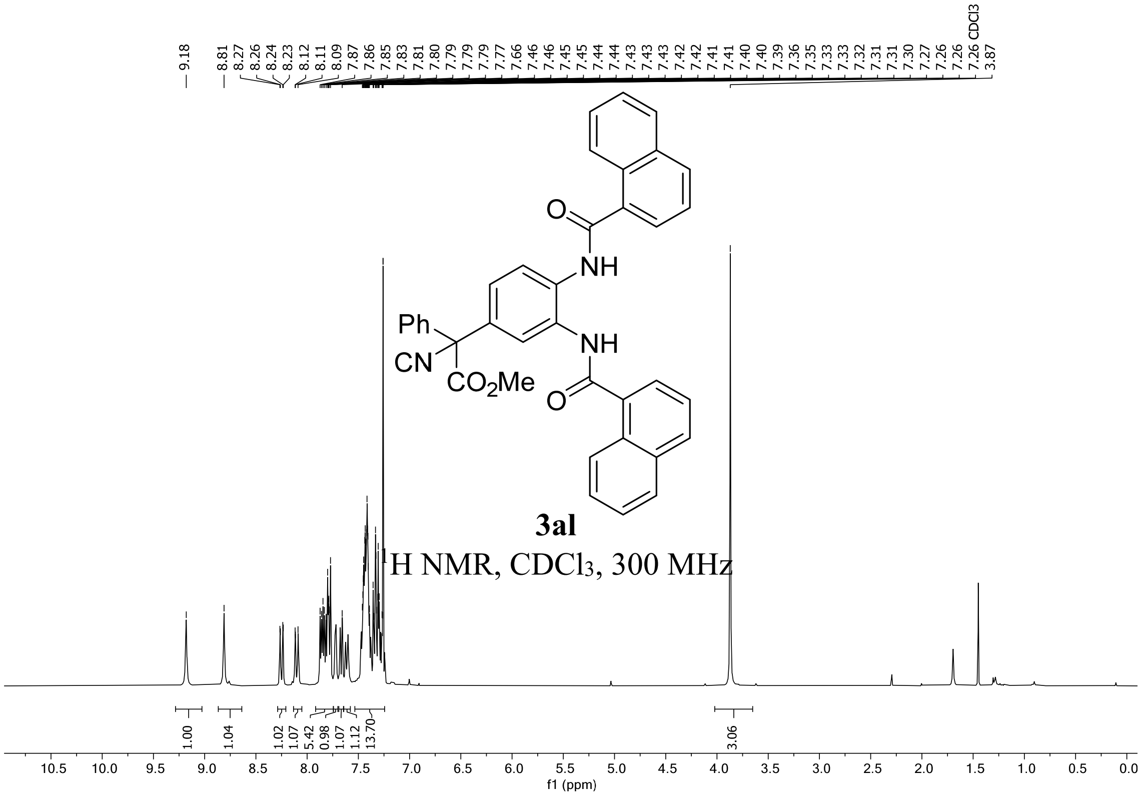

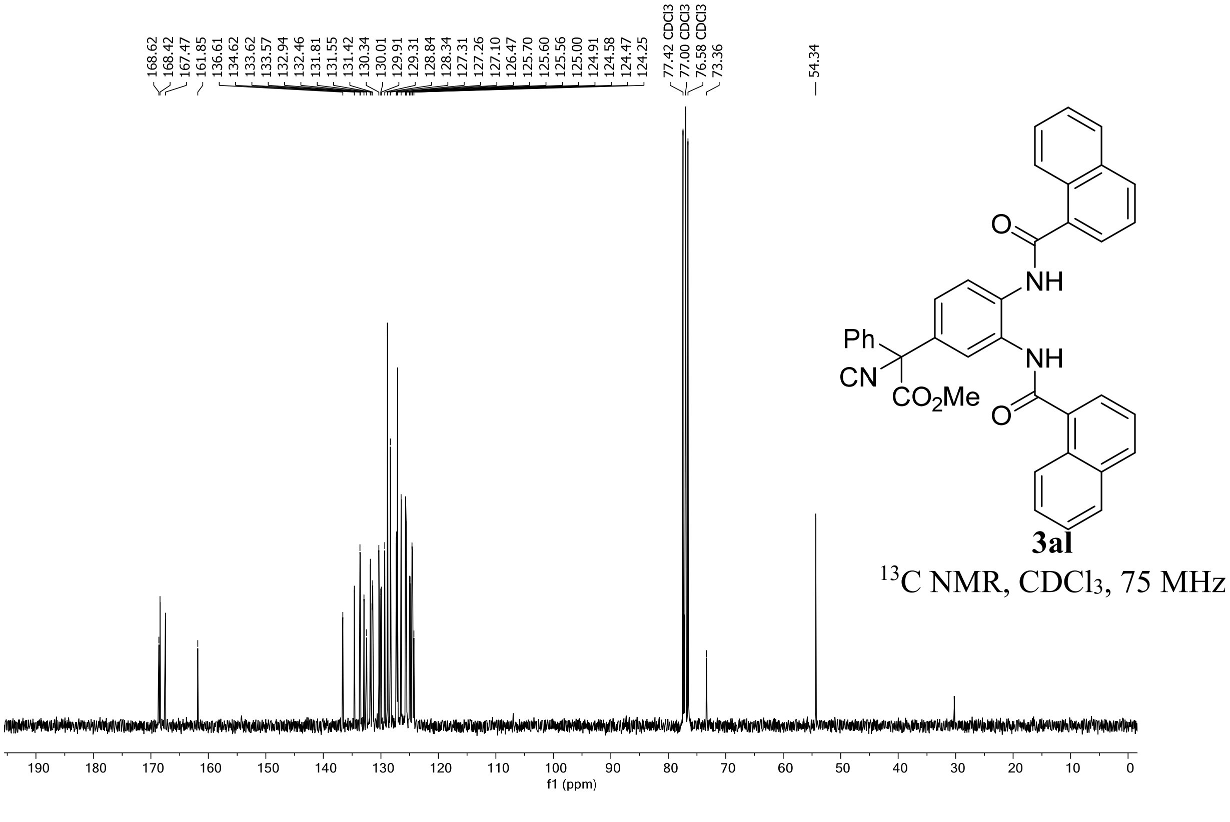

Methyl 2,2-bis(3,4-bis(benzamido)phenyl)-2-isocyanoacetate (3ha)

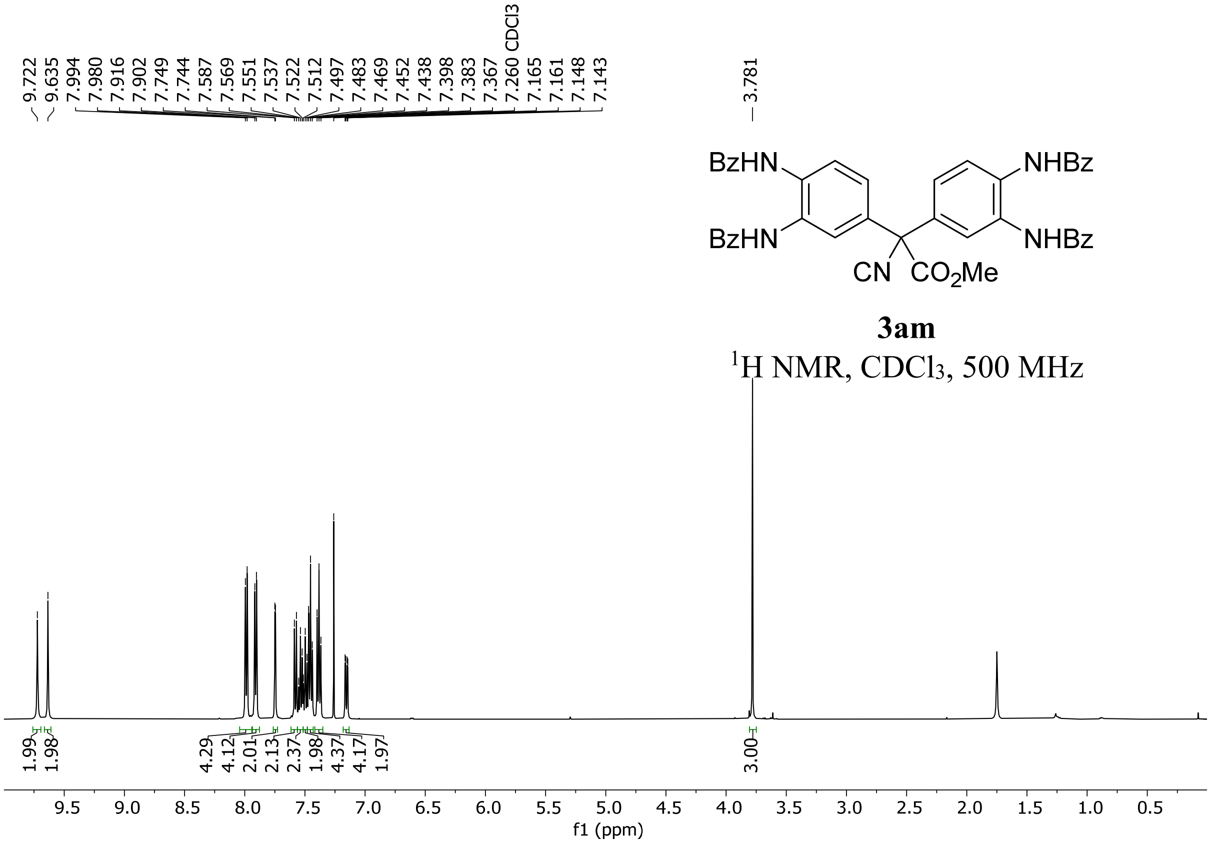

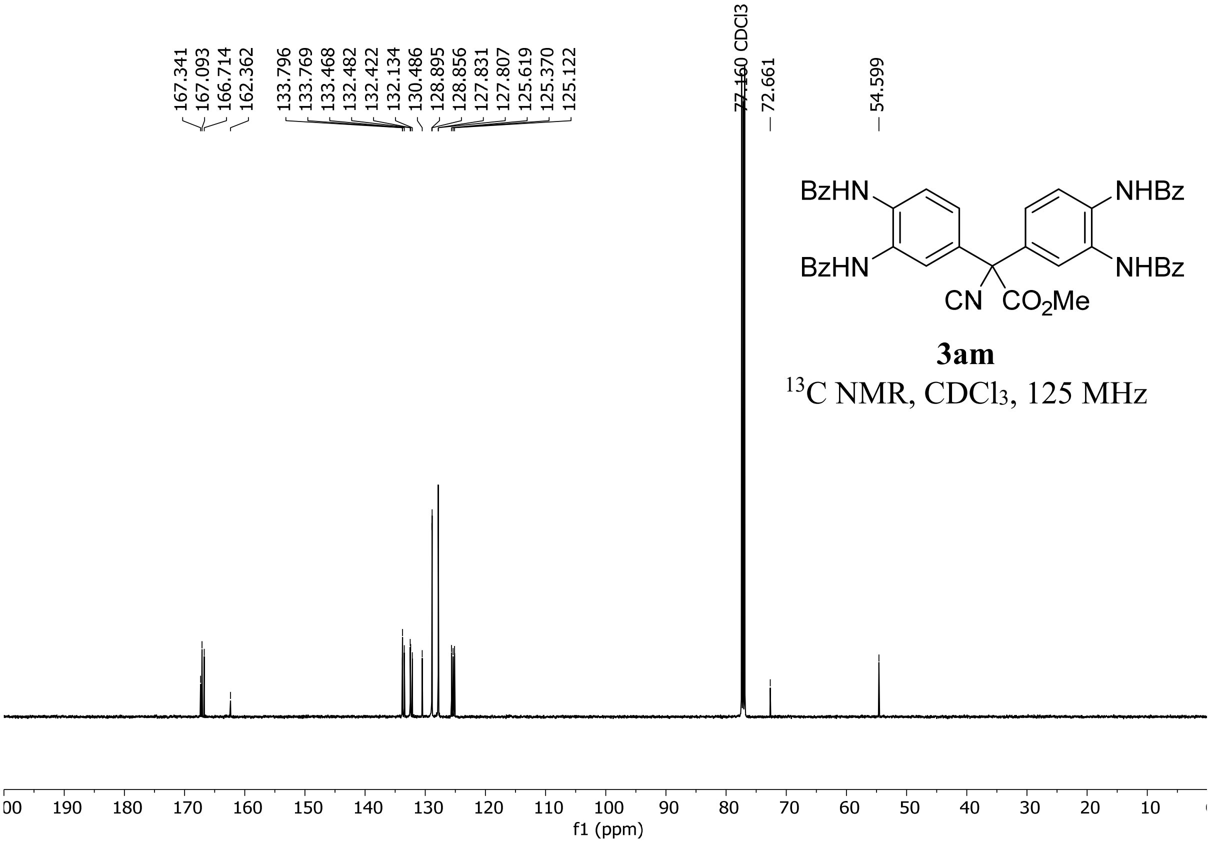

Methyl 6,7-bis(benzamido)-4-phenyl-4H-benzo[e][1,3]oxazine-4-carboxylate (3an)

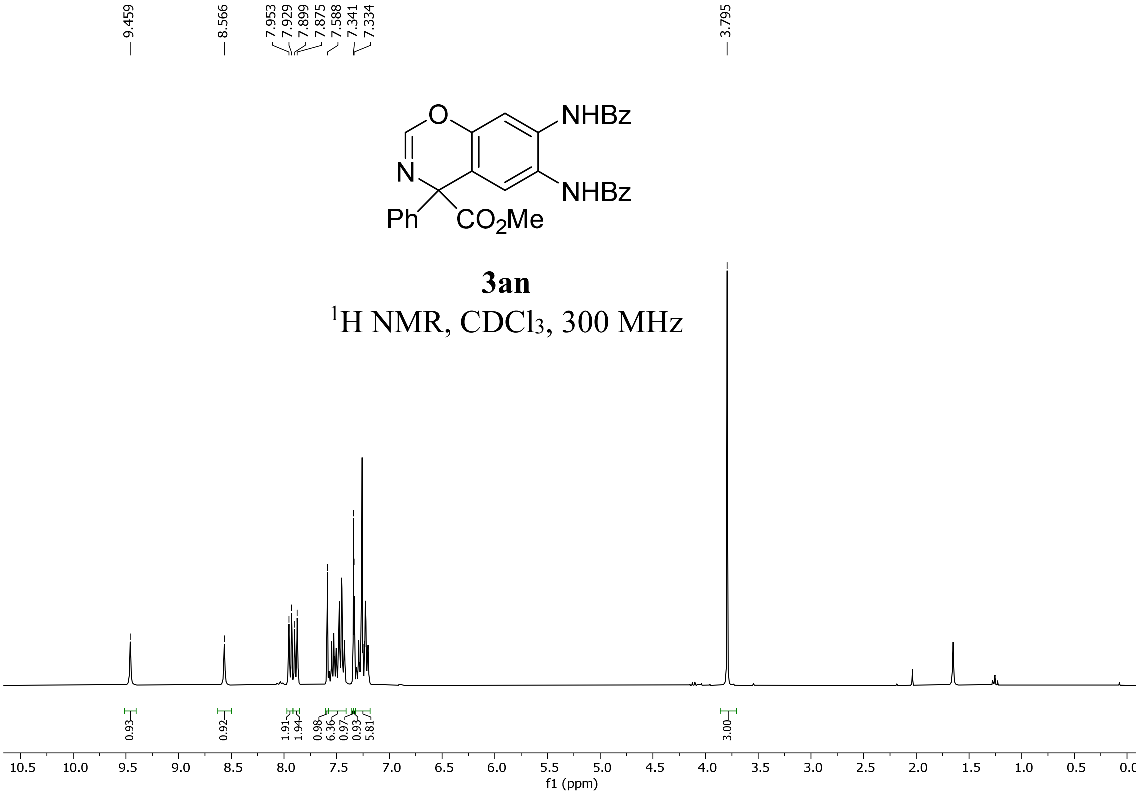

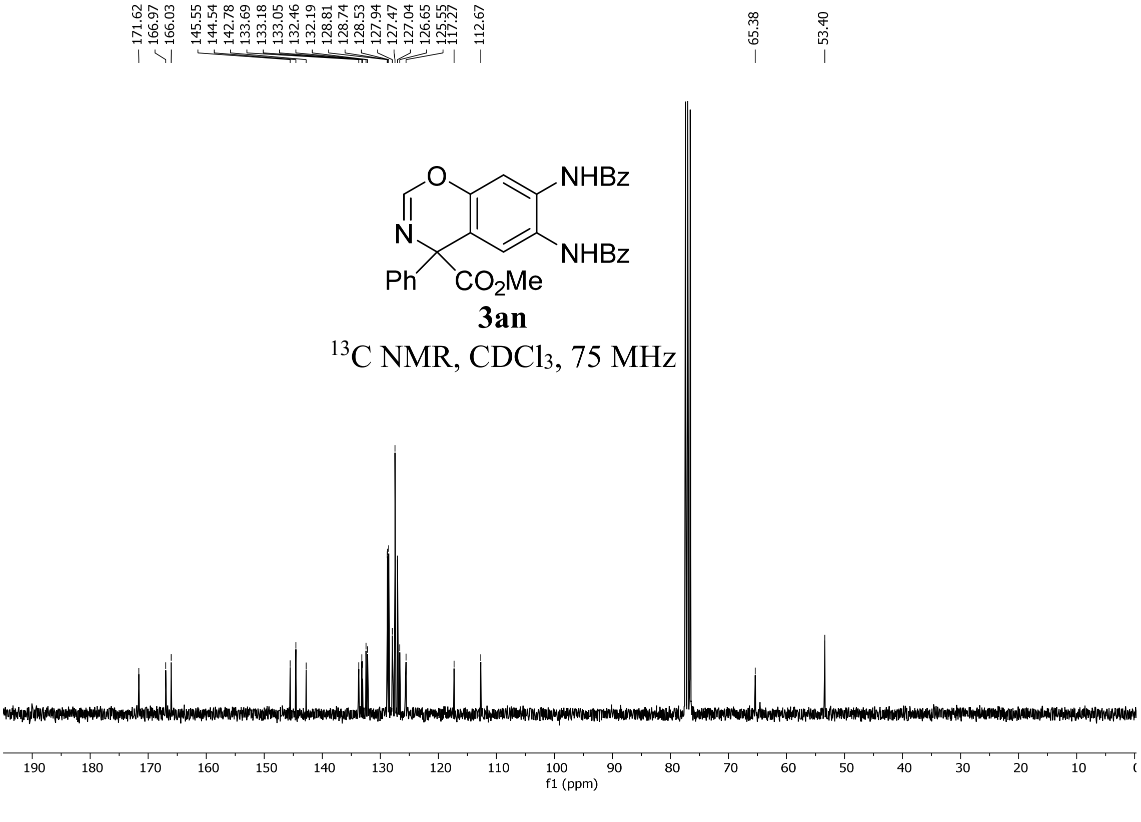

Methyl 6,7-bis(benzamido)-4-(2-nitrophenyl)-4H-benzo[e][1,3]oxazine-4-carboxylate (3fn)

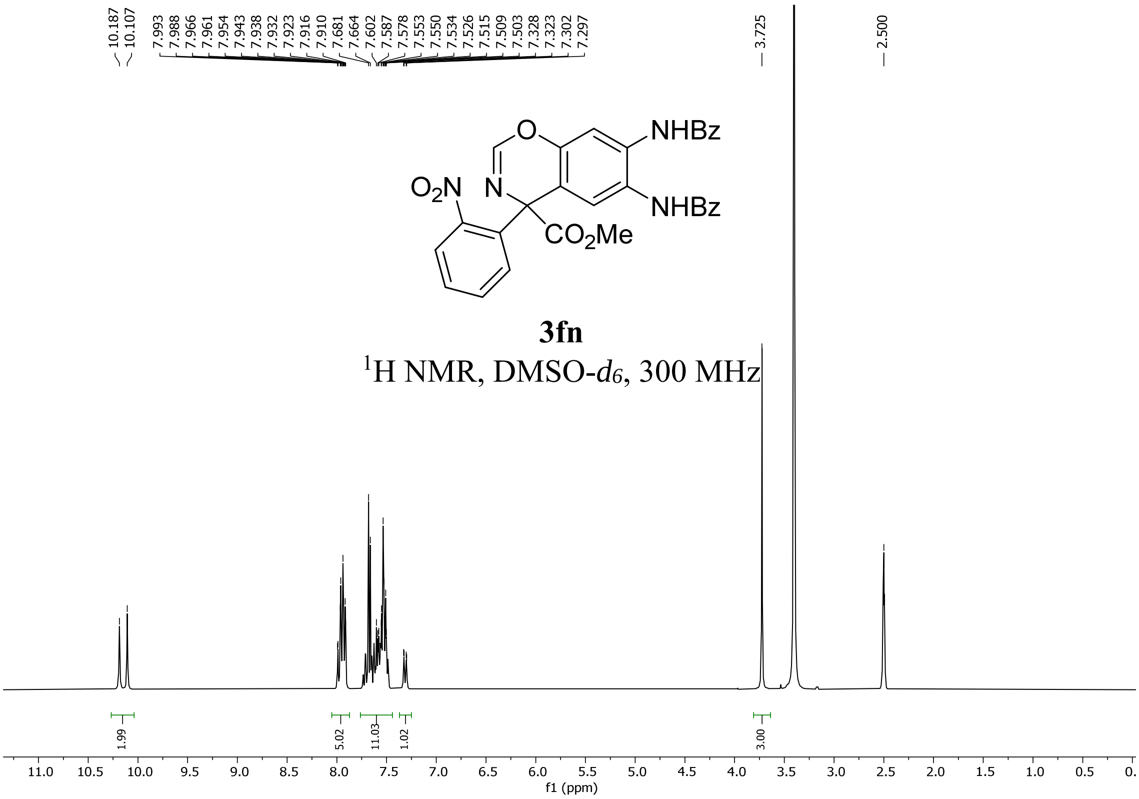

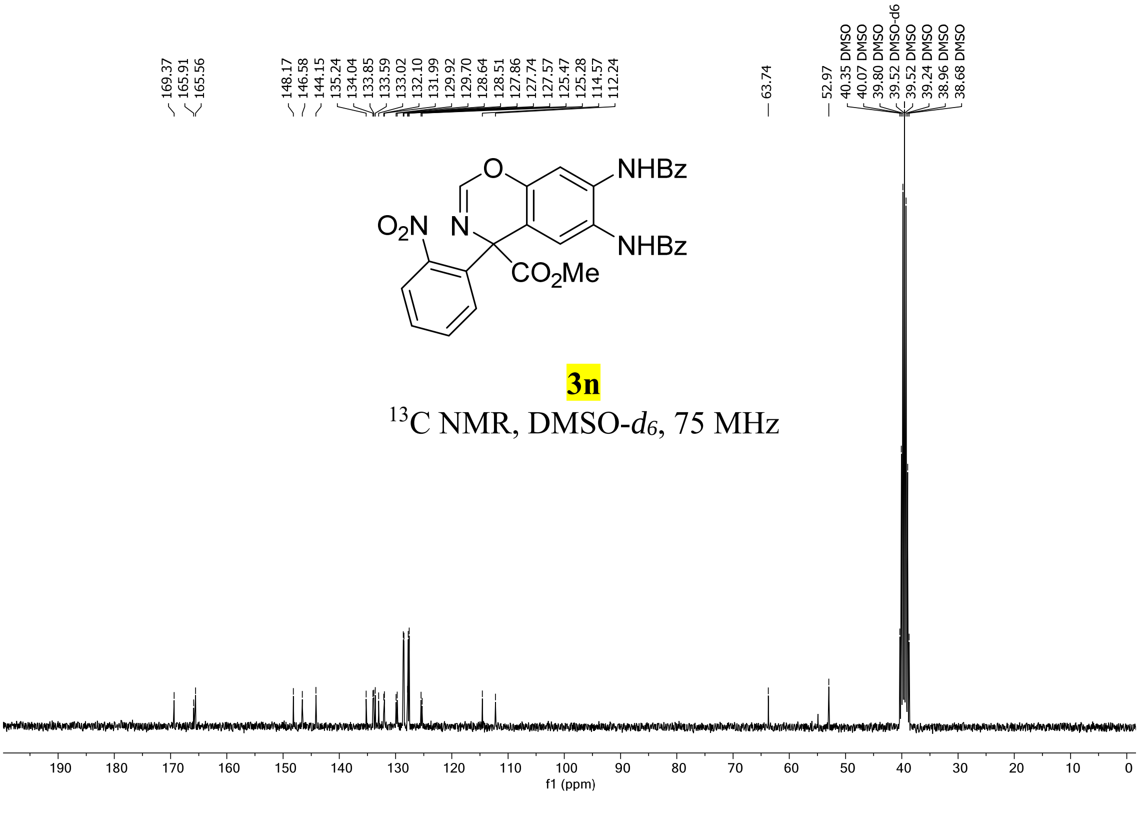

Methyl 2-amino-2-(3,4-bis(benzamido)phenyl)-2-phenylacetate (4aa)

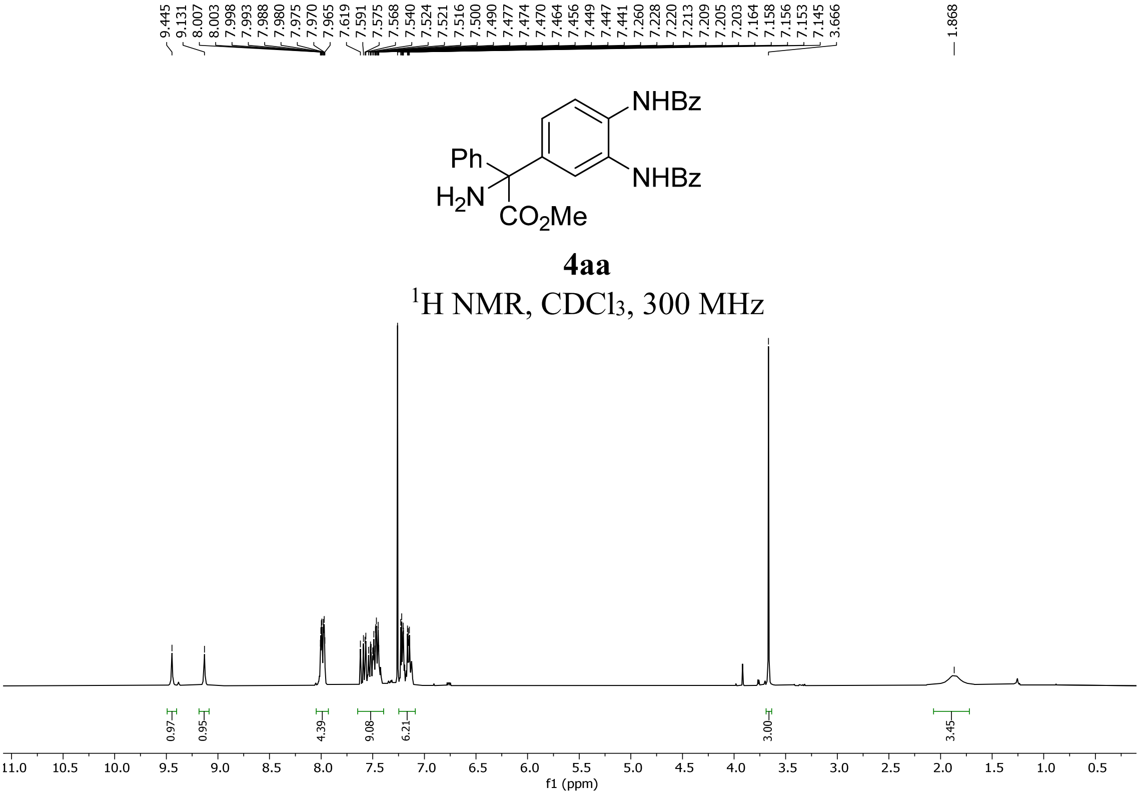

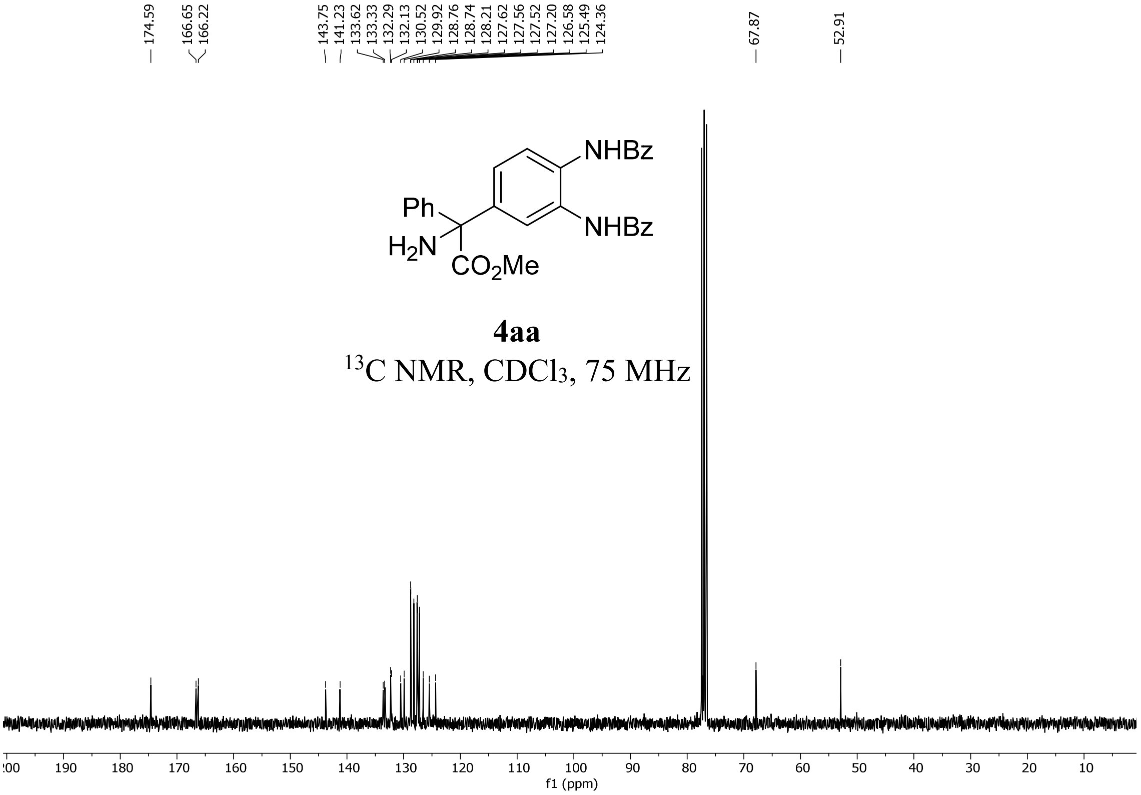

tert-Butyl 2-amino-2-(3,4-bis(benzamido)phenyl)-2-phenylacetate (4ba)

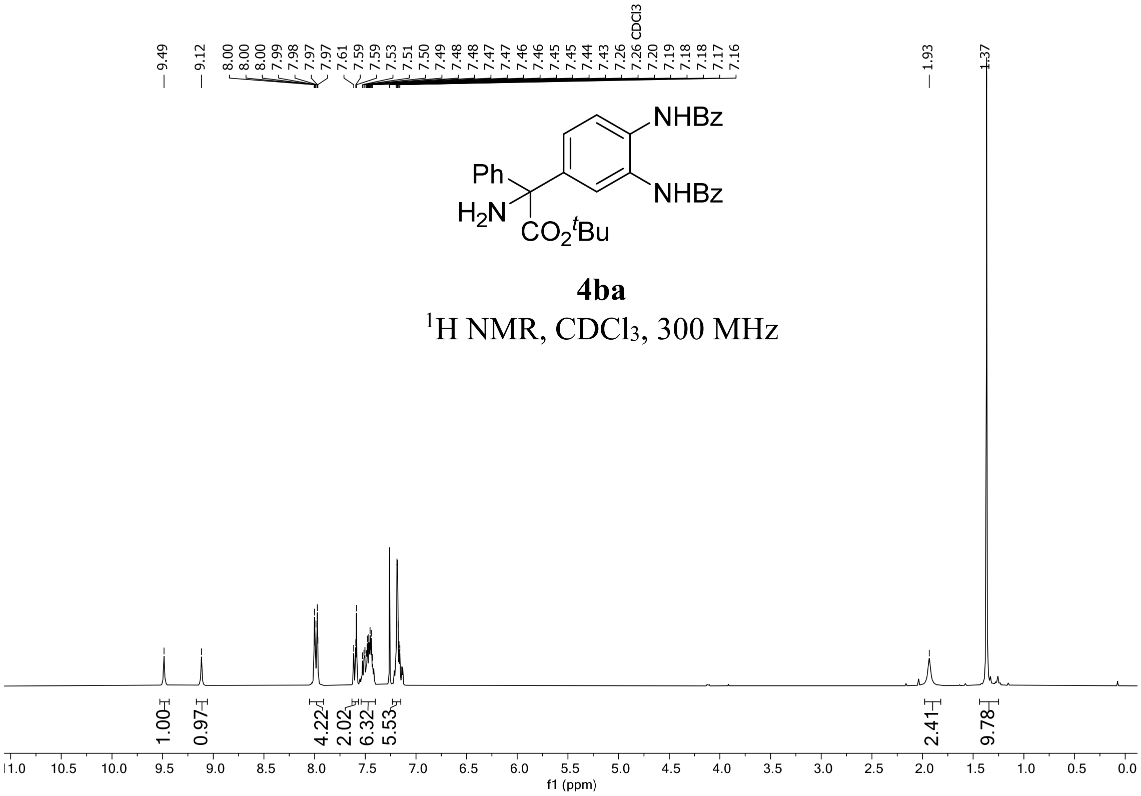

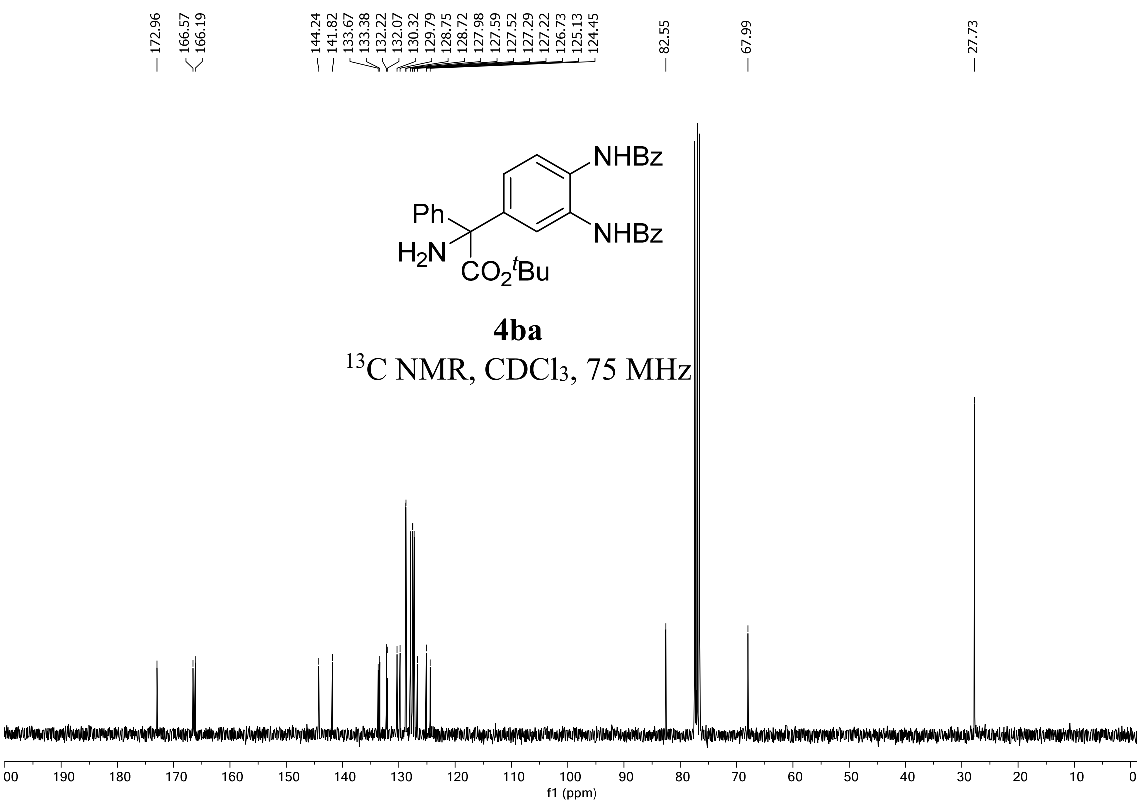

Methyl 2-amino-2-(3,4-bis(benzamido)-5-methylphenyl)-2-phenylacetate (4ab)

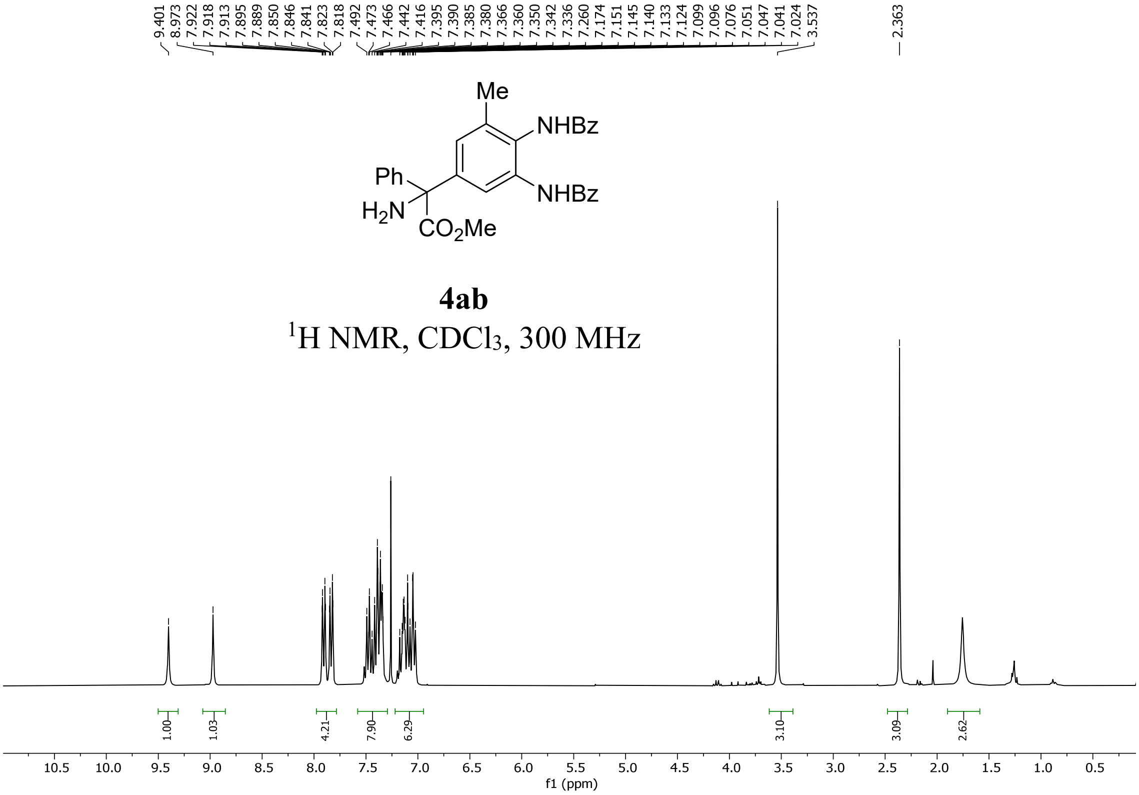

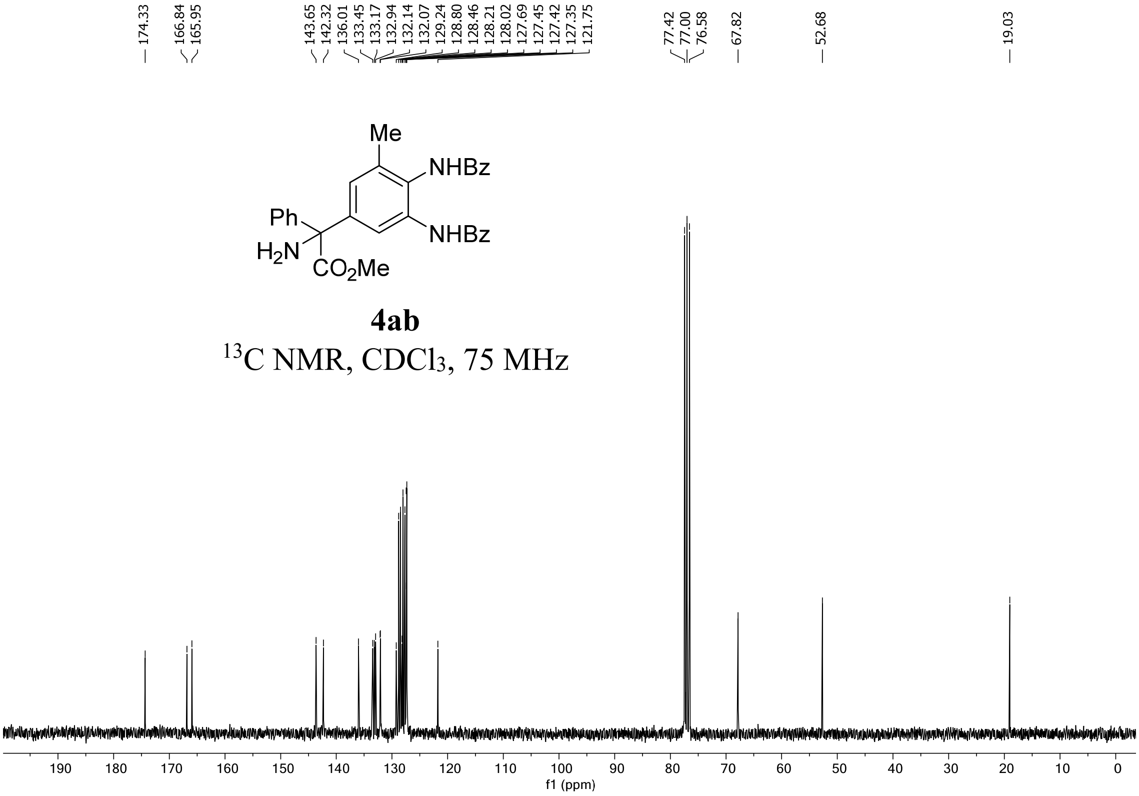

Methyl 2-amino-2-(4,5-bis(benzamido)-2-chlorophenyl)-2-phenylacetate (4ae)

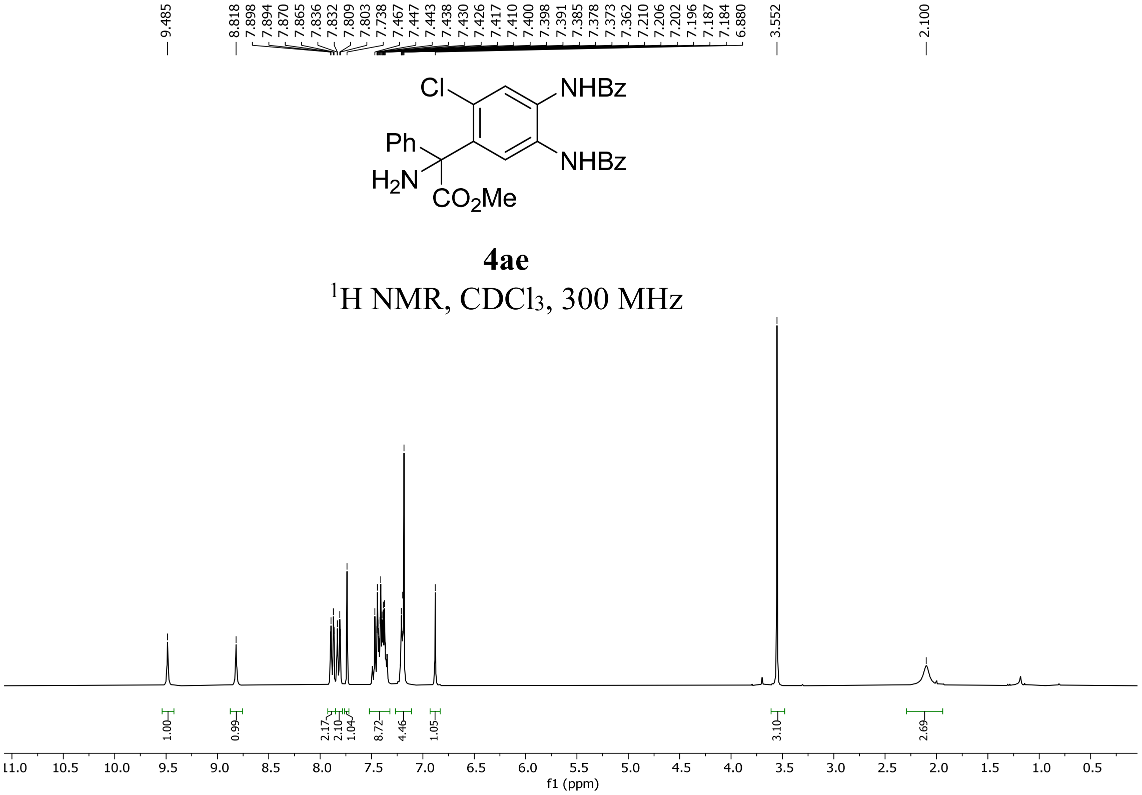

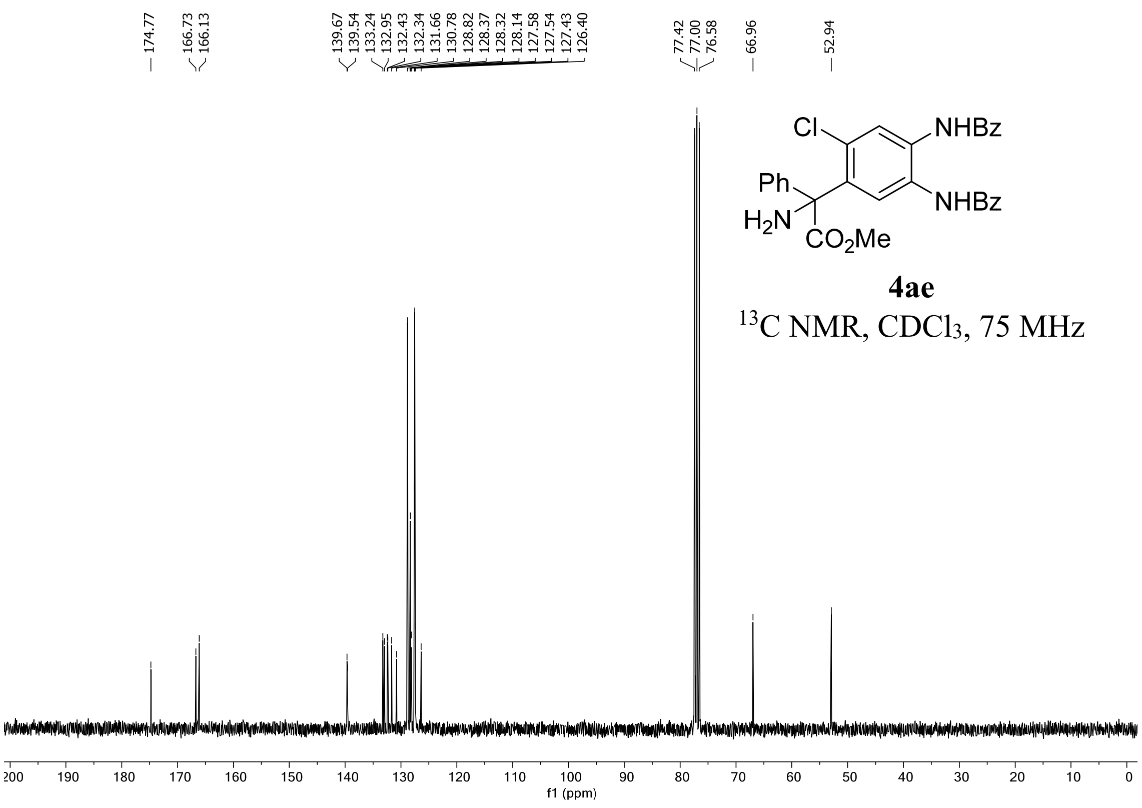

Additional Experiments

1. Reaction in the presence of TEMPO

The reaction catalyzed by Ag_2O was carried out in the presence of 1 equivalent of radical scavenger TEMPO. The reaction provided the expected product in 72% yield after 1 hour, ruling out the radical pathway as main mechanism.

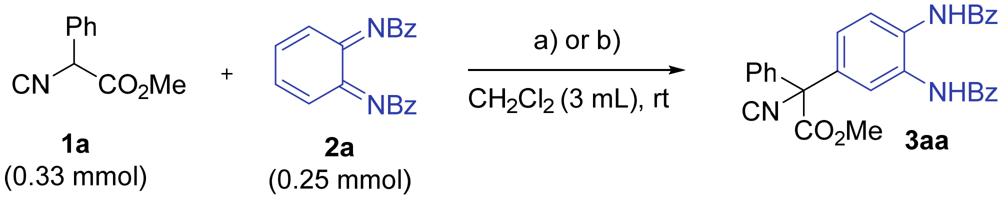

a) Ag_2O (0.0125 mmol) — 99% yield in 1 h
b) Ag_2O (0.0125 mmol), TEMPO (0.25 mmol) — 72% yield in 1 h

2. Attempts to prepare *N*,*N'*-(cyclohexa-3,5-diene-1,2-diylidene)diacetamide.

Oxidation of *N*,*N'*-(1,2-phenylene)diacetamide with lead tetraacetate under the reported conditions lead to a complex mixture. Attempts to oxidize the diacetamide with PIDA (according to a modification of Yang, X.-H.; Li, J.-P.; Wang, D.-C.; Xie, M.-S.; Qu, G.-R.; Guo, H.-M. *Chem. Commun.* **2019**, *55*, 9144–9147) were not successful, the starting material being recovered unreacted.

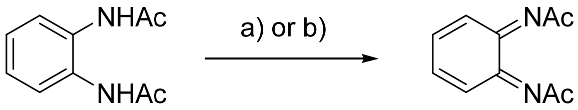

a) $Pb(OAc)_4$ (1.4 equiv), $CHCl_3$ (0.05M), 40 °C, 1 h — complex mixture
b) PIDA (3 equiv), Bu_4NI (2.5 equiv), Et_3N (2 equiv), MeCN (0.1M), 35 °C, 6h — no reaction
